# Donor-Resolved Analysis of HES1-Associated Fibroblast States in Systemic Sclerosis

**DOI:** 10.3390/biomedicines14102360

**Published:** 2026-10-16

**Authors:** Shenyi Fang, Lin Huang, Mingzhu Wang

**Affiliations:** 1 The First Affiliated Hospital of Zhejiang Chinese Medical University, Hangzhou 310053, China; fangshenyi@zcmu.edu.cn; 2 Innovation Center for Medical Basic Research of Autoimmune Diseases, China National Ministry of Education, College of Basic Medical Science, Zhejiang Chinese Medical University, Hangzhou 310053, China; huanglin@zcmu.edu.cn

**Keywords:** systemic sclerosis, myofibroblast, virtual perturbation, gene-regulatory network, Notch, HES1, drug repurposing, nirogacestat

## Abstract

**Background/Objectives:** We examined HES1-associated fibroblast states in systemic sclerosis (SSc) and the rationale for testing upstream γ-secretase inhibition with nirogacestat. **Methods:** We analysed 325,636 skin cells from 230 donors across five non-redundant cohorts using complete computational transcription-factor screens, donor-level activity and trajectory analyses, multiome data and spatial assays. Donors or independent simulations were the inference units, with cohort adjustment and multiplicity correction where applicable. Three independent 200 ns molecular dynamics runs per condition compared nirogacestat-bound and ligand-deleted γ-secretase models. **Results:** HES1 activity was positively associated with model-derived myofibroblast fate in 98.1% of 206 donors (median Spearman’s ρ=0.282; 95% confidence interval [CI], 0.258–0.309). The adjusted SSc–healthy control activity difference was uncertain (0.21; 95% CI, −0.01 to 0.43; Benjamini–Hochberg q=0.061). HES1 ranked 28/107 and 30/109 in the descriptive screens; SMAD3 showed a stronger association with cell fate. Disease-specific communication and contact-enrichment tests did not provide statistical support. The adjusted Visium association was negative; Xenium showed a weak positive mean without section-level corrected support. Nirogacestat was selected retrospectively through mechanistic and translational evidence despite a weak expression-reversal signal and discordant tofacitinib-signature alignment. Simulations showed lower reference-defined pocket root mean square deviation (RMSD) in bound models, with substantial sampling and endpoint-energy uncertainty. **Conclusions:** HES1 activity marks a consistent fibroblast-state association, with uncertain causality and SSc specificity. Nirogacestat is a candidate compound for experimental testing of upstream Notch-pathway perturbation in SSc fibroblasts; the computational findings do not establish efficacy.

## 1. Introduction

Systemic sclerosis (SSc) combines microvascular injury, immune dysregulation and progressive fibrosis of the skin and internal organs. Interstitial lung disease and pulmonary hypertension contribute substantially to morbidity and mortality, and established fibrosis remains difficult to reverse [1]. Myofibroblasts contribute to this process through contractility and excessive extracellular-matrix production [2]. Characterising the regulatory states associated with these cells can help define hypotheses for pathway-directed intervention.

There is experimental rationale suggesting the involvement of Notch signalling in SSc fibrosis. Studies of patient-derived dermal fibroblasts implicated Notch activation in collagen release and myofibroblast differentiation, with HES1 measured as a downstream pathway readout [3]. These experiments support a role for the pathway; they do not establish a causal role for HES1 from its expression alone. γ-Secretase is an upstream intervention point because its cleavage of Notch releases the intracellular signalling domain. Nirogacestat, a γ-secretase inhibitor, reduced fibrotic traits in dermal fibroblasts from recessive dystrophic epidermolysis bullosa, a distinct disease context [4]. In primary endothelial cells from SSc, nirogacestat improved angiogenic readouts in a study of NOTCH4-associated vascular dysfunction [5]. Whether nirogacestat modifies the state of SSc fibroblasts remains an open question.

Single-cell analyses offer complementary ways to examine this question. CellOracle models transcription-factor perturbations from inferred regulatory networks [6]; SCENIC+ links transcription factors, accessible regions and candidate target genes [7]. Donor-level analyses can then separate an association with a fibroblast state from a disease-associated difference. Spatial assays assess whether candidate signalling components share an anatomical context. These measurements address different questions, and agreement among analyses that share input data does not constitute independent replication.

We therefore asked whether HES1 activity is associated with model-derived myofibroblast fate across donors, whether that association is accompanied by SSc-specific activity or communication differences, and how the results position HES1 among alternative regulators. Complete computational screens retained the broader candidate space, while multiome and spatial analyses supplied hypotheses for experimental readouts. We also examined the rationale and limitations of selecting nirogacestat after expression-based drug ranking. The aim was to define a testable Notch-pathway hypothesis with an explicit distinction between observed associations, prior experimental evidence and candidate-specific efficacy.

## 2. Materials and Methods

### 2.1. Skin Datasets, Integration and Annotation

We retrieved deposited RNA-sequencing matrices and metadata for seven candidate GEO datasets: GSE249279, GSE292979, GSE195452, GSE138669, GSE210395, GSE181957 and GSE236111. Participant identifiers, tissue sources and technical-library relationships were reconstructed before donor-level inference. GSE210395 profiled peripheral blood mononuclear cells and was excluded. The 15 GSE181957 samples were excluded as duplicate reprocessing within GSE249279; blood and lung libraries in GSE195452 were also removed. Technical skin libraries from the same participant were merged for inference. The five retained cohorts comprised 325,636 cells from 230 donors (147 SSc and 83 healthy controls [HC]).

The Scanpy workflow filtered cells by counts, detected genes and mitochondrial fraction, normalised library size and log-transformed expression [8]. Annotated matrices were handled in AnnData [9]. Batch-aware highly variable gene selection used cohort as the batch key for the shared embedding, and Harmony used cohort as the integration variable [10]. Neighbourhood graphs, Leiden clusters and UMAP embeddings were constructed from the corrected principal components. Raw-droplet workflows used emptyDrops [11]; droplet ambient-RNA correction used DecontX [12]. Plate-based MARS-seq data were processed separately without droplet decontamination. Dissociation-associated scores were recorded for QC description; they were not regressed from the integrated matrix or included in the reported donor models.

Canonical markers identified 14 major cell types and seven fibroblast states: Myofibroblast, SFRP4+ pro-fibrotic, SFRP2+/DPP4+, Adipogenic, FMO1+/LSP1+, LGR5+ and Inflammatory-like. Marker summaries matched atlas identifiers to full-gene cohort matrices, calculated mean expression and detection within donor–state combinations, and averaged donors with equal weight. The contractile and matrix-associated markers included *ACTA2*, *TAGLN*, *POSTN*, *COL11A1* and *COMP* (Supplementary Figure S1). The Inflammatory-like state comprised 54 cells from one HC donor and was descriptive.

### 2.2. Donor-Level Cell Composition

The independent unit was the deposited donor or participant. For total fibroblasts and each state, counts were divided by all retained skin cells from that donor. Cohort-adjusted beta-binomial logit models used glmmTMB [13]: cbind(count, total-count) ~ condition + cohort. Eight endpoints were planned. SSc-versus-HC odds ratios (ORs), Wald 95% confidence intervals (CIs) and raw *p* values were reported; Benjamini–Hochberg (BH) and Holm adjustments covered the seven estimable tests. The sparse Inflammatory-like endpoint was descriptive rather than estimable.

Sensitivities used alternative denominators, leave-one-cohort-out fits and sum-constrained beta-binomial models in sccomp [14], retaining the condition/cohort design, zero effect threshold, 5% false-positive target and seed 20260723. Details are in Supplementary Methods S1; donor distributions are in Supplementary Figure S7.

### 2.3. Computational Transcription-Factor Perturbations

CellOracle evaluated every TF with non-zero fitted edges under skin-multiome and promoter regulatory priors: 107 and 109 factors, respectively, [6,15]. The signed perturbation score describes the predicted shift against a fixed myofibroblast-directed reference. Rankings and algorithm-reported statistics are descriptive because the archived null contains one randomised-GRN field and neighbouring grid positions are correlated. Full-object reconstructions for HES1, SMAD3 and MEF2C used CellOracle 0.20.0 and were summarised by donor with 10,000-donor-bootstrap intervals, conditional on the fixed fitted network. Supplementary Methods S2 gives the priors, propagation settings, score definition and sensitivity analyses.

### 2.4. Regulon Activity and Model-Derived Cell Fate

CollecTRI regulons and decoupler’s univariate linear model (ULM) estimated TF activity [16,17]. Myofibroblast activity was aggregated within reconstructed donors. HES1, SMAD3 and MEF2C disease contrasts used activity ~ condition + cohort, with ordinary least squares, HC3 robust standard errors, and BH/Holm adjustments across the three focal factors.

Archived CellRank 2.0.7 myofibroblast fate probabilities were joined to activity by exact cell identifier [18]. The primary analysis required at least 30 fibroblasts per donor. Within-donor Spearman coefficients were summarised by their median, 95% CIs from 10,000 donor-bootstrap resamples, signed-rank and sign tests, and BH/Holm adjustments. Cell-count thresholds, cohort summaries and graph reconstructions excluding HES1 or its 76 fixed CollecTRI targets were sensitivities (Supplementary Methods S3). Fate probabilities are model outputs from cross-sectional data and do not directly observe differentiation.

Adult held-out-cohort models evaluated the broader regulon panel, using participant-level aggregation. Imputation, scaling, model fitting and hyperparameter selection were confined to training cohorts; the shared feature universe reflected activity availability across cohorts. The design is described in Supplementary Methods S4, with discrimination and calibration panels in Figure S5. These analyses address multiregulon discrimination rather than HES1 causality or drug response.

### 2.5. Enhancer Networks and Disease Accessibility

SCENIC+ inferred candidate TF–region–gene links from GSE312129, the same multiome source used for the CellOracle skin prior [7,19]. Archived AUCell summaries lacked donor/condition fields and were descriptive. Disease accessibility used all 9087 fibroblasts from four HC and ten SSc donors, aggregated into donor pseudobulks. edgeR quasi-likelihood models tested 69,152 donor-supported, expression-filtered peaks [20]. Exact-coordinate eRegulon intersections required ten tested regions per scope; median disease log fold changes were compared with 9999 abundance- and length-matched draws. Supplementary Methods S5 describes the network, tested universe and focal-scope eligibility.

### 2.6. Spatial Organisation and Candidate Communication

Visium and Xenium addressed spatial association at different resolutions. Visium comprised 14 donor-labelled sections (10 SSc, 4 HC), while Xenium comprised ten SSc sections; each analysis used one section per donor. The separately specified HES1–myofibroblast programme tests used depth-/composition-/domain-adjusted hexagonal adjacency for Visium and depth-/state-/macro-region-adjusted fibroblast six-nearest-neighbour graphs for Xenium [21,22,23]. Restricted within-section permutations and 10,000-donor-bootstrap intervals retained donor-level inference. Supplementary Methods S6 records target-overlap removal, adjustment sets and sensitivities.

Candidate JAG1/DLL4–NOTCH2/NOTCH3 routes were assessed using donor-resolved LIANA+, spatial COMMOT and shared-mask-edge Xenium contact tests [24,25]. Donor disease contrasts and section-bootstrap contact intervals were kept separate, with correction across four routes. Receiver detection used beta-binomial models in 220 donors with myofibroblasts. Xenium supplied within-SSc contact/localisation tests, without an HC disease contrast. Supplementary Methods S7 and Figures S3, S6 and S24 give the complete route settings, null assumptions, NicheNet context and negative results [26,27].

### 2.7. Candidate Selection and Experimental Structure

The disease-expression query and L1000 compound scores were frozen before a retrospective Notch-mechanism and translational-evidence assessment was added. It considered negative expression-reversal scores, direct Notch-suppression evidence, evidence from human cells obtained from patients with SSc and clinical-development maturity. External response alignment was an annotation rather than an exclusion criterion. The frozen query was unchanged, but the added decision was post-ranking and remains subject to selection bias.

The GSE209635 response signature came from a tofacitinib-versus-placebo SSc study [28]. Tofacitinib is a JAK inhibitor, and so alignment cannot establish nirogacestat response. Prior endothelial experiments in SSc, fibroblast experiments in another disease, and desmoid-tumour clinical evidence were retained at their respective scopes [4,5,29].

PDB 8KCT provides the experimentally resolved nirogacestat (O6U) pose in human γ-secretase at 2.60 Å [30]; structural preparation and validation limits are detailed in Supplementary Methods S8.

### 2.8. Plotting and Reproducibility

Plots use recovered source tables where available; preserved vector panels are identified in the provenance record. Matplotlib 3.11.0 generated statistical plots, while PyMuPDF 1.27.2.3 and pypdf 6.11.0 supported vector layouts [31,32,33]. The revision numerical workflows used Python 3.12.5, NumPy 2.4.6, pandas 2.3.3, SciPy 1.16.3 and statsmodels 0.14.6 [34,35,36,37]. The composition-model refit used R 4.4.3 and glmmTMB 1.1.14 [13,38].

Archived spatial-workflow records identify Scanpy 1.11.5, AnnData 0.11.4, decoupler 2.1.4, esda 2.7.0 and libpysal 4.13.0; the archived communication workflow identifies LIANA 1.8.0. Retained setup records specify harmonypy 0.0.10, DropletUtils 1.26.0 for emptyDrops, celda 1.26.0 for DecontX, COMMOT 0.0.3, SCENIC+ 1.0a2, Snakemake 8.5.5 and Cicero 1.3.9. The authors’ contemporary-version confirmation, cross-checked against official release histories, identifies umap-learn 0.5.12 and Python igraph 1.0.0 for Scanpy’s Leiden backend. Author-confirmed GitHub installations, cross-checked against historical repository metadata, specify spacexr 2.2.1 for RCTD, Banksy 1.9.2, nichenetr 2.2.1.1 and sccomp 2.1.34. Tables are editable LaTeX. Supplementary Table S1 documents the versions and their workflow-specific provenance.

### 2.9. Molecular Simulations

Three independently equilibrated 200 ns runs per condition compared nirogacestat-bound (holo) and matched ligand-deleted (apo) models in a lipid bilayer using GROMACS 2026.3 [39]. Structural metrics and common-basis principal component analysis (PCA) assessed model behaviour and sampling; independent runs were the comparison units (n=3 per condition). Membrane-adapted molecular mechanics/Poisson–Boltzmann surface area (MM/PBSA), calculated with AmberTools 26.0 [40], used 151 snapshots per holo run over 50–200 ns and excluded entropy. Supplementary Methods S8, Table S3 and Figures S8–S23 provide model preparation, parameters, sampling diagnostics, structural and exposure analyses, endpoint-energy decomposition and detailed results.

### 2.10. Use of Artificial Intelligence

OpenAI Codex assisted with manuscript preparation and revision. The documented original abstract assistance concerned grammar, clarity, formatting and presentation of author-provided content. During revision, assistance also included drafting and editing the abstract, manuscript and reviewer responses; checking statements against archived code, numerical outputs and cited sources; code implementation and debugging; recovery and indexing of existing results; and preparation and execution orchestration of MD, quality-control, postprocessing and figure-generation workflows. Substantive revisions narrowed causal and disease-specific claims, retained negative findings, disclosed retrospective candidate selection, and replaced superseded MD interpretations with the six-run dataset and its uncertainty. Numerical results were obtained from the executed scientific software and source data described in the Methods. The authors checked the adopted scientific statements, donor/sample counts, statistical units, numerical summaries, figures and citations against these materials, reviewed and approved the adopted AI-assisted content and its disclosed scope, and take responsibility for the final text and interpretation. The desktop product used for this revision was OpenAI Codex version 26.1002.7124.0; this identifies the current application and is not assigned retrospectively to earlier assistance.

## 3. Results

### 3.1. The Skin Atlas Identifies Fibroblast-State Differences

**Figure 1:**
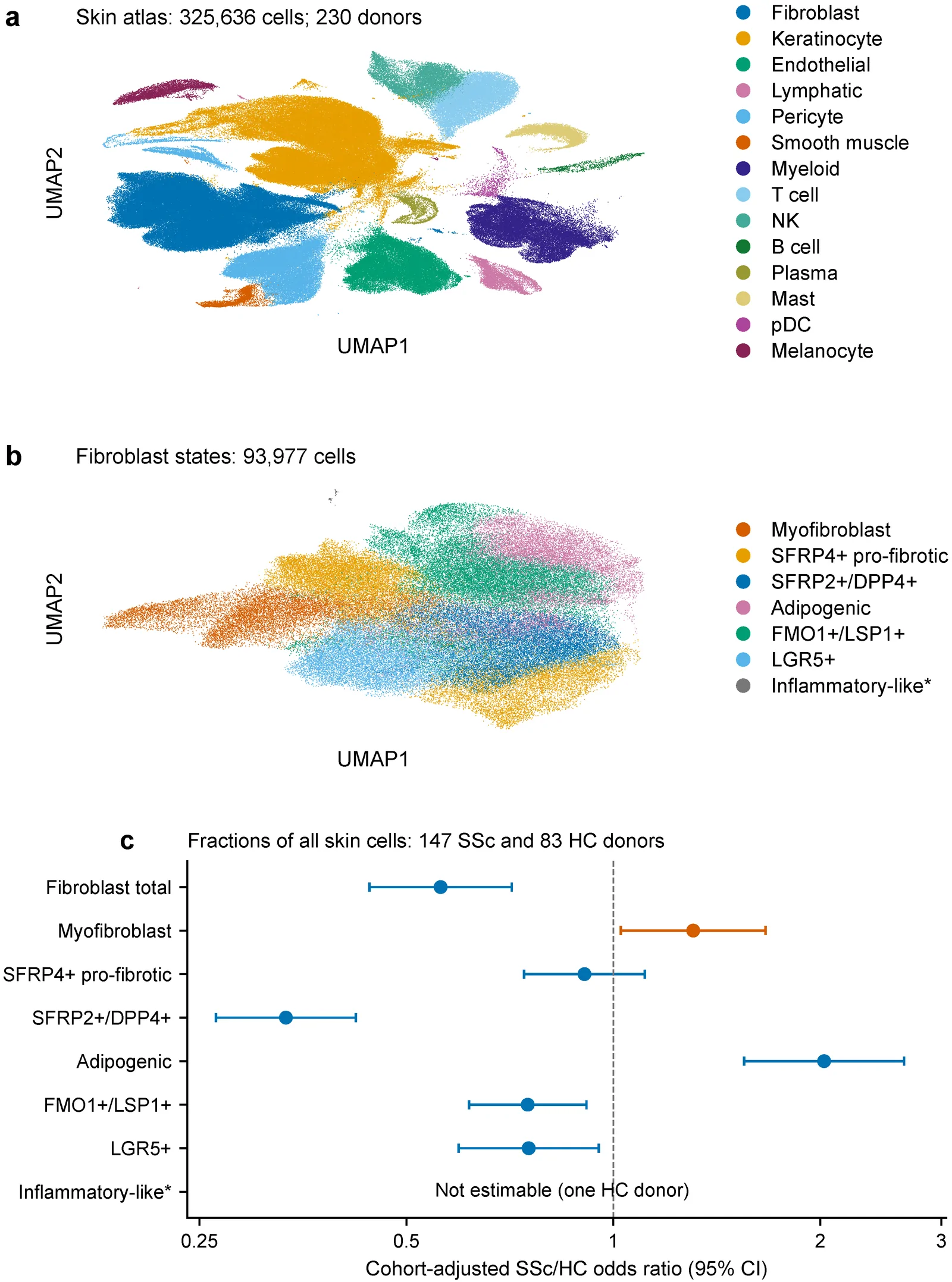
Skin atlas and donor-level composition. (**a**) UMAP of 325,636 cells from 230 donors, coloured by major cell type. (**b**) Seven-state fibroblast annotation of 93,977 cells. (**c**) Cohort-adjusted SSc–HC beta-binomial ORs and 95% CIs, using all retained skin cells as the denominator. Eight endpoints were planned; BH and Holm cover seven estimable tests. The asterisk marks the descriptive Inflammatory-like state, represented by one HC donor; its odds ratio was not estimable. In (**c**), orange highlights Myofibroblasts and blue denotes the other estimable endpoints. The vertical line marks OR = 1. Donor distributions are separated into Supplementary Figure S7.

The non-redundant atlas contained 325,636 cells from 230 donors (147 SSc and 83 HC) across GSE249279, GSE292979, GSE195452, GSE138669 and GSE236111 [2,41]. Harmony provided a joint manifold for annotation (Figure 1a). Fourteen major cell types and seven fibroblast states were retained, with donor-balanced marker evidence (Figure 1b; Supplementary Figure S1). The Inflammatory-like state contained 54 cells from one HC donor and was descriptive.

Relative to all skin cells, total fibroblasts were less abundant in SSc after cohort adjustment (OR 0.56; 95% CI, 0.44–0.71; BH q<0.001; Holm p<0.001). Myofibroblast abundance showed a modest difference whose significance depended on multiplicity adjustment (OR 1.31; 95% CI, 1.03–1.67; BH q=0.036; Holm p=0.061). Adipogenic cells were more abundant (OR 2.03; 95% CI, 1.55–2.65; BH q<0.001). SFRP2+/DPP4+, FMO1+/LSP1+ and LGR5+ cells were less abundant, while SFRP4+ pro-fibrotic cells had an uncertain contrast (Figure 1c; Supplementary Figure S7).

### 3.2. Complete Screens Place HES1 Among Alternative Regulators

**Figure 2:**
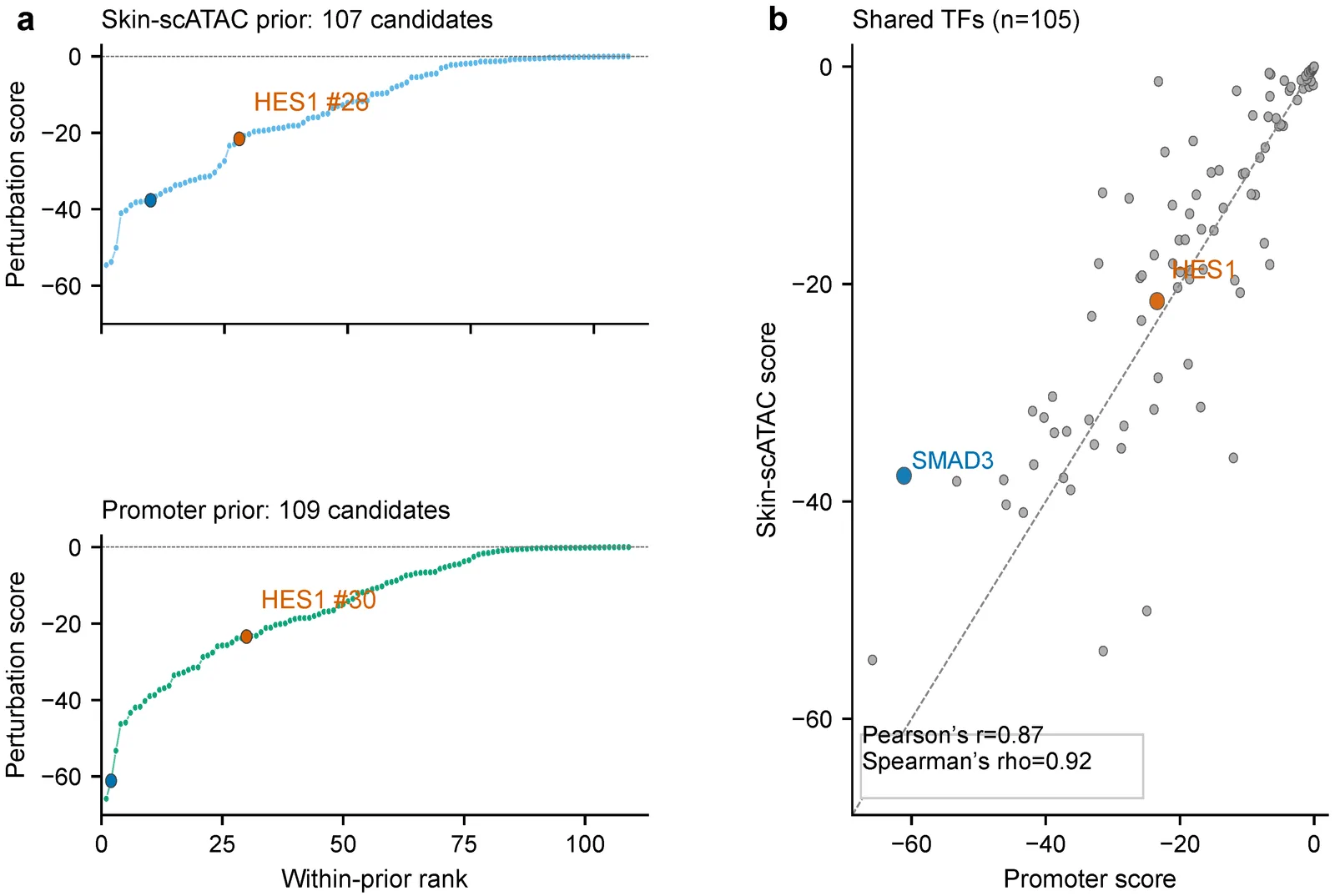
Descriptive regulator rankings under two priors. (**a**) Score profiles for all 107 skin-prior and 109 promoter-prior factors, with HES1 ranks 28/107 and 30/109. More-negative scores indicate a stronger predicted shift against the fixed developmental reference. (**b**) Agreement for 105 shared TFs (Pearson’s r=0.87; Spearman’s ρ=0.92). The dashed line marks equal scores. These rankings are fixed-model summaries; correlated grid positions and a single archived randomised field preclude formal inference from the algorithm statistics. Reconstruction and fixed-field donor summaries are separated into Supplementary Figure S25.

All 107 skin-prior and 109 promoter-prior edge-active CellOracle simulations completed. HES1 ranked 28/107 (score −21.57) and 30/109 (score −23.41). Scores for 105 shared factors were correlated across priors (Pearson’s r=0.87; Spearman’s ρ=0.92; Figure 2a,b). These are descriptive rankings: a single randomised field and spatially correlated grid do not justify inferential interpretation of algorithm-reported *p* or *q* values. HES1’s position does not identify it as the leading unbiased regulator.

The full 29,985-cell reconstruction reproduced the knockout-minus-reference field with maximum numerical discrepancy $1.55\times 10^{-15}$ (Supplementary Figure S25). In the mature-Myofibroblast region, the median HES1 field across 200 donors was −0.00913 (95% bootstrap CI, −0.01755 to 0.00519; BH q=0.305). The paired Myofibroblast-minus-SFRP2/DPP4 lineage difference was −0.02973 (95% CI, −0.05042 to −0.01947; BH $q=6.52\times 10^{-5}$). These summaries are conditioned on a fixed fitted field; they do not demonstrate disease effects or experimental reversal.

### 3.3. HES1 Activity Is Associated with Model-Derived Fate

**Figure 3:**
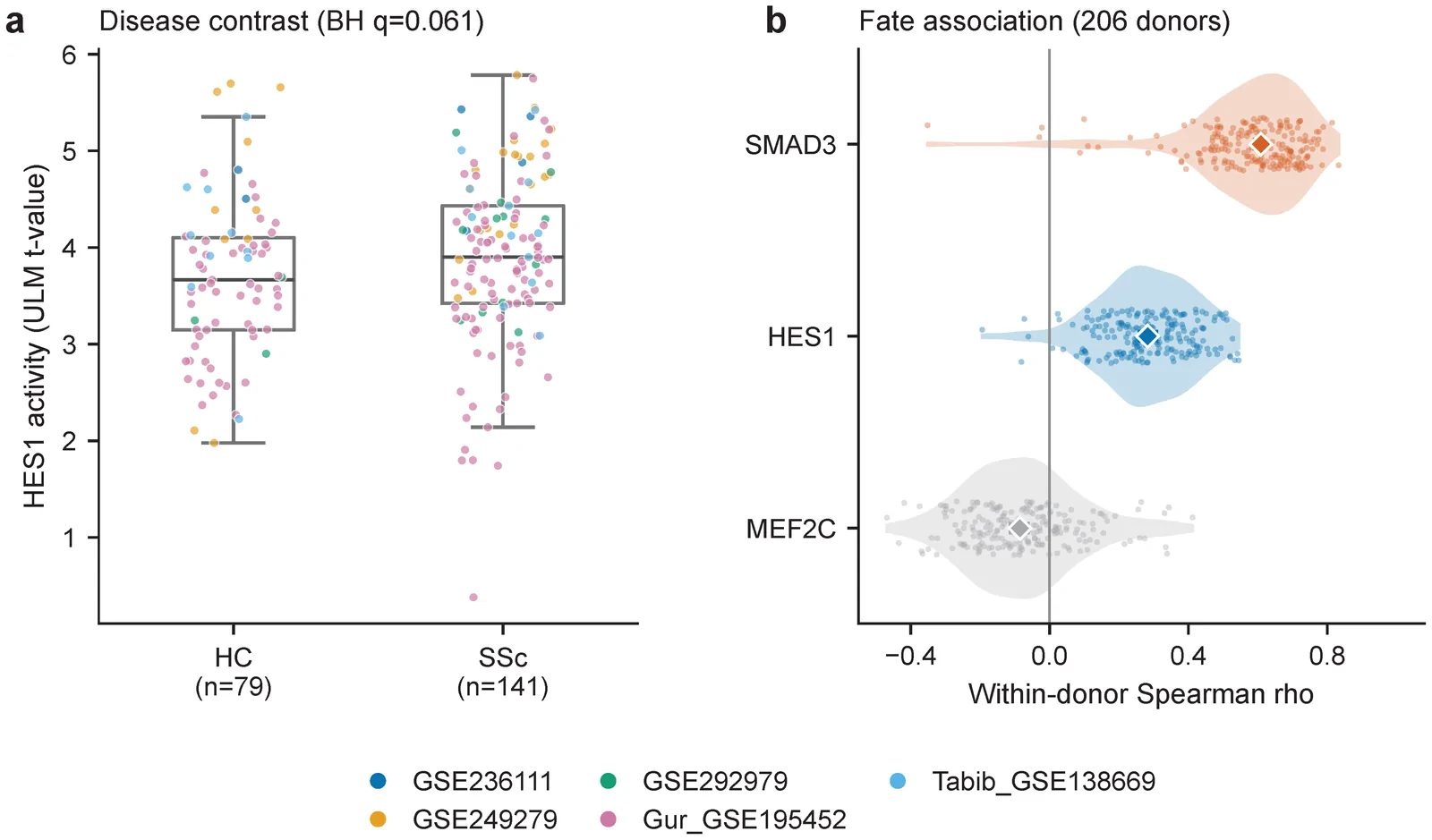
Disease contrast and within-donor fate association. (**a**) HES1 activity in 79 HC and 141 SSc donors, with an uncertain cohort-adjusted contrast. (**b**) Within-donor Spearman coefficients for HES1, SMAD3 and MEF2C in 206 donors with at least 30 fibroblasts. Diamonds indicate medians; bars show donor-bootstrap 95% CIs. Descriptive state-activity summaries and adult multiregulon discrimination/calibration are separated into Supplementary Figures S4 and S5.

Among 220 Myofibroblast donors (141 SSc and 79 HC), the adjusted HES1 activity difference was 0.21 (95% CI, −0.01 to 0.43; BH q=0.061; Holm p=0.061). The CI crossed zero and the contrast missed the 5% FDR threshold (Figure 3a). Unadjusted medians were 3.90 in SSc and 3.67 in HC.

Among 206 donors with at least 30 fibroblasts (132 SSc and 74 HC), median HES1 activity–fate Spearman coefficient was 0.282 (95% donor-bootstrap CI, 0.258–0.309); 98.1% were positive (BH $q<10^{-34}$). SMAD3 was more strongly positive (median ρ=0.609) and MEF2C was negative (ρ=−0.085; Figure 3b). The HES1 direction persisted across cell-count thresholds and graph reconstructions excluding HES1 or its fixed targets. This is consistency within a computational trajectory framework, with causality and SSc specificity unresolved (Table 1).

Eight adult held-out cohorts comprised 554 independent participants. The 668-regulon panel had a median AUROC of 0.856 and a median Brier score of 0.245 (cohort range, 0.169–0.321); removing HES1 yielded a median AUROC of 0.857. The 11-cohort sensitivity comprised 645 participants and 652 shared regulon features, with median full-panel AUROC 0.793. These estimates describe broader-panel discrimination and cohort-dependent calibration, with little incremental discrimination from HES1 (Supplementary Figure S5).

### 3.4. Enhancer Hypotheses Have Limited Focal Disease Coverage

**Figure 4:**
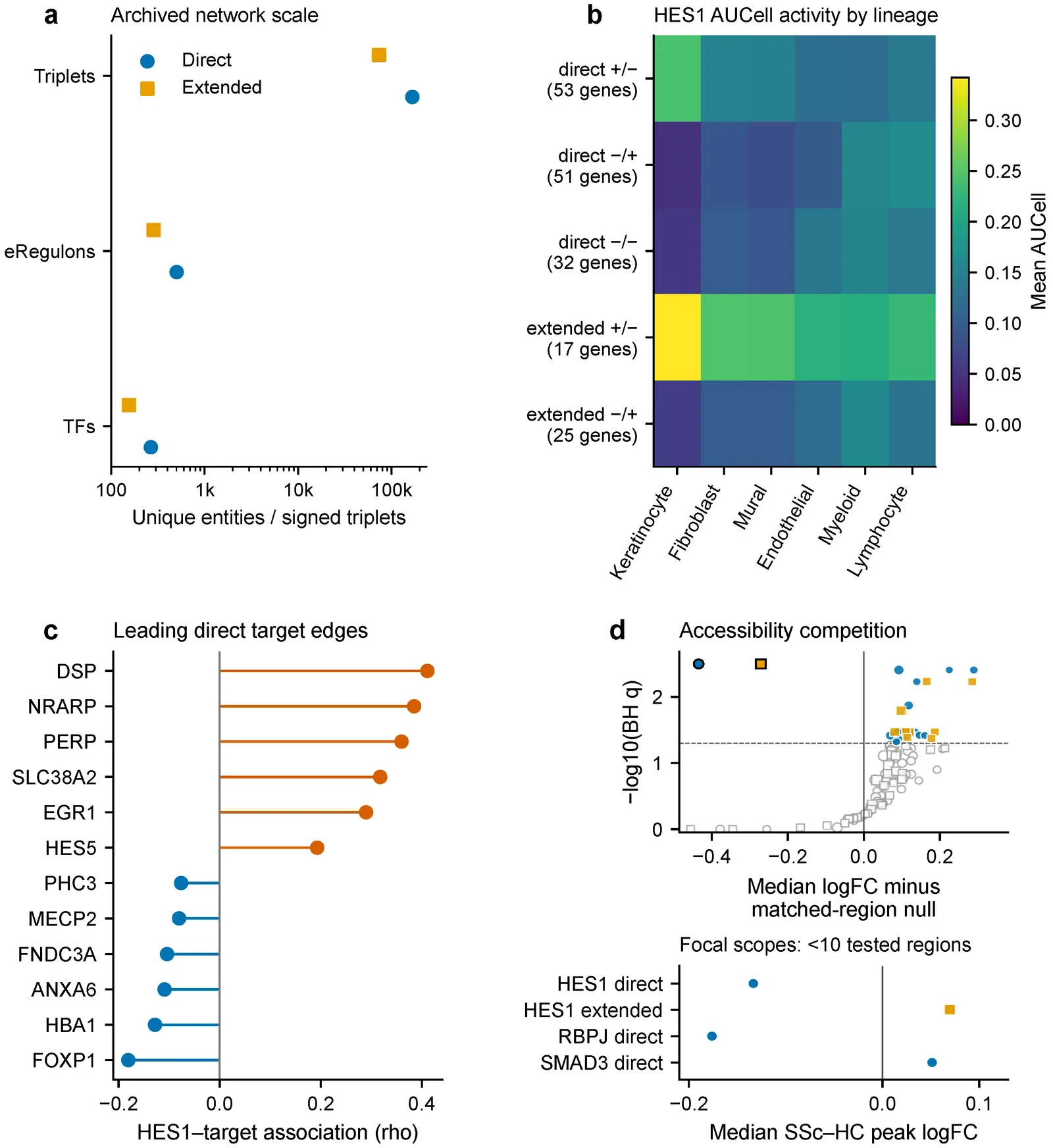
Enhancer-network context and accessibility limits. (**a**) Direct/extended network sizes. (**b**) Mean HES1 gene-based AUCell across lineages. (**c**) Leading direct HES1 target associations; the axis reports inferred TF–target correlation and its sign. (**d**) Disease-accessibility summaries: 118 scopes meet the ten-region gate; HES1, RBPJ and SMAD3 focal scopes have insufficient coverage. In (**d**), the horizontal dotted line marks BH q=0.05, and the vertical lines mark zero on the respective effect axes. Network and disease-testing region sets have different eligibility.

The GSE312129 SCENIC+ network contained 266 TFs, 502 direct and 285 extended eRegulons, and 240,864 signed triplets (Figure 4a). Three direct HES1 eRegulons contained 126 unique target genes and 172 unique archived regions, with ten genes shared between the two negative TF-to-gene sets. NRARP and HES5 links are hypotheses for perturbation, requiring direct regulatory validation. Mean gene-based AUCell scores for the HES1 eRegulons labelled direct +/− and extended +/− were highest in keratinocytes, and so this representation was not fibroblast-selective (Figure 4b,c; Supplementary Figure S2).

Disease accessibility tested 69,152 peaks from four HC and ten SSc donors after a 421,185-region condition union and a 421,173-region donor-support step. Of 336 evaluated TF scopes, 118 met the ten-tested-region gate; BH adjustment covered eligible scopes with finite empirical *p* values, and 24 passed against matched draws. HES1 direct and extended scopes contained five and two tested regions; RBPJ direct and SMAD3 direct scopes contained one and nine, respectively. Each focal scope was below the gate (Figure 4d). These counts refer to the exact-coordinate intersection after condition-union inclusion, donor support and edgeR filtering. Sparse disease-testing coverage limits focal accessibility inference and does not establish biological absence of Notch regulation. SCENIC+ and CellOracle share GSE312129.

### 3.5. Spatial Associations Vary by Assay and Analytical Scale

**Figure 5:**
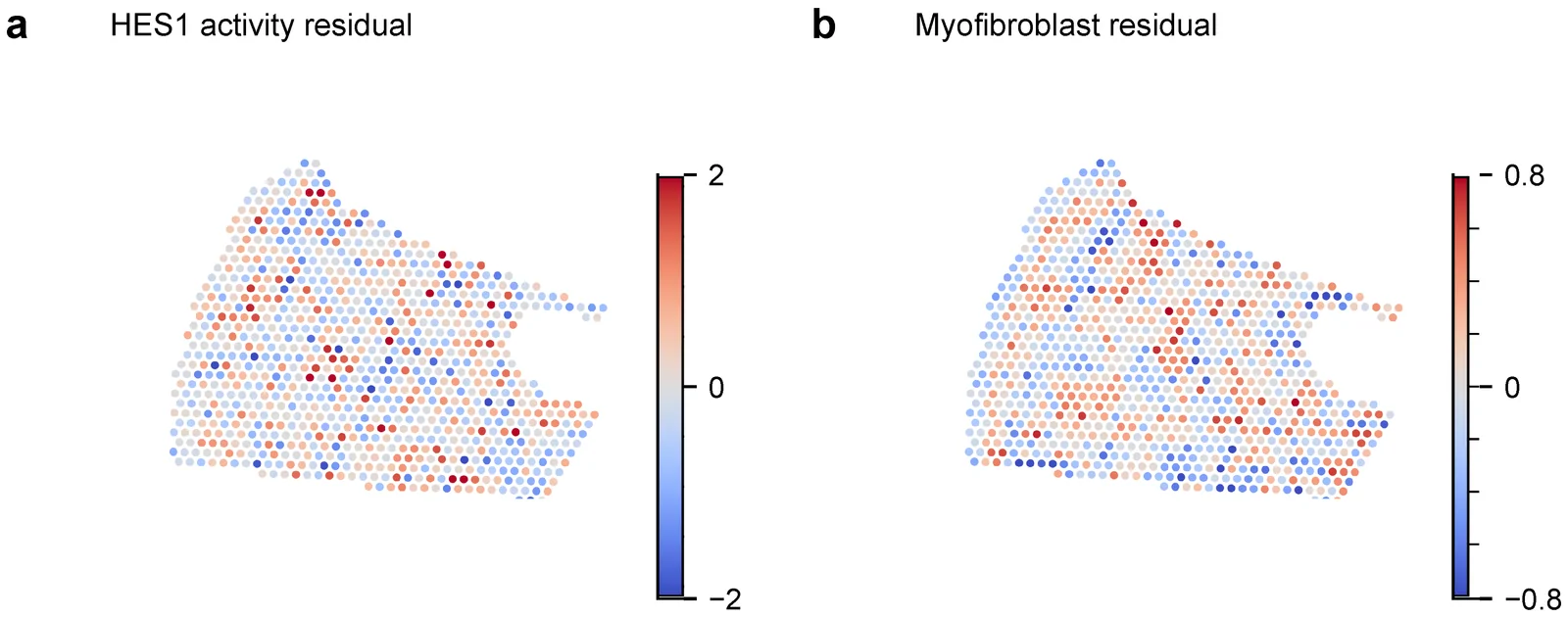

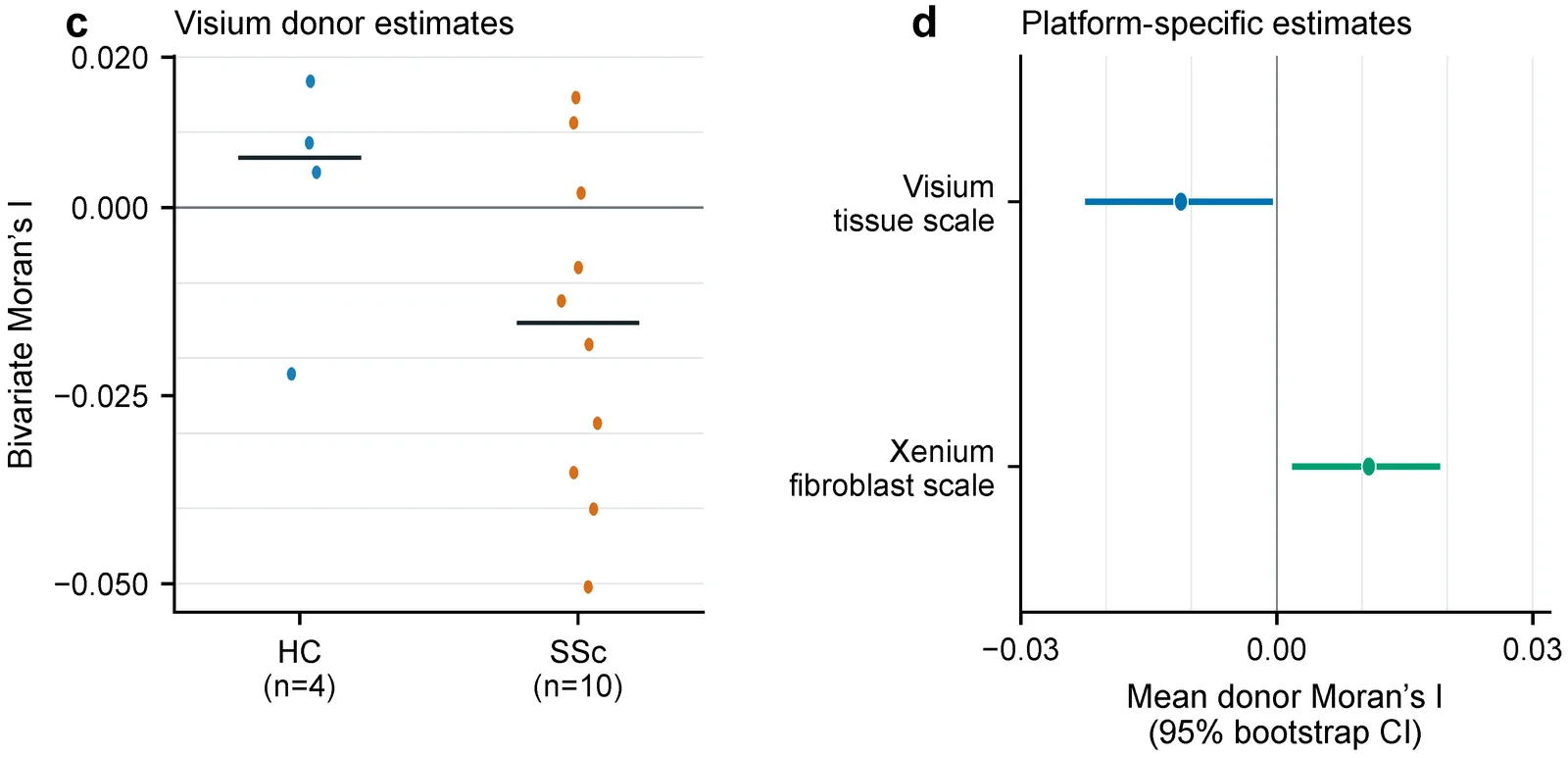
Spatial association at tissue and fibroblast scales. (**a**,**b**) Residual HES1 activity and myofibroblast-programme maps from the same representative Visium section. (**c**) Adjusted bivariate Moran’s *I* for four HC and ten SSc donor-labelled Visium sections; points are sections and horizontal lines are condition medians. (**d**) Equal-donor means and 95% CIs from 10,000 donor-bootstrap resamples for separately specified Visium (n=14) and Xenium (n=10) models. Visium uses hexagonal adjacency; Xenium uses a fibroblast six-nearest-neighbour graph and includes SSc only. Different panels, resolutions and adjustment models preclude treating the two estimates as a common disease-effect test.

For Visium, the mean bivariate Moran’s *I* was −0.01123 (95% donor-bootstrap CI, −0.02247 to −0.00046) across 14 donors after shared-target removal and depth/composition/domain adjustment. Only one section passed BH or Holm, with a negative direction. The separately specified Xenium fibroblast model had a small positive mean (I=0.011; reported donor-bootstrap 95% CI, 0.0018–0.019) across ten SSc donors, while no individual section passed BH or Holm (Figure 5). Different resolutions, panels, graphs and adjustments limit direct comparison. Xenium contains SSc sections only.

**Figure 6:**
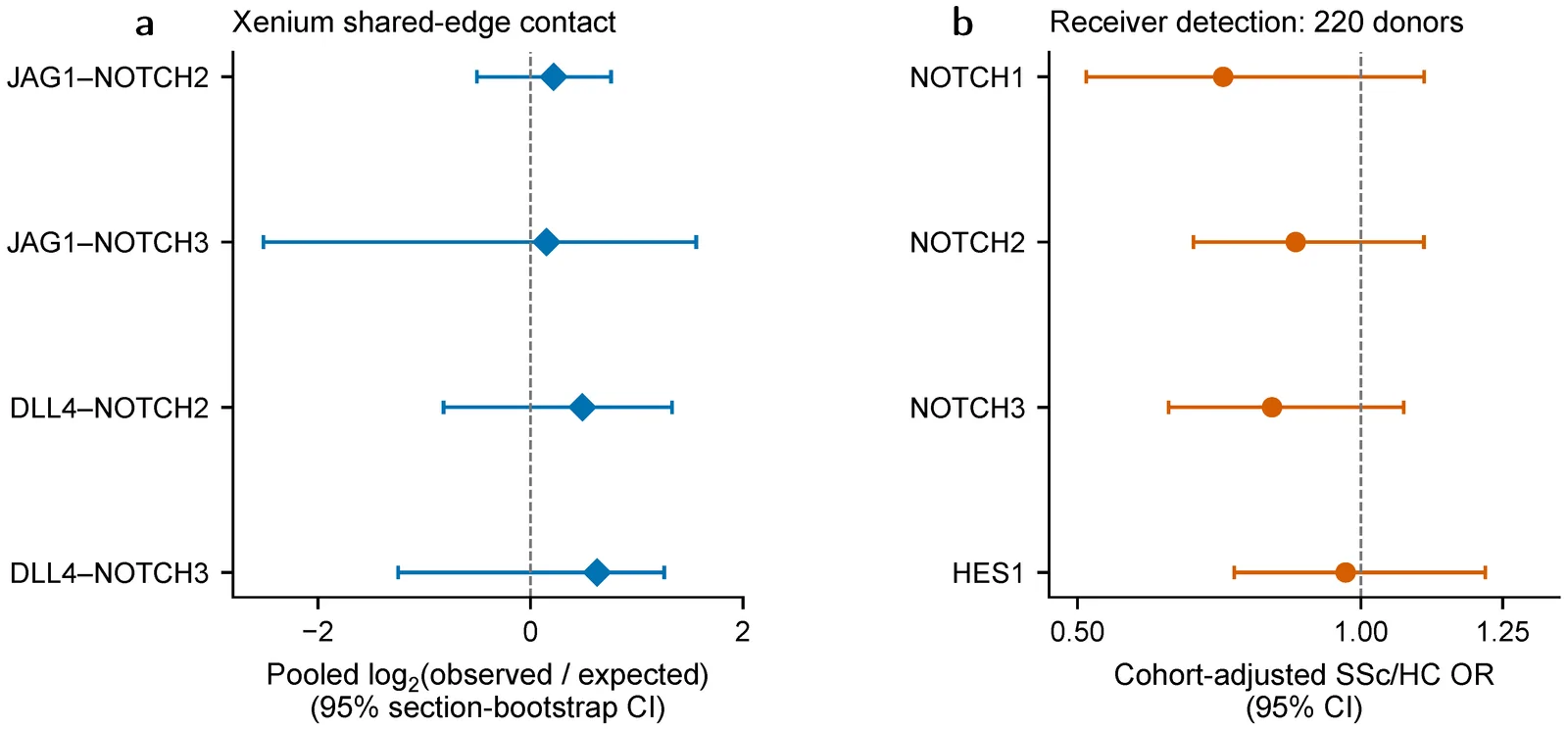
Contact enrichment and disease-associated receiver detection. (**a**) Xenium shared-mask-edge log_2_(observed/expected) enrichment with 95% intervals from 10,000 section-bootstrap resamples in ten SSc-only sections. All intervals include zero (BH q=0.371–0.464); these are within-SSc contact tests. (**b**) Cohort-adjusted beta-binomial SSc–HC receiver-detection ORs and Wald 95% CIs in 220 donors with myofibroblasts. The null references are zero for contact enrichment and one for ORs. BH adjustment covers four routes and four receiver genes as separate families. Neither analysis establishes a disease-associated increase or validates a perivascular Notch route. Donor LIANA+ and Visium disease contrasts are separated into Supplementary Figure S24; auxiliary ranking/localisation is in Figure S6.

All four LIANA+ disease CIs crossed zero (BH q=0.714–0.812; 133 donors for JAG1 and 112 for DLL4 routes). The Visium DLL4–NOTCH2 difference was 0.00085 (95% CI, −0.00002 to 0.00172; exact p=0.0759; BH q=0.304). Xenium shared-edge effects ranged from log_2_(observed/expected) 0.15 to 0.63, all CIs crossed zero, and BH q=0.371–0.464. Receiver detection also lacked significant disease-associated increases: NOTCH1, NOTCH2, NOTCH3 and HES1 ORs were 0.76, 0.88, 0.84 and 0.97 (BH q=0.339–0.812; Figure 6). The contact and receiver tests are shown in Figure 6; full LIANA+ and Visium route results are in Supplementary Figure S24. The tests do not validate disease-specific perivascular signalling. The rankings, localisation and representative contacts remain exploratory (Supplementary Figures S3 and S6).

### 3.6. Nirogacestat Is a Retrospectively Identified Experimental Candidate

Nirogacestat (PF-03084014) ranked 6440 of 13,937 finite L1000 compounds, with primary reversal score −0.103. Its alignment with the external tofacitinib-response signature was discordant (−0.760). RO4929097 had stronger primary reversal (−1.007; rank 3710). The retrospective decision favoured nirogacestat’s mechanism and clinical-development maturity; expression evidence was weak and inconsistent (Table 2).

Prior nirogacestat treatment improved angiogenic readouts in SSc endothelial cells and reduced selected markers of the endothelial-to-mesenchymal-transition in the tested NOTCH4-risk context [5]. This was γ-secretase inhibition, not a selective NOTCH4 perturbation. Fibroblast evidence came from recessive dystrophic epidermolysis bullosa, a different disease [4]. The 8KCT structure resolves O6U in the PSEN1 substrate site, and desmoid-tumour phase 3 evidence establishes development maturity [29,30]. None establishes efficacy in SSc fibroblasts.

### 3.7. Membrane Simulations Provide Structural Context for Nirogacestat

**Figure 7:**
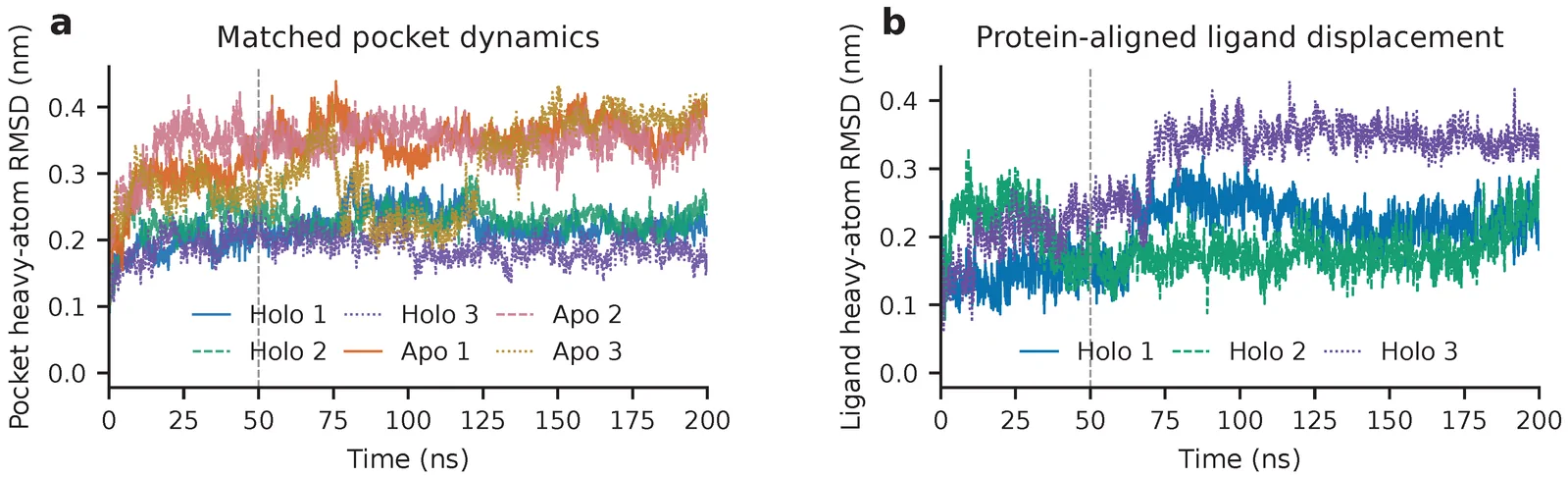

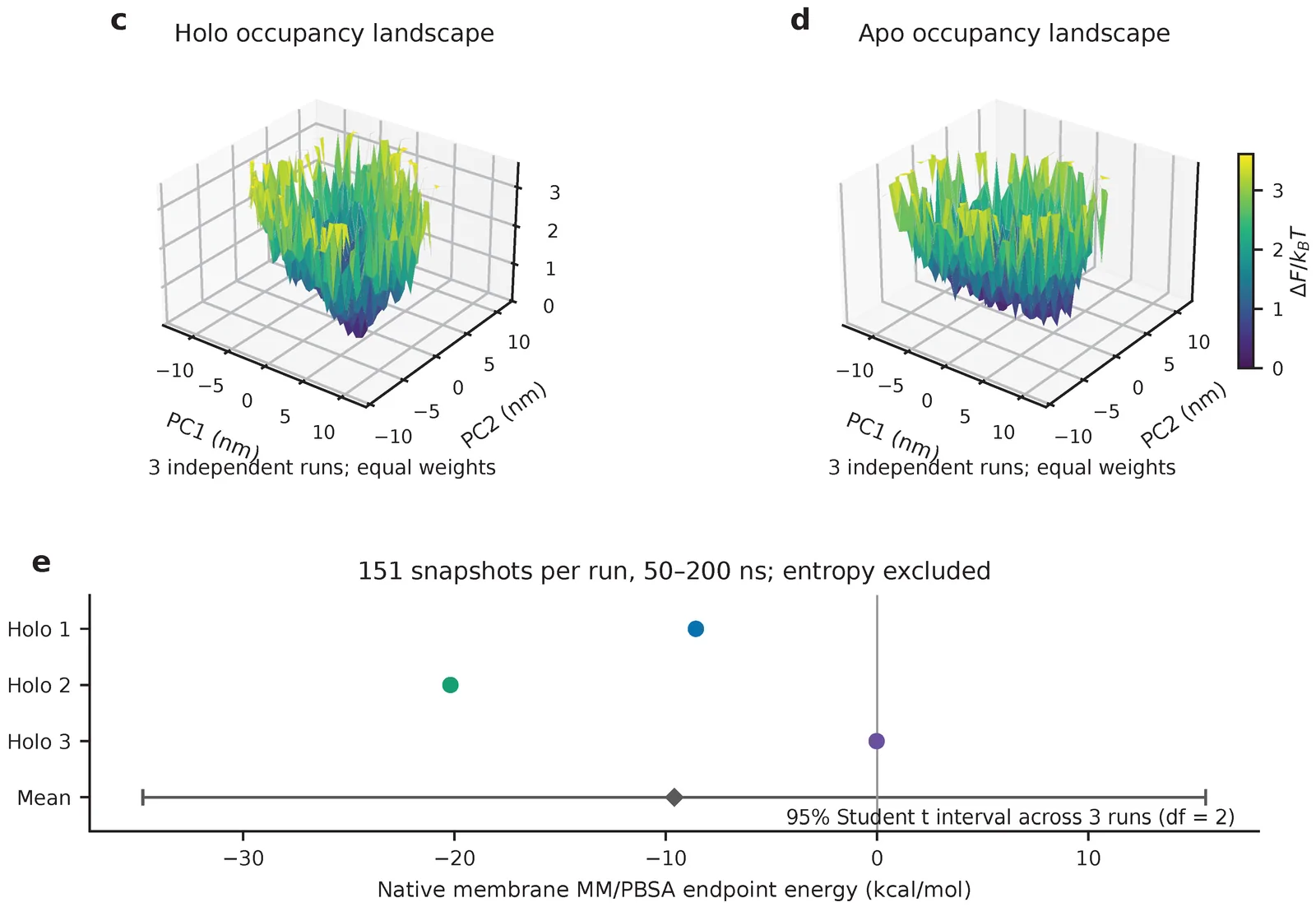
Replicated nirogacestat–γ-secretase simulations. (**a**,**b**) Pocket and ligand heavy-atom RMSD after PSEN1 fitting in three independent 200 ns runs per condition (ligand RMSD, holo only); dashed lines mark 50 ns. Ligand RMSD uses each run’s initial production frame. (**c**,**d**) Equal-run holo and apo occupancy landscapes on a shared Cα PCA basis; $\Delta F/k_{B}T=-\ln\left(p/p_{\max}\right)$ is normalised within each condition. Unvisited bins remain missing; colour and height scales are shared. (**e**) Membrane-MM/PBSA run means from 151 snapshots per holo run over 50–200 ns; the diamond and bar show the equal-run mean and its 95% confidence interval based on Student’s *t* distribution (df = 2). Entropy is excluded. Sampling uncertainty and the energy interval spanning zero limit interpretation; detailed analyses and display conventions are in Supplementary Methods S8, Table S3 and Figures S8–S23.

Across three independent 200 ns runs per condition, the mean pocket heavy-atom root-mean-square deviation (RMSD) after PSEN1 fitting over 50–200 ns was 0.216 nm in holo and 0.343 nm in apo models (holo-minus-apo difference, −0.128 nm; approximate Welch-*t* 95% interval, −0.178 to −0.077; Figure 7a). Ligand displacement and projected occupancy landscapes varied among runs and temporal blocks (Figure 7b–d), indicating substantial sampling uncertainty.

The mean membrane-MM/PBSA endpoint energy was −9.595 kcal/mol (descriptive three-run Student-*t* 95% interval, −34.739 to 15.549; Figure 7e). The interval spans zero, entropy was excluded and spacing sensitivity remained. These reference-dependent model results provide structural context for experimental testing; they do not establish binding affinity, potency or efficacy in SSc fibroblasts. Detailed structural, solvent-exposure, contact and energy results are reported in Supplementary Methods S8, Table S3 and Figures S8–S23.

## 4. Discussion

The clearest finding was a positive association between HES1 activity and model-derived myofibroblast fate across donors. In 206 donors, the median within-donor Spearman coefficient was 0.282 (95% CI, 0.258–0.309), and 98.1% of coefficients were positive. The direction persisted across cell-count thresholds and in graph reconstructions that excluded HES1 or its fixed regulon targets. This consistency supports a state-associated signal within the computational trajectory framework. Cross-sectional activity–fate correlations, however, cannot distinguish a causal regulator from a marker, a consequence of differentiation or a component under shared upstream control. The estimated fate probabilities are model outputs and do not directly observe cell transitions [18].

HES1 was a mechanism-guided focus within a broad regulator set. It ranked 28th of 107 and 30th of 109 in the complete CellOracle screens, while SMAD3 had a stronger within-donor fate association (median ρ=0.609). The screens therefore provide no basis for presenting HES1 as the leading unbiased regulator. Their algorithm-reported significance values also compare spatially autocorrelated grid positions with a single archived randomised field and are descriptive [6]. The rationale for examining HES1 is its role as a Notch-pathway readout and the availability of an upstream pharmacological intervention. Higher-ranked regulators remain relevant alternatives, and the current data do not demonstrate that HES1 has greater biological importance than SMAD3.

The disease contrast is a separate result. The adjusted SSc–HC HES1 activity difference was 0.21, with a confidence interval crossing zero and BH q=0.061. NOTCH1, NOTCH2, NOTCH3 and HES1 detection also showed no significant disease-associated increase. A state association shared by SSc and HC fibroblasts can identify a phenotype for experimental study, but it does not establish an SSc-selective target. Earlier experimental work implicated Notch signalling in SSc fibroblast activation and collagen release [3]; the present atlas does not independently demonstrate the same disease-specific activation. Similarly, the modest myofibroblast abundance difference depended on the multiplicity adjustment (BH q=0.036; Holm p=0.061). Its magnitude and uncertainty should be considered alongside the activity results.

The auxiliary analyses help define the scope of this association. SCENIC+ supplied candidate HES1 and RBPJ regulatory links, including links involving NRARP and HES5 [7]. The inferred network and the disease-accessibility analysis used the same GSE312129 source, and so they are complementary representations of that dataset. The archived eRegulon regions and the peaks eligible for disease testing are different sets: the focal scopes had fewer than the fixed ten overlapping tested regions and could not support a disease-level accessibility inference. The wider network size does not resolve that limitation. Held-out adult cohorts assessed the broader multiregulon panel, but removing HES1 changed the median AUROC only from 0.856 to 0.857. This performance does not establish an HES1-specific diagnostic contribution or predict drug response.

The spatial results provide a boundary on the proposed mechanism. All four donor-resolved LIANA+ SSc–HC contrasts were non-significant (BH q=0.714–0.812). The primary Visium DLL4–NOTCH2 comparison was also non-significant (p=0.0759; BH q=0.304), and all four Xenium mask-contact confidence intervals crossed zero (BH q=0.371–0.464). NicheNet rankings and representative contacts identify candidate ligand–receptor pairs for follow-up, but they do not demonstrate disease-specific signalling or enriched contact [24,25,26]. Visium and Xenium also use different spatial geometries and adjustment models; Xenium included only SSc sections and therefore supplies no SSc–HC comparison. A perivascular Notch route remains an experimental hypothesis.

The nirogacestat decision requires the same distinction between mechanism and efficacy. The candidate was selected by a retrospective mechanism-eligibility and translational-evidence assessment applied after the disease-expression query and L1000 compound scores had been frozen. Retaining the frozen query preserves the original expression comparison, but the added selection criteria introduce a post-ranking decision and potential selection bias. Nirogacestat ranked 6440 of 13,937 finite compounds, with a primary reversal score of −0.103. Its L1000 profile was discordant with the external tofacitinib-response signature from GSE209635 (alignment score −0.760) [28]. That trial evaluated a JAK inhibitor; accordingly, this comparison is an external transcriptomic annotation and cannot establish nirogacestat response or lack of response. RO4929097 had a stronger primary reversal score (−1.007; rank 3710), illustrating that the nirogacestat decision gave additional weight to clinical-development maturity. These findings provide weak and inconsistent expression-based support and do not identify nirogacestat as the strongest reversal candidate. The rationale for testing it rests on its upstream γ-secretase mechanism, prior SSc endothelial-cell observations and clinical-development maturity. The endothelial experiment addressed angiogenic dysfunction in a NOTCH4-risk context [5]; the fibroblast experiment involved a different fibrotic disease [4]. Neither establishes efficacy in SSc fibroblasts.

The experimental γ-secretase–nirogacestat pose supplies a starting point for structural modelling [30]. The simulations describe pocket behaviour under fixed membrane and protonation assumptions, with substantial sampling uncertainty and an endpoint-energy interval spanning zero. This structural context supports experimental follow-up; it does not establish affinity, potency or an SSc-fibroblast response.

Clinical-development maturity also brings safety constraints. The desmoid-tumour trial and prescribing information document gastrointestinal toxicity, ovarian toxicity, liver-enzyme and electrolyte abnormalities, and additional warnings [29,42]. These observations concern a different disease and treatment regimen. They warrant careful assessment of exposure, tolerability and viability in experimental work and cannot be assumed to define a suitable SSc regimen.

The immediate biological test would be a donor-replicated perturbation study in primary SSc and HC dermal fibroblasts. Matched vehicle conditions and exposure-dependent viability measurements would help interpret changes in cell state. NICD, HES1 and NRARP can assess pathway response, while α-smooth muscle actin, collagen and matrix production can assess fibrotic phenotypes. Parallel Notch-pathway and HES1 perturbations could help determine whether HES1 contributes to the observed state association. A contact-dependent co-culture experiment with JAG1- or DLL4-positive vascular cells could then address the spatial hypothesis. These experiments would test the candidate against the uncertainty and negative findings identified here.

## 5. Conclusions

Donor-resolved analyses associate HES1 activity with model-derived myofibroblast fate, while the disease-level activity contrast and candidate spatial Notch routes lack statistical support. Complete regulator rankings and shared-source multiome analyses position HES1 as a mechanism-guided research focus whose causal and SSc-specific roles remain unresolved. Nirogacestat is a candidate compound for testing upstream γ-secretase inhibition, selected through a retrospective mechanism and translational-evidence assessment despite weak expression-reversal and discordant external treatment-response alignment. The resulting hypothesis is that Notch-pathway perturbation may alter pro-fibrotic states in SSc fibroblasts. Donor-replicated experiments with pathway, cell-state and viability readouts are needed to establish whether that hypothesis is supported.

## Figures and Tables

**Table 1:** Main findings and inferential scope. Rows estimate different quantities and are not one validation chain.

Question	Independent Unit	Result	Interpretation
Myofibroblast abundance	230 donors; cohort-adjusted	OR 1.31 (1.03–1.67); BH q=0.036, Holm p=0.061	Modest, correction-sensitive difference
HES1 disease activity	220 donors (141 SSc, 79 HC)	Difference 0.21 (−0.01–0.43); BH q=0.061	Uncertain disease contrast
HES1 activity–fate	206 donors (132 SSc, 74 HC)	Median ρ=0.282 (0.258–0.309); 98.1% positive	Model-derived state association
CellOracle ranking	Fixed model/grid; two priors	HES1 28/107 and 30/109	Mechanism-guided focus within a wider regulator set
Candidate Notch routes	Donors/sections, by assay	Disease and contact tests non-significant	Exploratory routes for experimental testing

**Table 2:** Expression evidence and the retrospective decision. Scores are transcriptomic annotations, not affinity or clinical potency. Lower primary scores indicate stronger reversal; the rank denominator is 13,937 finite compounds.

Compound	Primary Score	Rank	Interpretation
Nirogacestat/PF-03084014	−0.103	6440	Weak reversal and discordant tofacitinib-response alignment (−0.760). Selected post-ranking on mechanism and maturity.
RO4929097	−1.007	3710	Stronger primary reversal; an alternative requiring direct comparative testing.

## Data Availability

RNA and multiome data are available from GEO under the retained accessions listed in the Materials and Methods and supplement. Xenium data are from GSE312932; the Visium collection is deposited at https://doi.org/10.5281/zenodo.14577696. PDB 8KCT supplies the experimental structure. Public analysis code is available at https://github.com/fsy2004/ssc-virtual-perturbation (accessed on 12 October 2026) [59]. The checksum-verified MD revision archive at https://github.com/fsy2004/ssc-virtual-perturbation/releases/tag/revision-md-20261008 (accessed on 12 October 2026) contains executed analysis code and result tables, both processed holo/apo model topologies, representative native production inputs and the six-run source manifest. Complete native whole-system trajectories, energy files, checkpoints and per-run input dependencies are available from the corresponding author upon reasonable request. The coordinate trajectories alone total approximately 161.83 GB; bulk-file transfer and storage constraints require separate access arrangements for these native files. Public numerical summaries are accompanied by their source identities and are not substitutes for the original trajectories.

## Supplementary Materials

The supporting information can be downloaded at https://www.mdpi.com/article/10.3390/biomedicines14102360/s1. Supplementary_Materials.pdf contains the following methods, tables and figures. Supplementary Methods: S1. Composition sensitivities; S2. CellOracle reconstruction; S3. Activity and trajectory sensitivities; S4. Adult held-out-cohort validation; S5. SCENIC+ and accessibility; S6. Spatial models; S7. Communication and mask-contact tests; S8. Molecular dynamics and membrane MM/PBSA. Table S1. Software version evidence. Table S2. O6U analogy penalties of at least 10. Table S3. Membrane-MM/PBSA independent-run and temporal-block means. Energies are in kcal/mol and exclude entropy. The three runs are independent; snapshots and within-run blocks are correlated. The last column is the trapezoidal time-weighted 1-minus-5-ns difference. Figure S1. Donor-balanced marker evidence. Inflammatory-like is descriptive. Figure S2. Selected HES1/RBPJ triplets, association signs and within-run ranks. Figure S3. Representative expression-positive shared-mask-edge contact; an example does not establish enrichment. Figure S4. Donor-balanced descriptive mean ULM activities for HES1, SMAD3 and MEF2C across seven fibroblast states. Cell activities are averaged within donor/state before equally weighting donors. State-specific donor counts differ; Inflammatory-like has one HC donor and is descriptive. The 3999 unassigned fibroblasts lack saved activity and are omitted. These summaries are not disease contrasts. Figure S5. Held-out discrimination/calibration for HES1-only, full-panel and HES1-removed models. Figure S6. Auxiliary route display, NicheNet ranks and Xenium transport localisation. Figure S7. Donor fibroblast-state composition with all retained skin cells as denominator. Figure S8. Six-run common-basis principal-component trajectories. Independent runs share one matched-atom mapping and covariance basis. Figure S9. Six-run projected occupancy landscapes. Unsupported histogram bins remain missing; each surface has its own additive zero. Figure S10. Three-dimensional holo and apo projected occupancy landscapes. These are conformational occupancy surfaces, with finite sampling and no affinity or kinetic-barrier inference. Figure S11. Independent-run and temporal-block occupancy support. Four 50-ns blocks describe within-run sampling; three runs per condition define comparison units. Figure S12. Sampling and histogram-resolution diagnostics. Autocorrelation and occupancy differences qualify interpretation rather than certify convergence. Figure S13. Protein solvent-accessible surface area for each of six independent runs. Figure S14. Holo ligand geometric exposure and interface areas, with explicit source selections. Figure S15. Independent-run and 50-ns-block SASA summaries. Three-run bootstrap intervals are descriptive. Figure S16. SASA native-stride and point-resolution sensitivity. Numerical controls retain the source geometric definitions. Figure S17. Membrane-MM/PBSA endpoint estimates from three independent runs, each sampled at 151 times over 50–200 ns. (a) Per-run energy traces. (b) Run means and the equal-run mean with a Student-*t* 95% interval (two degrees of freedom). (c) Trapezoidal time-weighted 1-minus-5-ns differences; dashed lines mark the predeclared 0.5-kcal/mol spacing diagnostic. Entropy is excluded and the between-run interval includes zero. Figure S18. Supported amino-acid endpoint components from all 453 snapshots. Points and bars show equal-run means and descriptive three-run Student-*t* 95% intervals. The 16 largest absolute means are displayed for exploration; all 1313 protein residues and complete temporal-block summaries are retained in the source tables. Global nonpolar energy is separate, so the plotted sums are not full per-residue TOTAL energies or experimentally established hotspots. Figure S19. Structural dynamics of six independent runs. Traces display 100-ps observations from metrics calculated every 10 ps. Each run uses its own EQ6 reference; atom selections and fitting definitions are matched between conditions (Methods S8). Protein $R_{g}$ uses the production TPR masses after hydrogen mass repartitioning. The vertical line at 50 ns marks the reporting window. Figure S20. Run-level structural summaries over 50–200 ns. Circles represent the three independent run means per condition. Squares and bars show the equal-run mean and descriptive Student-*t* 95% interval with two degrees of freedom. Holo and apo runs are unpaired. Full 0–200-ns summaries and unpaired Welch intervals are provided in the source tables. Figure S21. Residue flexibility over 200 ns. Cα RMSF is calculated separately for each run after unweighted PSEN1 fitting. Faint lines show individual runs, solid lines show equal-run means, and shading shows between-run standard deviations. Grey marks identify the predefined pocket. Only observed model residues are plotted. Source tables distinguish whole-trajectory RMSF from mean within-50-ns-block RMSF over 50–200 ns. Figure S22. Ligand displacement, exposure and hydrogen-bond interactions. Ligand RMSD uses protein fitting without ligand self-alignment. Ligand SASA uses the membrane/glycan/ligand environment, RDKit van der Waals radii, a 1.4-Å probe and 256 deterministic surface points; 1024-point resolution controls are retained separately. Direct hydrogen bonds require donor–acceptor distance ≤3.5 Å and donor–H–acceptor angle $\geq 150^{\circ }$. A bridged residue requires two qualifying bonds through the same water oxygen in the same frame. Bond and bridged-residue counts have different units. Figure S23. Residue-level ligand-contact occupancies. Source tables retain all 1313 modelled protein residues and three event types. Each panel displays up to 25 residues with maximum occupancy ≥10% in any run, ordered by that maximum. This is a descriptive display selection. Heavy-atom contacts require distance ≤4.5 Å. Occupancy is the fraction of native 10-ps frames with at least one qualifying event for the residue. Water bridges retain the same-frame, same-water definition in Methods S8. Figure S24. Donor communication scores and spatial disease contrasts. (a) LIANA+ magnitude scores for HC (blue) and SSc (orange): 133 eligible donors for JAG1 and 112 for DLL4 routes. Cohort-adjusted disease intervals include zero (BH q=0.714–0.812). (b) Visium COMMOT SSc–HC differences and 95% intervals in 14 donor-labelled sections (10 SSc, 4 HC); disease tests use all 1001 condition-label assignments. BH adjustment covers four routes. Raw donor scores and adjusted disease differences have distinct scales. Xenium contact enrichment and receiver detection are shown separately in main Figure 6; Methods S7 specifies the settings and null assumptions. Figure S25. Fixed-model transition-field reconstruction and donor summaries. Left to right: reference transition field, HES1 knockout-weighted field, their exact difference, and fixed-field donor summaries for SMAD3, MEF2C and HES1. The reference and knockout fields use the archived 29,985-cell object and the same 40-by-40 display grid. The difference reproduces the exported calculation (maximum numerical discrepancy $1.55\times 10^{-15}$). Donor points summarise the fixed mature-Myofibroblast field; diamonds and bars show medians and 10,000-donor-bootstrap 95% intervals. The HES1 summary includes 200 donors. These descriptive estimates condition on the fitted network and do not measure donor-specific network-refitting uncertainty, observed differentiation or causal effects. Methods S2 provides the developmental-reference and lineage definitions. The supplementary methods and software documentation cite the following references: [6,7,8,9,13,14,15,16,17,18,19,20,21,22,23,24,25,26,27,30,31,32,33,34,35,36,37,38,39,40,43,44,45,46,47,48,49,50,51,52,53,54,55,56,57,58].

## Abbreviations

AUCell, area under the recovery curve; AUROC, area under the receiver operating characteristic curve; BH, Benjamini–Hochberg; CI, confidence interval; DAR, differential accessibility region; eGRN, enhancer-driven gene-regulatory network; eRegulon, enhancer-driven regulon; FDR, false-discovery rate; GRN, gene-regulatory network; HC, healthy control; HMR, hydrogen mass repartitioning; KO, knockout; MD, molecular dynamics; MM/PBSA, molecular mechanics/Poisson–Boltzmann surface area; OR, odds ratio; PBMC, peripheral blood mononuclear cell; PDB, Protein Data Bank; POPC, 1-palmitoyl-2-oleoyl-sn-glycero-3-phosphocholine; PSEN1, presenilin 1; RCTD, robust cell-type decomposition; RMSD, root mean square deviation; scATAC, single-cell assay for transposase-accessible chromatin; SSc, systemic sclerosis; TF, transcription factor; ULM, univariate linear model; UMAP, uniform manifold approximation and projection.
