# Supplementary material for: Donor-Resolved Analysis of HES1-Associated Fibroblast States in Systemic Sclerosis
Source: Biomedicines. 2026 Oct 16;14(10):2360. doi: 10.3390/biomedicines14102360 (PMC13702352; doi:10.3390/biomedicines14102360)
Supplement: Supplementary file 1 [file biomedicines-14-02360-s001.zip › biomedicines-4589813-supplementary.pdf]

Supplementary Materials
Donor-resolved analysis of HES1-associated fibroblast states in systemic sclerosis

Fang et al.

15 October 2026

Contents

S1. Composition sensitivities **2**

S2. CellOracle reconstruction **2**

S3. Activity and trajectory sensitivities **2**

S4. Adult held-out-cohort validation **3**

S5. SCENIC+ and accessibility **3**

S6. Spatial models **4**

S7. Communication and mask-contact tests **4**

S8. Molecular dynamics and membrane MM/PBSA **5**

Supplementary figures **11**

S1. Composition sensitivities

Cell-type and fibroblast-state counts were summed across technical libraries from the same donor after tissue filtering and duplicate removal. The deposited donor or patient identifier was the independent unit. For total fibroblasts and each fibroblast state, the primary denominator was all retained SSc or HC skin cells from that donor. Each endpoint used a beta-binomial logit model in `glmmTMB` [1]: `cbind(count, total-count) ~ condition + cohort`. We extracted SSc-versus-HC odds ratios, Wald 95% confidence intervals and raw p values. Eight endpoints were planned; Benjamini–Hochberg and Holm adjusted the seven estimable tests, with Inflammatory-like descriptive.

Supporting analyses covered the 14 mutually exclusive major cell types with the all-skin-cell denominator, the seven states divided by assigned fibroblasts, and leave-one-cohort-out refits. For composition-aware sensitivity, sccomp [2] fitted sum-constrained beta-binomial models with condition and cohort effects. Separate models covered the 14 major types and the seven fibroblast states plus all other skin cells. The effect threshold was zero, the false-positive target was 5%, and the seed was 20260723. The 54-cell Inflammatory-like state occurred in one HC donor and was not treated as an estimable endpoint.

S2. CellOracle reconstruction

CellOracle [3] was used to model TF knockout on the fibroblast manifold. A skin-specific base GRN was constructed from GSE312129 snMultiome chromatin accessibility using Cicero co-accessibility, TSS links and TF motif enrichment [4]; a promoter-based GRN provided a second prior. Every TF with non-zero fitted regulatory edges in each prior was evaluated. For each TF, expression was fixed at zero, the perturbation was propagated for three steps, and the transition field was estimated with 200 neighbours, full cell sampling, random seed 0 and `sigma_corr`=0.05. A SFRP2/DPP4-rooted diffusion-pseudotime flow, oriented towards a fixed myofibroblast marker score, provided the developmental reference.

We reproduced the perturbation score directly from the archived engine. At retained Myofibroblast grid positions, KO-shift and developmental-reference vectors were paired by location. The saved signed score is the negative sum of negative inner products. CellOracle's `estimate_transition_prob(calculate_randomized=True)` generates a randomised-GRN control field. The function `get_negative_PS_p_value` applies a paired one-sided Wilcoxon comparison of observed and randomised-control negative inner products across retained positions. BH adjustment was applied across the complete prior-specific candidate space, and complete ranks and algorithm-reported statistics were exported before candidate interpretation. Only one randomised field was archived for each TF/prior run, so there is no replicated domain-level null; in addition, neighbouring grid positions are spatially autocorrelated. We therefore treat the reported p and BH q values solely as descriptive grid-separation indices, not as formal inferential statistics or a basis for TF selection.

The complete edge-active screens used the same engine within each prior. All attempted TF simulations succeeded and were ranked within their respective prior-specific tables. The promoter prior had 109 attempted and 109 ranked TFs. The skin-scATAC prior had 107 attempted and 107 ranked TFs. Neither analysis had a failed simulation.

The archived 3.54-GB Oracle object was reopened in CellOracle 0.20.0 and analysed at its full 29,985-cell size for HES1, SMAD3 and MEF2C. For each factor, the uniform local-neighbour reference and correlation-weighted KO transition were exported on the same 40-by-40 grid. Their explicit difference reproduced `delta_embedding` within 10^{-8}. Cell-level field summaries were joined to donor identifiers after duplicate removal. Donor medians, cohort summaries, leave-one-cohort-out estimates and donor-count thresholds were calculated in the retained developmental-gradient mass region. Fixed-field within-donor downsampling used fractions 0.50, 0.75 and 1.00 with 100 repeats. These summaries did not refit the GRN within donors or cohorts. A separate lineage analysis compared the existing SFRP2/DPP4, SFRP4-positive pro-fibrotic and Myofibroblast labels. The labels followed the established literature annotation. Inference used 10,000 donor-bootstrap replicates and BH/Holm adjustment within each three-state family. Pseudotime windows, labels, neighbour counts and thresholds were held fixed in these summaries.

S3. Activity and trajectory sensitivities

To obtain a measure of TF activity complementary to the perturbation engine, we inferred per-cell activities using decoupler's univariate linear model (ULM) [5] with the CollecTRI regulon collection [6]. Cell identifiers were mapped to reconstructed deposited donors after exclusion of non-skin and duplicated reprocessing. Within each donor, activity was aggregated across Myofibroblast cells. SSc–HC contrasts were fitted with `activity ~ condition + cohort`

OLS and HC3 robust standard errors. Adjusted mean differences, 95% CIs, standardised effects, BH and Holm values and leave-one-cohort-out refits were reported for HES1, SMAD3 and MEF2C. The Inflammatory-like state was descriptive because it contained 54 cells from one HC donor.

Archived CellRank2 myofibroblast fate probabilities were joined to CollecTRI/ULM activities by exact cell identifier [7]. Technical libraries were merged within deposited donors. The primary analysis required at least 30 fibroblasts per donor; thresholds of 20 and the outcome-blind donor-count quartiles 63, 140 and 405 provided sensitivities. Within each donor, Spearman ρ was calculated between TF activity and myofibroblast fate probability. Median coefficients received 10,000-donor-bootstrap 95% CIs; signed-rank tests, sign tests, BH and Holm adjustment covered HES1, SMAD3 and MEF2C. Cohort summaries and leave-one-cohort-out results were exported.

For leave-feature-out sensitivity, we reconstructed full-gene `.raw` matrices from six cohort H5AD files after donor and duplicate filtering. The non-redundant matrix contained 89,045 fibroblasts. One outcome-blind 2,000-HVG set was selected before three arms were defined: baseline, removal of HES1, and removal of 76 fixed CollecTRI HES1 targets. Cells, donor mapping, archived activity values, root cell, terminal-state rule and graph parameters were fixed. Each arm independently rebuilt Harmony by cohort, a 30-neighbour graph, diffusion pseudotime and a hard-thresholded CellRank PseudotimeKernel. Connected graphs, finite normalised fate probabilities and hashed inputs and outputs were required. A complete one-job PETSc rerun checked numerical stability. This sensitivity evaluates graph-feature leakage and solver stability. It cannot establish causality.

S4. Adult held-out-cohort validation

Eight adult whole-skin cohorts formed the primary nested held-out-cohort validation: GSE130955, GSE58095, GSE249550, GSE181549, GSE9285, GSE32413, GSE76807 and GSE231692. GSE95065, GSE125362 and baseline GSE76885 were added to a sensitivity analysis because of sparse publication or batch metadata, small size or longitudinal sampling. GEO family SOFT files and linked publications defined disease, tissue, baseline and participant eligibility. Repeated samples or biopsy sites were aggregated within participant after baseline selection. GSE130955 contained 91 biopsies from 81 participants. Normalisation of zero-padded GSE58095 identifiers revealed 61 SSc and 36 HC participants; four later SSc biopsies were excluded and two same-time biopsies from one participant were averaged. GSE249550 contained 93 unique baseline participants. The five added primary cohorts contributed 283 participants, and the three sensitivity cohorts contributed 91.

Each outer fold held out one complete cohort. Median imputation, standardisation, L2 logistic regression [8] and selection of the regularisation parameter C were confined to training cohorts. Inner leave-one-training-cohort-out AUROC selected C, with equal weight for each cohort. We compared three fixed feature sets: HES1 alone, all 668 shared CollecTRI/ULM activities, and the 667-feature panel without HES1. The 11-cohort sensitivity shared 652 activities. Ten thousand participant-stratified bootstrap resamples of each test cohort provided intervals for AUROC, average precision and Brier score. They also provided paired differences between feature sets. We reported SSc prevalence, calibration intercept, calibration slope and prevalence-binned calibration curves. The legacy and live decoupler 2.1.6 CollecTRI networks contained the same 42,990 signed edges.

S5. SCENIC+ and accessibility

SCENIC+ [9] was run on the GSE312129 skin single-nucleus multiome source that also defined the CellOracle skin regulatory prior. Chromatin-accessibility topics were inferred with pycisTopic [9] and MALLET [10]. Candidate regions were linked to motif enrichment, and the SCENIC+ Snakemake workflow inferred enhancer-driven regulons [11]. We exported all 266 TF summaries, 502 direct eRegulons, 285 extended eRegulons and 240,864 TF–region–gene triplets. Each triplet retains its direct or extended membership and the signs of the inferred TF-to-gene and region-to-gene associations. Raw importance and ranking values were converted to within-run percentiles. Supplementary Figure S2 used the product of TF-to-gene importance, region-to-gene importance and triplet-rank percentiles to order the 317 HES1/RBPJ triplets. For each TF, the display retained six direct and two extended triplets without repeating a gene or region.

The three direct HES1 eRegulons were expanded to gene-level membership tables, and their pairwise overlaps were calculated. Archived AUCell values were summarised by cell type. The retained AUCell table lacks donor and condition fields, so donor-level SSc–HC inference used the separate CollecTRI analysis. SCENIC+ supplied enhancer-network context from the shared GSE312129 source.

For the condition-aware accessibility analysis, all fibroblast barcodes were retained for each of four HC and ten SSc GSE312129 donors. Fragments were filtered in a streaming pass without cell sampling. Regions entered the

donor-pseudobulk matrix when they belonged to the condition-union peak set and were accessible in at least two fibroblast donors within either condition. The condition union contained 421,185 regions; donor support retained 421,173 regions across 9,087 fibroblasts. edgeR quasi-likelihood models [12] with design ~ `condition` tested 69,152 peaks that passed expression filtering.

Archived direct and extended eRegulon regions were intersected with the disease-tested peaks by exact canonical genomic coordinates. This intersection includes only peaks meeting the condition-union, donor-support and edgeR eligibility rules described above. Archived network membership alone does not imply inclusion in the disease-tested subset. A TF-scope test required at least ten overlapping regions. Its statistic was the median SSc-versus-HC peak logFC. The matched null used 9,999 bootstrap region draws with replacement, stratified by logCPM and region-length deciles. Of 336 evaluated TF scopes, 118 met the ten-region gate. BH adjustment covered the eligible scopes with finite empirical p values. HES1, RBPJ and SMAD3 were designated as focal TFs, but their focal scopes did not meet the minimum-region gate. The eRegulons and donor DAR derive from the same GSE312129 source.

S6. Spatial models

Visium and Xenium were analysed separately because their resolution, gene coverage and estimands differ. The Visium collection contained 14 donor-labelled sections (10 SSc and 4 HC), one per donor. Its primary graph used native 10x first-ring hexagonal adjacency. We compared the six-gene myofibroblast programme with a HES1 CollecTRI/ULM score after removing the overlapping targets TAGLN, COL1A1 and COL1A2. Within each section, we residualised sequencing depth, RCTD cell-composition CLR principal components explaining 90% of variance, and BANKSY domain [13]. Bivariate Moran's I was computed on the fixed graph [14]. Residual labels were permuted 5,000 times within outcome-blind coordinate blocks nested in BANKSY domains. Benjamini–Hochberg and Holm procedures covered the 14 sections. Exact binomial intervals quantified Monte Carlo uncertainty. Sensitivities varied block size, graph definition and composition method and included leave-one-donor-out summaries.

The Xenium set contained ten SSc donor-labelled sections, one per donor. Because the targeted panel lacks COL1A2 and FN1, a separate four-gene programme was evaluated within high-confidence RCTD fibroblasts on a symmetric 6-nearest-neighbour graph. Depth, fibroblast state and macro-region were adjusted before Moran analysis. Fixed-graph residual labels were permuted 5,000 times within study-specific coordinate blocks nested in macro-regions; BH and Holm corrections covered the ten sections. Annotation thresholds, block sizes and adjustment sets were varied as sensitivities. Ten-thousand-donor bootstrap intervals and leave-one-donor-out means were reported for both platforms. The block-restricted permutation preserves regional marginals; fine-scale autocorrelation remains outside the exchangeability assumption of this study-specific null.

S7. Communication and mask-contact tests

Ligand-receptor signalling was analysed with NicheNet, donor-resolved LIANA+ and COMMOT. The NicheNet candidate universe required expression in a fixed sender population, an edge to a receptor expressed in myofibroblasts, representation in the NicheNet v2 ligand-target matrix and a bona fide ligand annotation. For the final criterion, we queried the OmniPath composite intercell annotation for generic functional protein ligands [15]. Candidates required a consensus score of at least four and annotation as secreted, plasma-membrane transmembrane or plasma-membrane peripheral. The inclusion and exclusion table was exported before ranking.

NicheNet [16] ranked the resulting 100 ligands by AUPR-corrected activity against the myofibroblast transcriptional programme. It also calculated predicted ligand-to-target potentials for the Notch ligands, with TGFB2 as a positive control. In the default single-ligand mode, filtering changed each retained ligand's rank within the audited universe but not its activity score.

LIANA+ [17] was run separately within each deposited donor using the consensus resource, an expression-proportion threshold of 0.10 and at least ten sender and receiver cells. Technical skin libraries were merged before inference. The four primary routes paired pericyte JAG1 or endothelial DLL4 with myofibroblast NOTCH2 or NOTCH3. The primary donor score was $-\log_{10}$ of the aggregate magnitude rank. Eligible donors with no returned interaction received zero. Each score was regressed on condition and cohort with HC3 confidence intervals. Primary p values came from 9,999 condition-label permutations within cohort, followed by BH and Holm correction across the four routes.

COMMOT [18] modelled each route independently on the observed spatial geometry. All spatial units remained in the graph. Fixed RCTD weights defined JAG1 senders as endothelial plus pericyte, DLL4 senders as endothelial, and receivers as pro-fibrotic fibroblasts. For each route, the sample score was $\log(1 + \sum C_{ij} w_i w_j / n)$, where C is

the COMMOT transport matrix, w_i and w_j are sender and receiver weights, and n is the number of spatial units. The 14 Visium samples were compared with all 1,001 assignments of four HC labels using the fixed two-sided test, with BH correction across the four routes. Xenium COMMOT retained sections with at least 50% RCTD coverage and measured within-SSc localisation.

Receiver-gene detection was audited after reconstructing deposited participant identifiers from GEO and sample metadata. The analysis retained the five non-redundant skin cohorts and merged technical or sorted skin libraries within donor. Positivity for NOTCH1, NOTCH2, NOTCH3 and HES1 was defined as expression greater than zero in Myofibroblast cells. The primary analysis included all 220 donors with at least one Myofibroblast (141 SSc and 79 HC). Positive and total counts were summed within donor and modelled as `cbind(positive, total-positive) ~ condition + cohort` with a beta-binomial logit link in `glmmTMB`. Wald 95% CIs were reported, and BH and Holm procedures adjusted the four genes. Higher cell-count thresholds and leave-one-cohort-out refits remain in the archived workflow.

Direct adjacency was tested in the ten GSE312932 SSc Xenium sections using the deposited `cells.zarr.zip` segmentation labels in `/masks/1`. Mask labels were mapped one-to-one to H5AD cells by transformed centroids. RCTD labels [19] were retained when the maximum cell-type weight was at least 0.5. JAG1 senders were JAG1-positive endothelial cells or pericytes. DLL4 senders were DLL4-positive endothelial cells. Receivers were pro-fibrotic fibroblasts positive for NOTCH2 or NOTCH3, with positivity defined by at least one detected Xenium transcript. Two cells were directly adjacent when their segmentation masks shared a pixel edge. The statistic was the number of shared edges connecting an expression-positive sender and receiver.

The graph, cell types and positive-label counts were fixed. Positive labels were permuted 5,000 times within cell type and 500-μm coordinate blocks in each section. This study-specific restriction preserves coarse stratum marginals. Fine-scale autocorrelation remains outside its exchangeability assumption. One-sided plus-one permutation p values compared pooled observed and null edge counts. Exact Clopper–Pearson intervals quantified Monte Carlo uncertainty. Benjamini–Hochberg, Holm and single-step studentized maxT procedures assessed the four focal pairs. Pooled $\log_2$(observed/expected) effects received 95% CIs from 10,000 section-level bootstrap resamples. Leave-one-section-out effects were also exported. Sensitivities varied contact definition, mask gap, graph, annotation, block size, block origin and permutation stratum.

Plotting and revision provenance

Submitted statistical plots and layouts used Matplotlib 3.11.0, as recorded in PDF metadata [20]. Revised plots used Python 3.12.5 and Matplotlib 3.11.0, with NumPy and pandas for arrays and source tables [21, 22]. PyMuPDF and pypdf handled preserved PDF layers and replacement labels [23, 24]. The unchanged composition model was refitted in R 4.4.3 with glmmTMB 1.1.14 [1, 25]; donor activity/fate summaries were reproduced with statsmodels for OLS/HC3 and multiplicity adjustment, and SciPy for correlations and rank/sign tests [26, 27]. Figures use recovered source tables where available; vector-preserved panels are identified separately in the provenance record. Tables are editable LaTeX. Supplementary Table S1 documents the workflow versions and their provenance.

S8. Molecular dynamics and membrane MM/PBSA

Structural starting model and preparation

PDB 8KCT was selected because it directly resolves nirogacestat (O6U) bound to human γ-secretase at 2.60 Å [28]. The historical 6LR4 reference contains semagacestat, so it supplies neither an experimentally observed nirogacestat pose nor an apo reference [29]. This is a ligand-identity comparison, not a quantitative ranking of alternative structures. The 8KCT validation report contains O6U bond/angle outliers and a short Leu432–O6U contact. Local preparation checks evaluated ligand stereochemistry and contacts; these geometric checks make no separate ligand-density-fit claim.

CHARMM36m was selected for its refined protein-backbone treatment and compatibility with the CHARMM additive lipid, carbohydrate and ligand models [30–32]. The membrane uses CHARMM36-compatible lipids and carbohydrates, and modified CHARMM TIP3P water. Force-field compatibility is assessed separately from accuracy for this complex and catalytic-site protonation.

O6U parameters originate from automated CGenFF analogy assignment through CHARMM-GUI Ligand Reader [33], using a CCD-named MOL with explicit hydrogens and `guess_bond_order=false`. The source specifies CGenFF program 4.0 and topology/parameter release 5.0. All 76 current production atom names, types and charges map to the initial stream; the 78-bond graph and custom angle/dihedral coefficient conversions were checked against

the processed production topology. Maximum bonded and charge penalties are 35.5 and 32.824. Table S2 lists every term with penalty at least 10; none reaches 50. These values describe analogy confidence and do not establish parameter accuracy.

Completed source-bound ligand checks comprise the actual production-potential MM minimum, C09(S)/C14(S) stereochemistry, a fixed-conformer HF/6-31G(d) single-point calculation and 32 rigid water-interaction calculations in four selected directions at eight distances. Classical-charge and HF dipole magnitudes on the same fixed conformer were 4.843 and 5.899 D. Individual rigid-grid values, repulsive selected carbonyl geometry and grid-boundary minima are retained. The validation scope is a fixed-conformer single point and a rigid water grid, without relaxed geometry calibration, BSSE correction or parameter refitting. These limited diagnostics do not establish full parameter accuracy; no parameter or production trajectory was changed by these checks.

CHARMM-GUI Membrane Builder generated construction inputs and GROMACS templates [34–36]. Its internal CHARMM 47a2 preparation included 100 steepest-descent steps [37]. Subsequent assembly preserved protected coordinates, corrected newly added POPC packing and placed reviewed local cavity waters. Waters at introduced hard overlaps were handled as whole molecules; no blanket membrane-wide or protein-cavity water deletion was applied. GROMACS performed the research minimisation and equilibration separately from these construction operations.

Both systems contain 571 added POPC molecules, three native sterols and two native phosphatidylcholines. Holo contains 358,780 atoms, including 76 O6U atoms and 86,607 waters. Apo contains 358,758 atoms and 86,625 waters after removing O6U and adding 18 local waters. The 358,704 shared atoms retain matched starting coordinates. Terminal patches, protein fragments, disulfides, glycosylation, box and ions are matched. Asp257 is deprotonated and Asp385 carries an OD2 proton in both models; O6U is neutral. These are stated fixed microstates, and the ligand-deleted model is not an experimentally determined apo ensemble.

The protein/glycan component has charge $-3e$, balanced by 241 Na^+ and 238 Cl^-: 238 salt pairs and three counterions. Lipids, waters and the selected O6U microstate are neutral. Solute partial charges were not edited to force neutrality. Static source and topology checks covered atom order, chemical connectivity, patches, molecule counts, box periodicity and initial water/lipid placement. Membrane integrity was assessed from the trajectories as described below.

Simulation protocol and quality review

Nonwater hydrogen masses were repartitioned from 1.008 to 3.024 u, with 2.016 u transferred from each bonded heavy atom per changed hydrogen. Every molecular mass, atom type, charge and bond is conserved; water and ions retain ordinary masses. Compiled TPR masses and charges were checked; `mass-repartition-factor=1` prevents a second repartitioning. The production timestep is 2 fs with LINCS order 4/one iteration for hydrogen-involving bonds and SETTLE water.

GROMACS 2026.3 in mixed precision with CUDA performs minimisation, equilibration and production [38]. EQ1–6 consists of two 125-ps NVT stages at 1 fs, one 125-ps semi-isotropic NPT stage at 1 fs and three 500-ps NPT stages at 2 fs with progressive restraint release (1.875 ns total). Each repeat generates independent initial velocities and follows its own equilibration checkpoints; this samples velocity/equilibration realisations of one membrane scaffold. Production uses each run's own initial equilibrated state and then its full production checkpoint for continuation, without regenerated velocities.

The target temperature is 310.15 K with V-rescale SOLU/MEMB/SOLV groups and $\tau_T = 1$ ps. Semi-isotropic C-rescale targets 1 bar with $\tau_P = 5$ ps. Both coupling execution periods are 0.2 ps. PME starts with a 1.2-nm real-space cutoff, CHARMM force switching spans 1.0–1.2 nm, and dispersion correction is disabled. Actual runtime neighbour-list and PME tuning is retained in the logs. Whole-System coordinates are saved every 10 ps, energies every 2 ps and checkpoints every five minutes. Continuous extensions change only TPR step count and retain production time, velocities and coupling state.

Each of the three holo and three apo replicas completed its own 200-ns production trajectory and major numerical/structural review. Initial EQ1 velocity seeds, EQ1–6 checkpoints and production sources are independently retained; no production checkpoint was copied between replicas. The total production dataset is 1,200 ns. The adopted design has three independently equilibrated 200-ns runs per condition. Whole-System native trajectories, checkpoints, energies, inputs and logs are retained with checksums and available from the corresponding author upon reasonable request. Executed code, model inputs and analysis results are linked in the main Data Availability statement.

Long-window review examined numerical and group-temperature behaviour, boxes and periodic images, local leaflet/tail structure and periodic seams, water identities and paths, protein/pocket/ligand geometry and sampling. Normal γ-secretase cavity hydration is distinguished from a water pathway through a bilayer defect. Shared boundaries

and off-grid native observations retain their original values and sources. No single water count, absence of LINCS warnings or short-window mean temperature establishes acceptance.

Structural, sampling and endpoint-energy analyses

Structural metrics were calculated at native 10-ps times with matched protein and pocket atom selections in all six runs. RMSD used each run's equilibrated reference and either PSEN1 Cα or whole-complex fitting; ligand RMSD used protein fitting without ligand self-alignment. R_g retained the production TPR hydrogen-repartitioned masses, with heavy-atom geometric R_g reported separately. Direct hydrogen bonds required donor–acceptor distance ≤ 3.5 Å and donor–H–acceptor angle $\geq 150°$. A water-bridged residue required two qualifying bonds through the same water oxygen in the same frame. Whole-trajectory RMSF and mean within-50-ns-block RMSF over 50–200 ns were kept as distinct estimands. Condition comparisons used independent run means, with unpaired Welch-t intervals for holo-minus-apo differences; individual-condition intervals used Student-t with two degrees of freedom. These descriptive intervals have only three independent runs per condition. Display traces use native 100-ps subsampling. Supplementary Figures S19–S23 retain each run separately.

The completed six-run common PCA/FEL uses 1,313 matched Cα atoms, 2,001 observations per run at 100-ps spacing, equal run contributions and one pooled global-mean covariance on 12,006 observations by 3,939 coordinates. GPU FP64 covariance differed from the CPU control by at most 7.20×10^{-17}. The primary histogram has 40 bins per dimension; sensitivities retain 24, 32, 48 and 56 bins. Occupied-bin $\Delta F/k_BT = -\ln[p/p_{\max}]$ is normalised separately within each stated scope. Unoccupied bins are missing, with no pseudocount, smoothing or interpolation. The thermostat is 310.15 K in each run. Four non-overlapping 50-ns blocks, PC autocorrelation/effective-sample diagnostics and half-trajectory occupancy divergence characterise finite sampling; these are diagnostics rather than proof of equilibrium. Holo/apo condition probabilities weight the three runs equally.

SASA retains 2,001 native 100-ps observations per run and checks all 20,001 native trajectory times. Protein exposure, ligand geometric exposure and interface terms retain their explicit atom selections, radii and geometric definition. Independent runs are the comparison unit ($n = 3$ per condition); the reported run-bootstrap intervals are descriptive with only three runs. Temporal blocks and stride/point-resolution controls are retained separately.

The native membrane-MM/PBSA calculation uses AmberTools 26.0 `sander` [39] and native CHAMBER topologies: 21,460 complex atoms, 21,384 receptor atoms and 76 ligand atoms. The receptor includes 1,313 amino acids, native glycosylation (16 glycan residues/561 glycan atoms) and 1,310 CMAP terms. Protein HSD/HSE/HSP labels retain their source microstates. Same-frame complex, receptor and ligand coordinates use membrane-native placement and retained charge/bonded terms; the explicit bilayer is represented by the stated continuum membrane model for this endpoint. The dielectric settings are solute 1, membrane 7 and solvent 78.5, with pore treatment `poretype=1`. Explicit catalytic waters are not present in this continuum endpoint. Grid/ordinary-fine/native-reference controls assess the implemented solver, not experimental membrane physics or catalytic-water energetics.

The final endpoint analysis samples 50–200 ns at 1-ns spacing: 151 correlated snapshots per independent holo run and 453 total. The earlier 5-ns and 10-ns grids are exact subsets of the same coordinates and outputs; all 93 estimates in the 5-ns subset reproduce their earlier arithmetic summaries. Between-run uncertainty uses the three run means and a Student-t interval with two degrees of freedom. Complete non-overlapping partitions are [50,100), [100,150) and [150,200] ns, containing 50, 50 and 51 snapshots per run. Within-run moving-block bootstraps use 30- and 50-ns overlapping blocks without artificial periodic wrapping; they are kept separate from independent-run inference. Trapezoidal time-weighted means assess nested-grid spacing sensitivity. Entropy is excluded, so these quantities are endpoint energy estimates rather than complete binding free energies.

Supported amino-acid decomposition terms retain the 1,313 native protein residue identities and their internal, van der Waals, electrostatic, polar and supported SAS components. Each residue is summarised by independent run and by the complete 50/50/51-snapshot partitions; descriptive three-run Student-t intervals accompany the supported sums. Global nonpolar energy remains unassigned to individual residues. Unsupported zero values in the `idecomp=2` TOTAL channel are excluded from interpretation. Figure S18 displays the 16 largest absolute mean supported amino-acid terms as an exploratory selection; the source tables retain every residue. These components are not full residue-level binding free energies or experimentally validated hotspots.

Extended simulation results

All six independent trajectories completed 200 ns and their run-specific major numerical and structural checks. The shared PCA basis explained 31.26% and 13.60% of the global variance in PC1 and PC2. The projected two- and three-dimensional occupancy landscapes retained distinct run and temporal-block patterns (main Figure 7c,d; Supplementary

Figures S8–S12). PC1 descriptive effective sample sizes ranged from 4.17 to 28.83; first-half versus second-half Jensen–Shannon divergences ranged from 0.293 to 0.520 bits. These diagnostics indicate finite sampling and run differences; they do not establish convergence or kinetic barriers.

Over 50–200 ns, the equal-run mean pocket heavy-atom RMSD after PSEN1 fitting was 0.216 nm in holo and 0.343 nm in apo runs (main Figure 7a). The unpaired holo-minus-apo difference was -0.128 nm (approximate Welch-t 95% interval, -0.178 to -0.077). Whole-complex-fit Cα RMSD was 0.279 and 0.312 nm, respectively; its difference interval included zero. Mass-weighted protein R_g was 4.005 and 4.011 nm, with a difference interval also including zero. The mean ligand heavy-atom RMSD after protein fitting was 0.245 nm (descriptive between-run Student-t 95% interval, 0.046–0.445; main Figure 7b). Holo runs averaged 2.852 direct protein–ligand hydrogen bonds and 0.509 residues connected through the same explicit water molecule per frame. Individual runs, residue flexibility, contact occupancies and between-run intervals are shown in Supplementary Figures S19–S23. These reference-dependent structural measures describe the simulated models; the pocket difference does not establish experimental affinity or general protein stability.

Protein SASA was higher on average in holo than apo by 4.565 nm^2 over 0–200 ns (descriptive three-run bootstrap 95% interval, -3.116 to 12.246) and 3.892 nm^2 over 50–200 ns (-3.763 to 11.845). Both intervals crossed zero. Per-run protein SASA, ligand geometric exposure/interface areas, temporal blocks and numerical-resolution controls are in Supplementary Figures S13–S16. These estimates do not support a significant reduction in exposure or a general stability advantage.

Across the 453 membrane-MM/PBSA estimates, the three holo run means were -8.575, -20.189, -0.022 kcal/mol. Their equal-run mean was -9.595 kcal/mol (descriptive Student-t 95% interval with two degrees of freedom, -34.739 to 15.549 kcal/mol; main Figure 7e). The interval includes zero and entropy was excluded, so the estimates do not establish energetically favourable binding under this model. Time-weighted 1-minus-5-ns differences were $+1.282$, $+0.362$ and $+0.768$ kcal/mol. Residual spacing sensitivity and temporal-block differences qualify the estimates (Supplementary Table S3 and Figures S17–S18). Supported amino-acid contributions, including their run and block variability, retain the native residue mapping; global nonpolar energy is reported separately. These endpoint energies do not estimate experimental affinity or potency.

Main Figure 7 display conventions. RMSD traces display the original 100-ps observations without smoothing; dashed vertical lines mark the 50-ns start of the summary window. Ligand heavy-atom RMSD is relative to each holo run's initial production frame after PSEN1 fitting. Occupancy landscapes use the common 1,313-Cα basis and equal run weights; unvisited bins remain missing and colour and height scales are shared. These finite-sampling projections do not estimate kinetic barriers or establish convergence. The endpoint-energy panel uses 151 snapshots per holo run at 1-ns spacing over 50–200 ns (453 total); its interval is calculated across three independent run means (Student-t, df=2), and entropy is excluded.

Supplementary tables

Supplementary Table S1. Software version evidence

Version documentation combines retained execution manifests and version exports, installation and workflow specifications, and author-confirmed contemporary GitHub installations cross-checked against versioned repository metadata. The provenance column identifies the source for each workflow.

Software	Version	Workflow/provenance
Matplotlib [20]	3.11.0	Submitted PDF metadata and retained plotting manifest.
Python; NumPy [21]; pandas [22]	3.12.5; 2.4.6; 2.3.3	Revision plotting and donor-summary execution manifests.
SciPy [27]; statsmodels [26]	1.16.3; 0.14.6	Exact-cell donor-summary execution manifest.
PyMuPDF [23]; pypdf [24]	1.27.2.3; 6.11.0	Retained figure-layout manifest; PDF-layer handling.
Scanpy [40]; AnnData [41]	1.11.5; 0.11.4	Saved spatial-workflow version export; the separate revision setup pins AnnData 0.12.14.
harmonypy	0.0.10	Pinned revision-environment installation specification.
Leiden backend: Python igraph	1.0.0	Scanpy uses its igraph backend. Version reconstructed from the authors' latest-version confirmation and official release chronology.
umap-learn	0.5.12	Author-confirmed contemporary version, cross-checked against the official release history.
emptyDrops / DropletUtils	1.26.0	Retained DropletUtils installation archive and linked local-annotation setup script.
DecontX / celda	1.26.0	Version specified in the retained decontamination workflow source.
CellOracle [3]	0.20.0	Archived full-object reconstruction source and software specification.
CellRank [7]	2.0.7	Archived trajectory launch scripts and linked pipeline specification.
Cicero [4]	1.3.9	Retained installation and package-loading log.
decoupler [5]	2.1.4; 2.1.6	Saved spatial-workflow export: 2.1.4; submitted external-transfer workflow specification: 2.1.6.
SCENIC+ [9]; Snakemake [11]	1.0a2; 8.5.5	Retained installation log for the SCENIC+ environment.
R [25]; glmmTMB [1]	4.4.3; 1.1.14	Retained revision composition-model refit manifest.
edgeR [12]; limma [42]	4.4.2; 3.62.2	Recovered workflow specification; limma also identified in retained session information.
RCTD / spacexr [19]	2.2.1	GitHub installation route in retained source; version reconstructed from author confirmation and historical DESCRIPTION metadata.
BANKSY / Banksy [13]	1.9.2	Author-confirmed GitHub installation; contemporary devel-branch DESCRIPTION checked.
PySAL: esda; libpysal	2.7.0; 4.13.0	Saved spatial-workflow version export.
LIANA+ [17]	1.8.0	Saved communication-workflow version export.
COMMOT [18]	0.0.3	Version specified in retained spatial-communication workflow source.
NicheNet / nichenetr [16]	2.2.1.1	Author-confirmed contemporary GitHub version, checked against historical master-branch DESCRIPTION metadata.
sccomp [2]	2.1.34	Author-confirmed GitHub installation; contemporary master-branch DESCRIPTION checked.
GROMACS [38]	2026.3	Native minimisation, EQ1–6 and production runtime records.
CHARMM [37]	47a2	Retained CHARMM-GUI builder log; local assembly and GROMACS dynamics are documented separately.
CGenFF	Program 4.0; parameters 5.0	Initial ligand stream and retained production parameter provenance.
AmberTools [39]	26.0	Retained native CHAMBER and membrane-PB solver/runtime records.
PLIP [43]; PyMOL [44]; MDAnalysis [45, 46]	3.0.1; 3.1.1; 2.10.0	Recorded rendering/toolchain and postprocessing. The exact PyMOL 3.1.1 build record dates to 2024.

Supplementary Table S2. O6U analogy penalties of at least 10

Term	CGenFF identifier	CCD identifier	Penalty
Charge	C8	C24	11.959
Charge	C20	C27	16.329
Charge	C23	C31	32.293
Charge	C24	C32	26.346
Charge	N2	N05	14.433
Charge	N4	N07	25.561
Charge	N5	N08	32.824
Angle	N5–C23–N4	N08–C31–N07	28
Dihedral	N2–C20–C23–N4	N05–C27–C31–N07	29
Dihedral	N4–C23–N5–C24	N07–C31–N08–C32	35.5
Dihedral	N5–C23–N4–C8	N08–C31–N07–C24	28
Dihedral	N5–C23–N4–H41	N08–C31–N07–H43	28

Dihedral rows group Fourier terms for the same atom sequence; the N5–C23–N4–C8 entry contains three terms. Penalties in the 10–50 range warrant basic validation. These values are not confidence intervals or binding energies.

Table S3. Membrane-MM/PBSA independent-run and temporal-block means. Energies are in kcal/mol and exclude entropy. The three runs are independent; snapshots and within-run blocks are correlated. The last column is the trapezoidal time-weighted 1-minus-5-ns difference.

Run	50–200 ns	[50,100)	[100,150)	[150,200]	1–5 ns
MD 1	-8.575	-9.700	-8.308	-7.734	1.282
MD 2	-20.189	-21.276	-22.250	-17.101	0.362
MD 3	-0.022	-0.054	0.684	-0.684	0.768

Equal-run mean: -9.595; between-run 95% interval: -34.739 to 15.549 (Student-t, df=2).

Supplementary figures

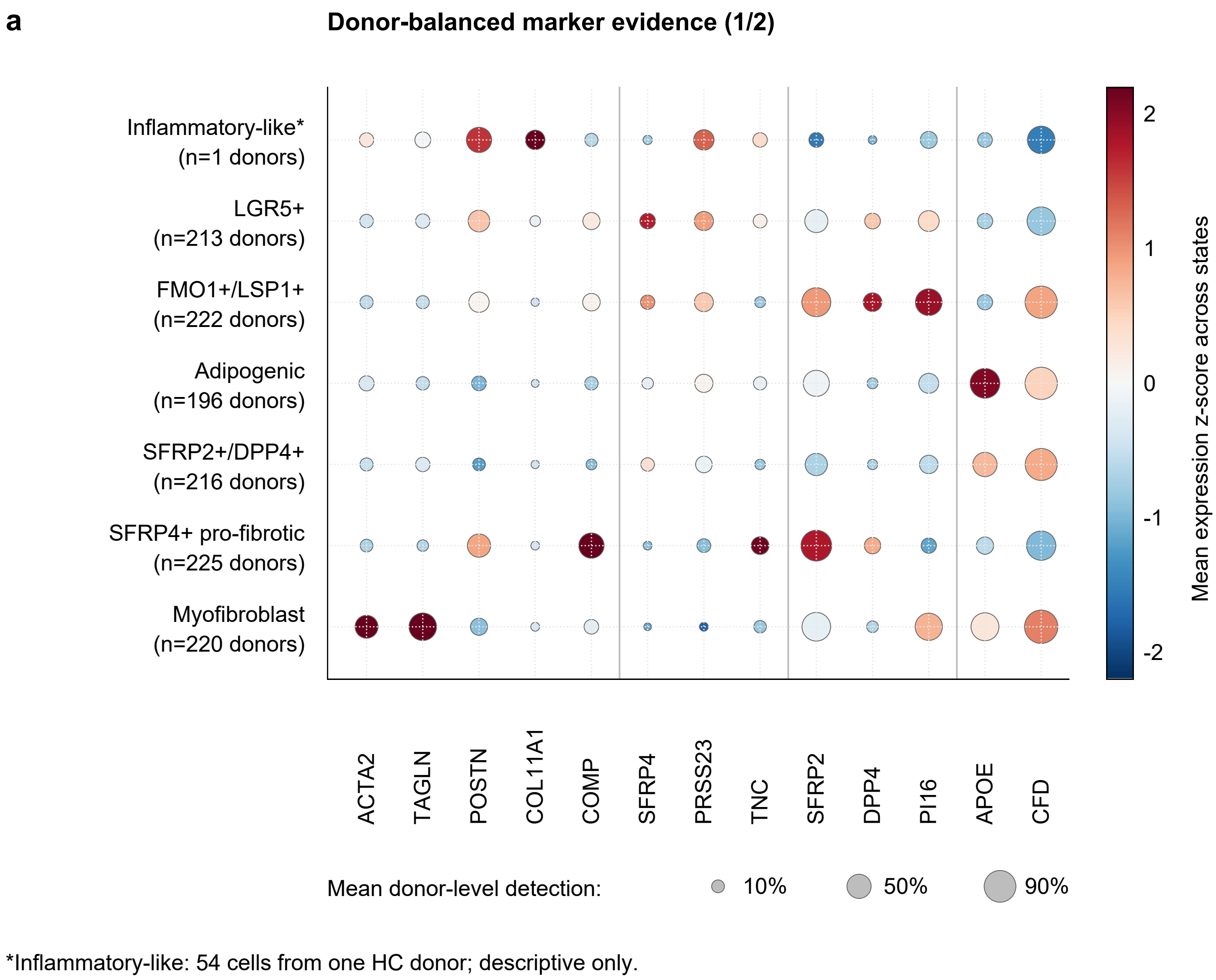

Figure S1. Donor-balanced marker evidence. Inflammatory-like is descriptive.

b

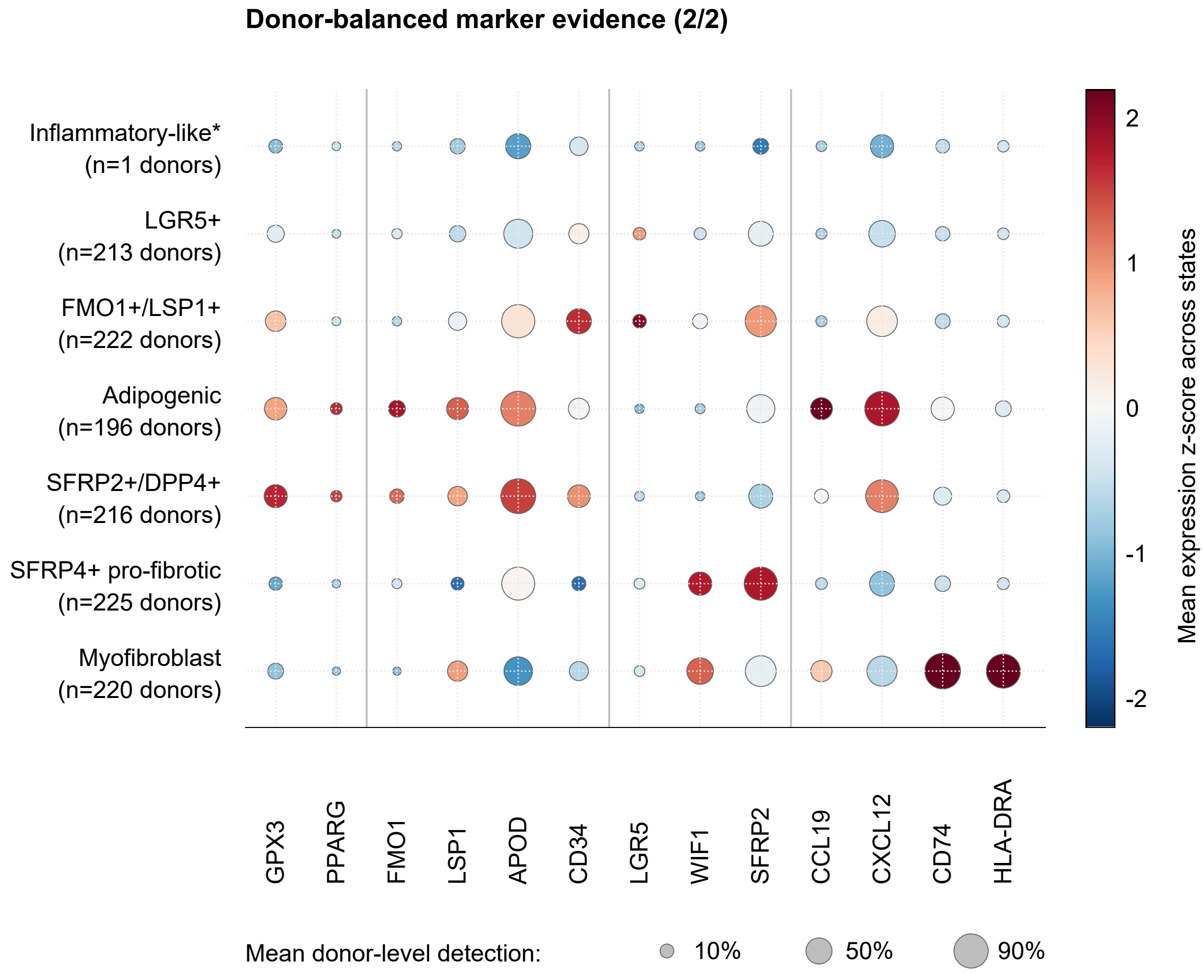

*Inflammatory-like: 54 cells from one HC donor; descriptive only.

Figure S1 (continued). Donor-balanced marker evidence. Inflammatory-like is descriptive.

a HES1 selected TF–region–gene triplets

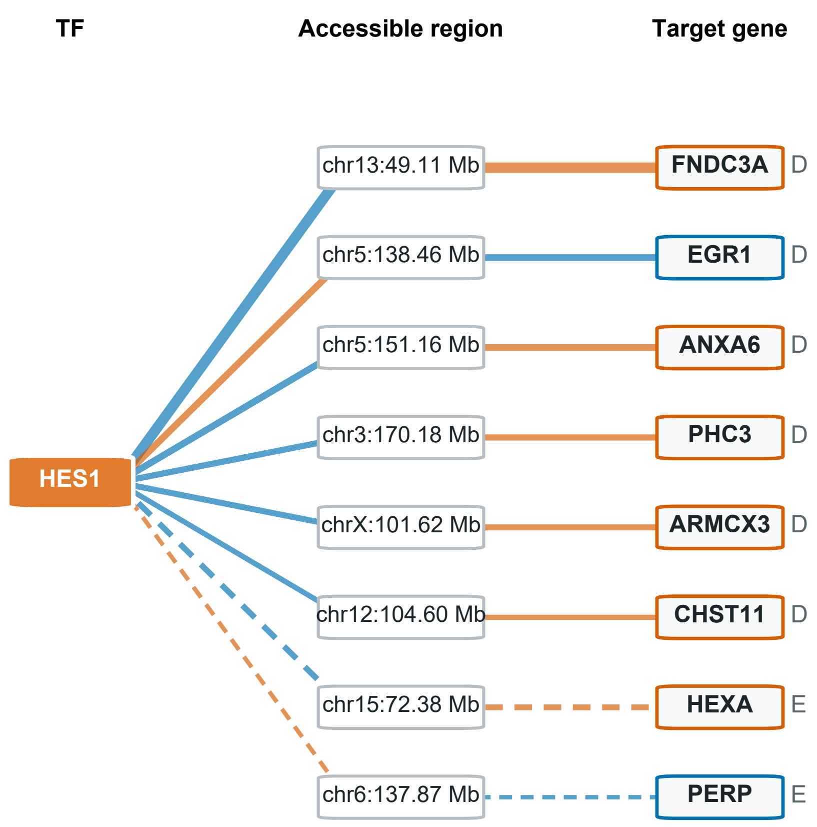

Orange: positive association; blue: negative association.
Solid: direct; dashed: extended. D/E labels retain archived scope.

Edge width retains the product of within-run importance/rank percentiles.

Figure S2. Selected HES1/RBPJ triplets, association signs and within-run ranks.

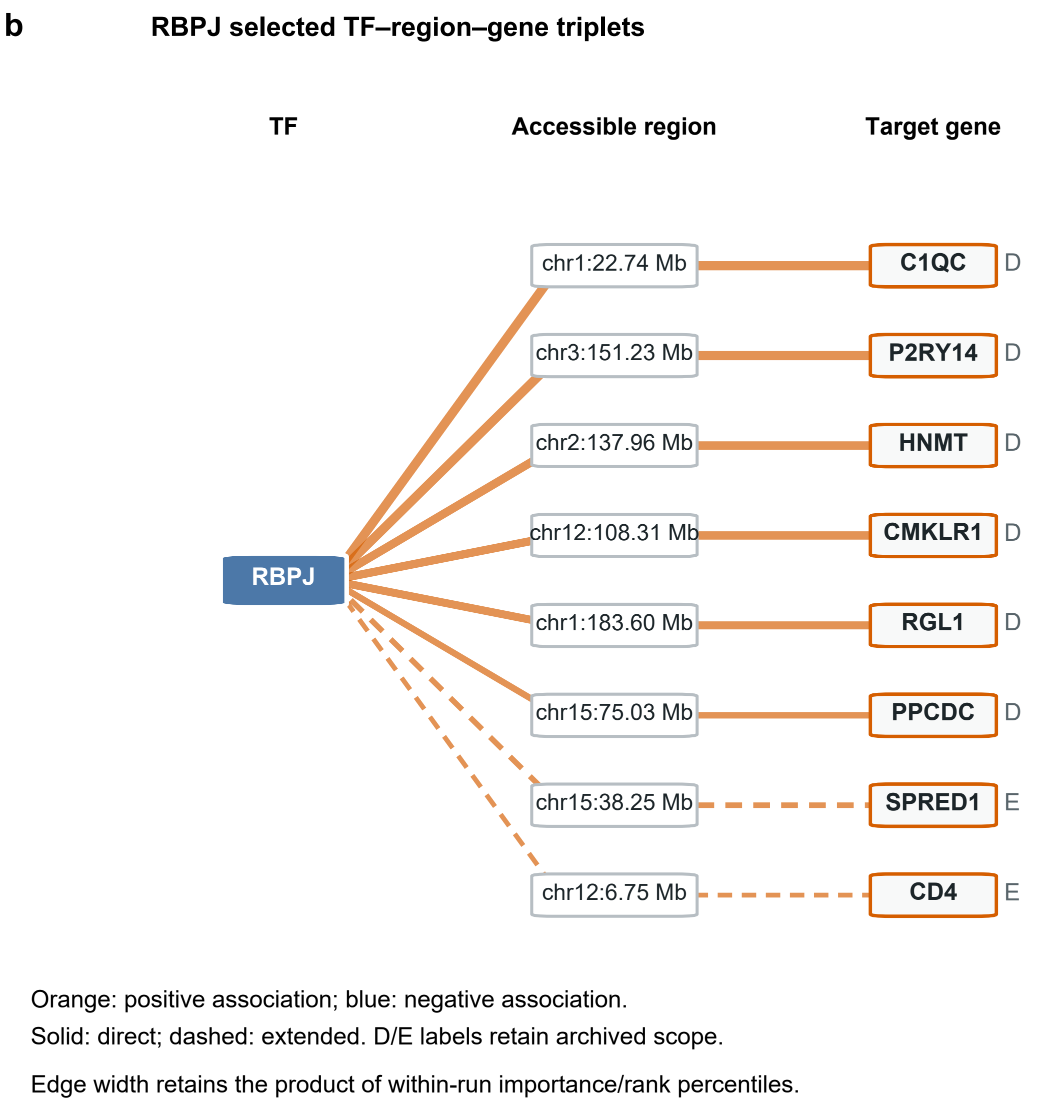

Figure S2 (continued). Selected HES1/RBPJ triplets, association signs and within-run ranks.

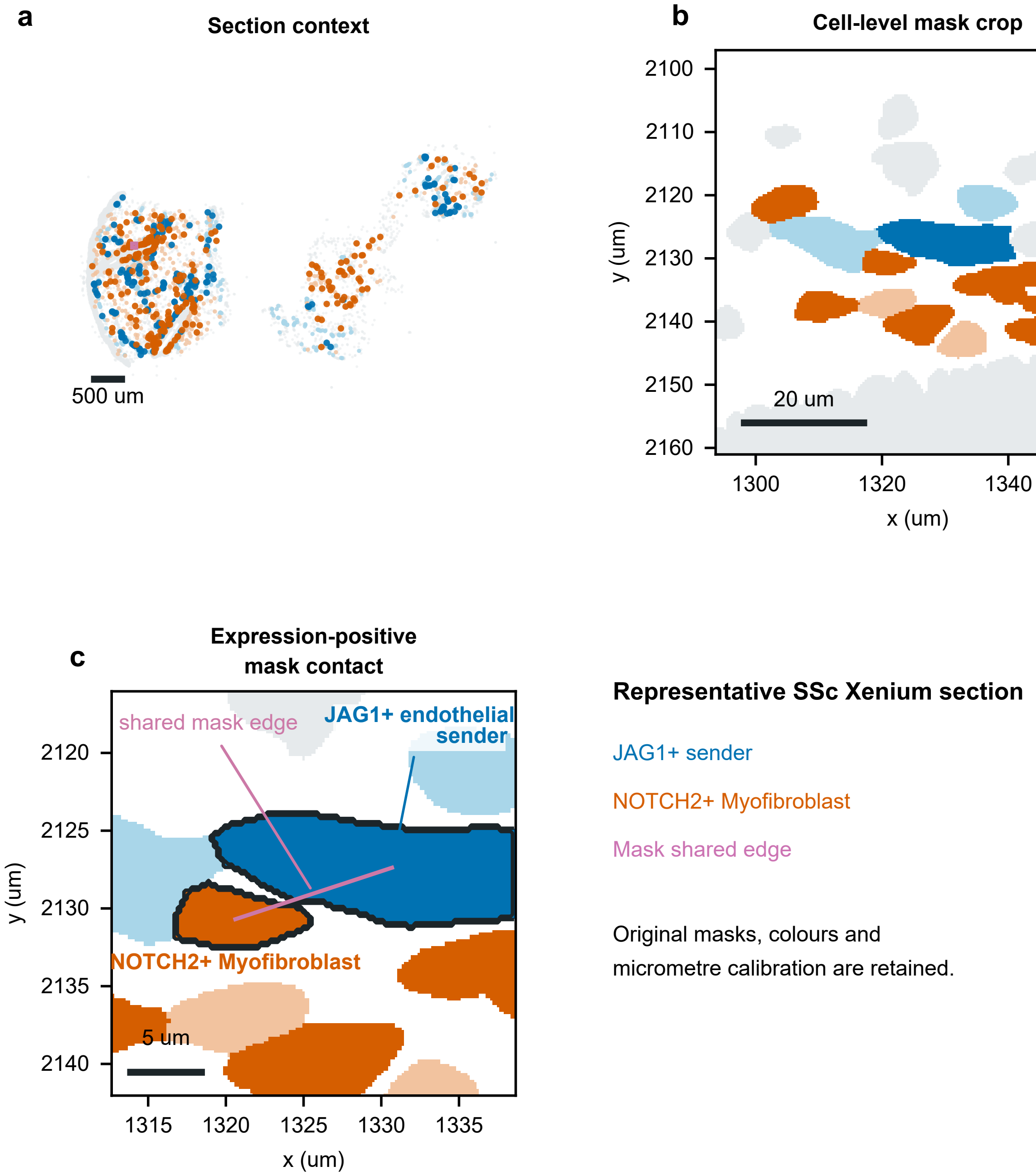

Figure S3. Representative expression-positive shared-mask-edge contact; an example does not establish enrichment.

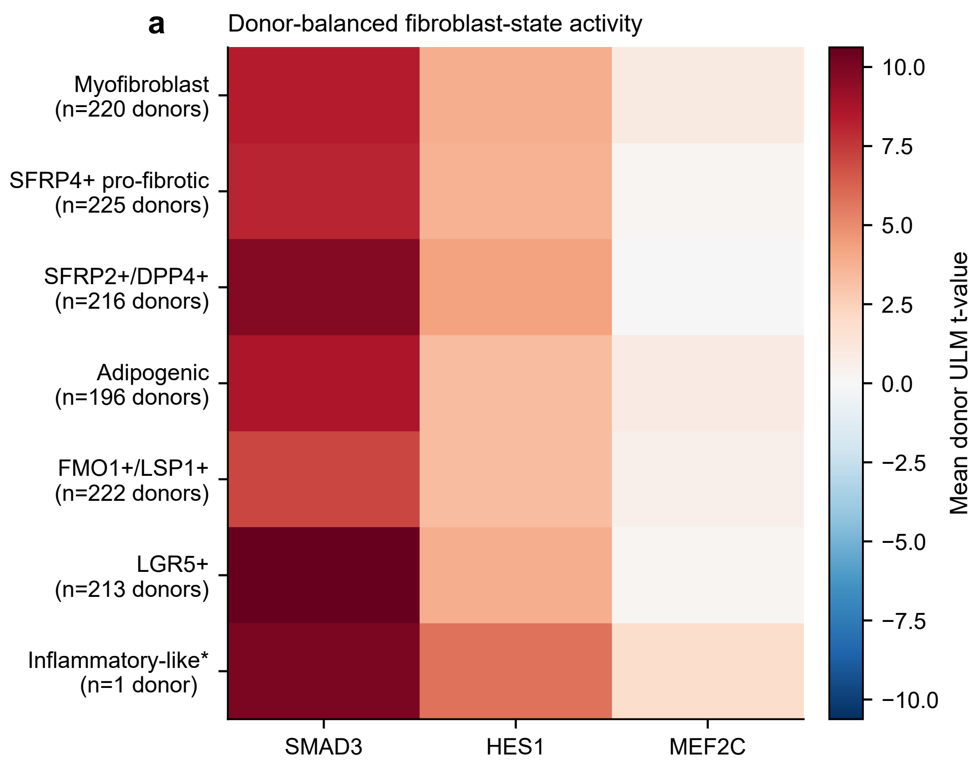

*Inflammatory-like: 54 cells from one HC donor; descriptive only.

Figure S4. Donor-balanced descriptive mean ULM activities for HES1, SMAD3 and MEF2C across seven fibroblast states. Cell activities are averaged within donor/state before equally weighting donors. State-specific donor counts differ; Inflammatory-like has one HC donor and is descriptive. The 3,999 unassigned fibroblasts lack saved activity and are omitted. These summaries are not disease contrasts.

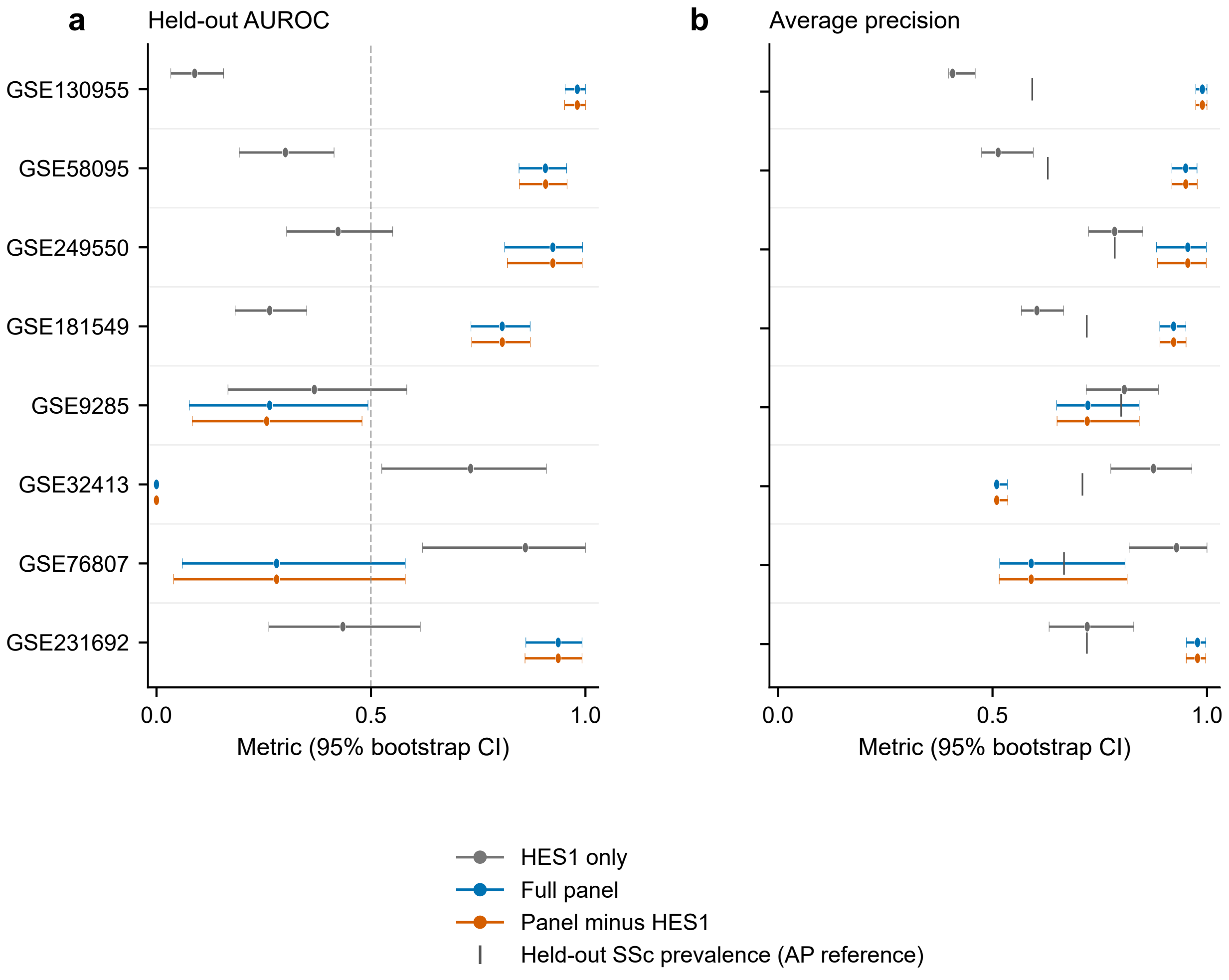

Figure S5. Held-out discrimination/calibration for HES1-only, full-panel and HES1-removed models.

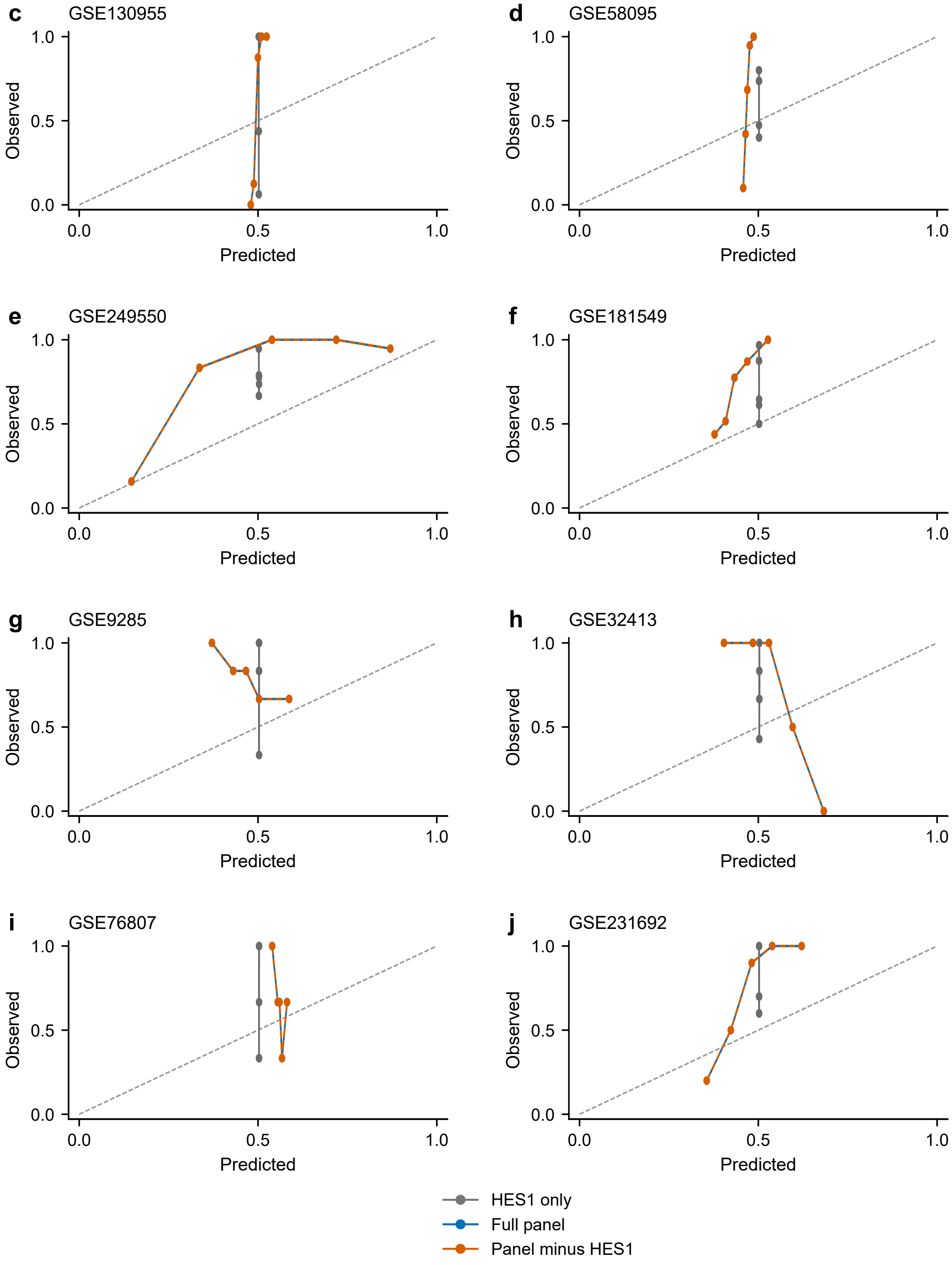

Figure S5 (continued). Held-out discrimination/calibration for HES1-only, full-panel and HES1-removed models.

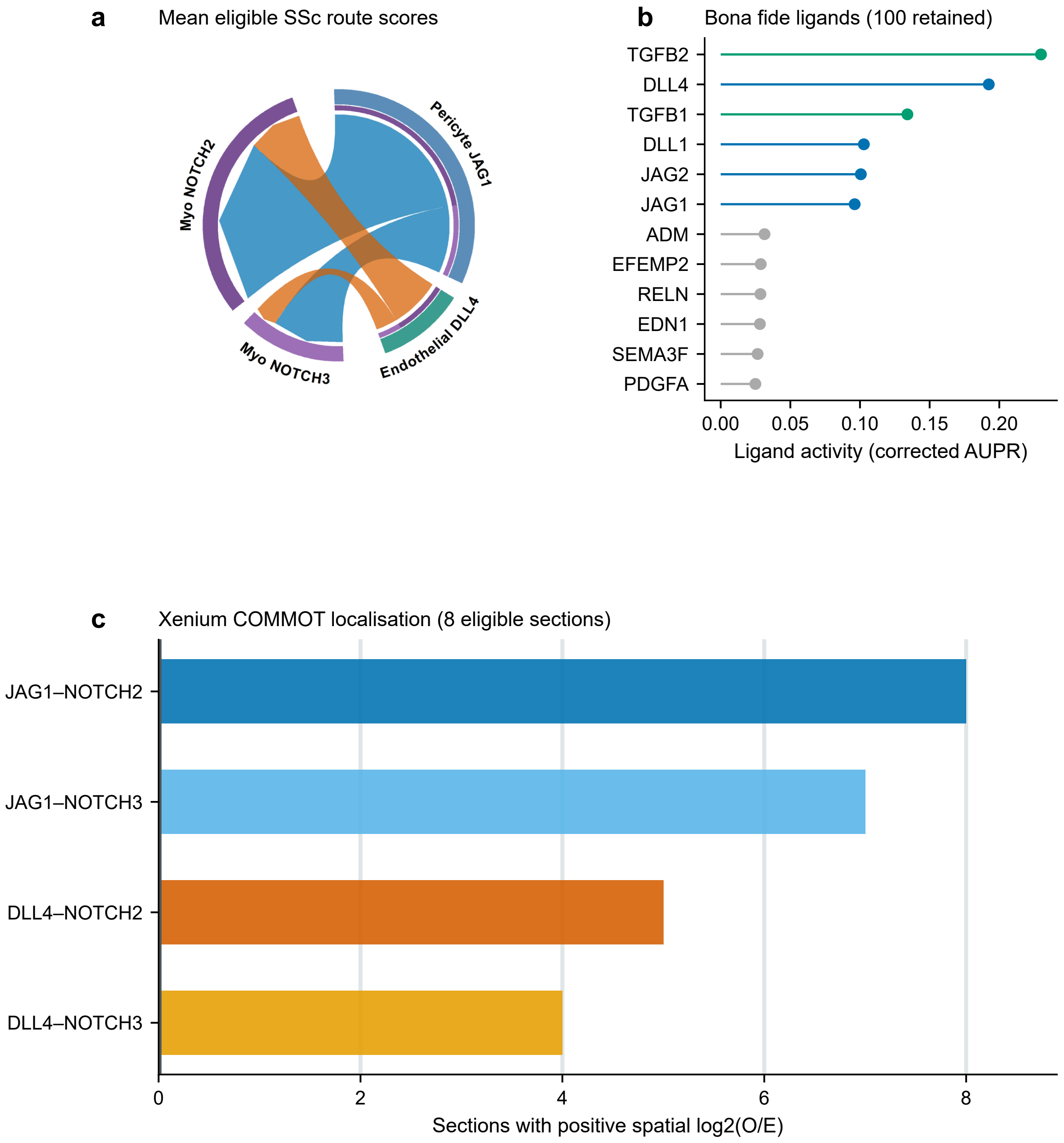

Figure S6. Auxiliary route display, NicheNet ranks and Xenium transport localisation.

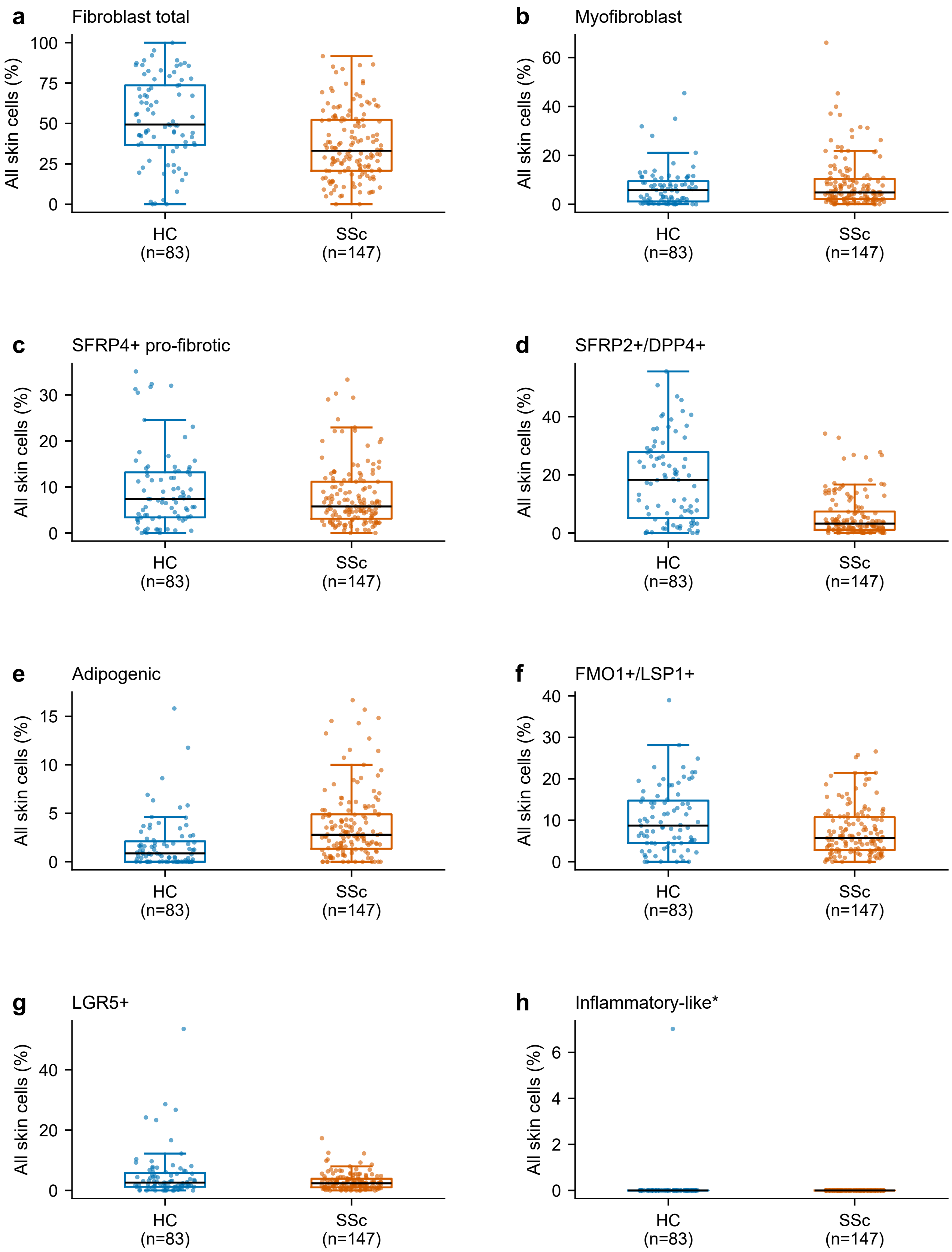

Figure S7. Donor fibroblast-state composition with all retained skin cells as denominator.

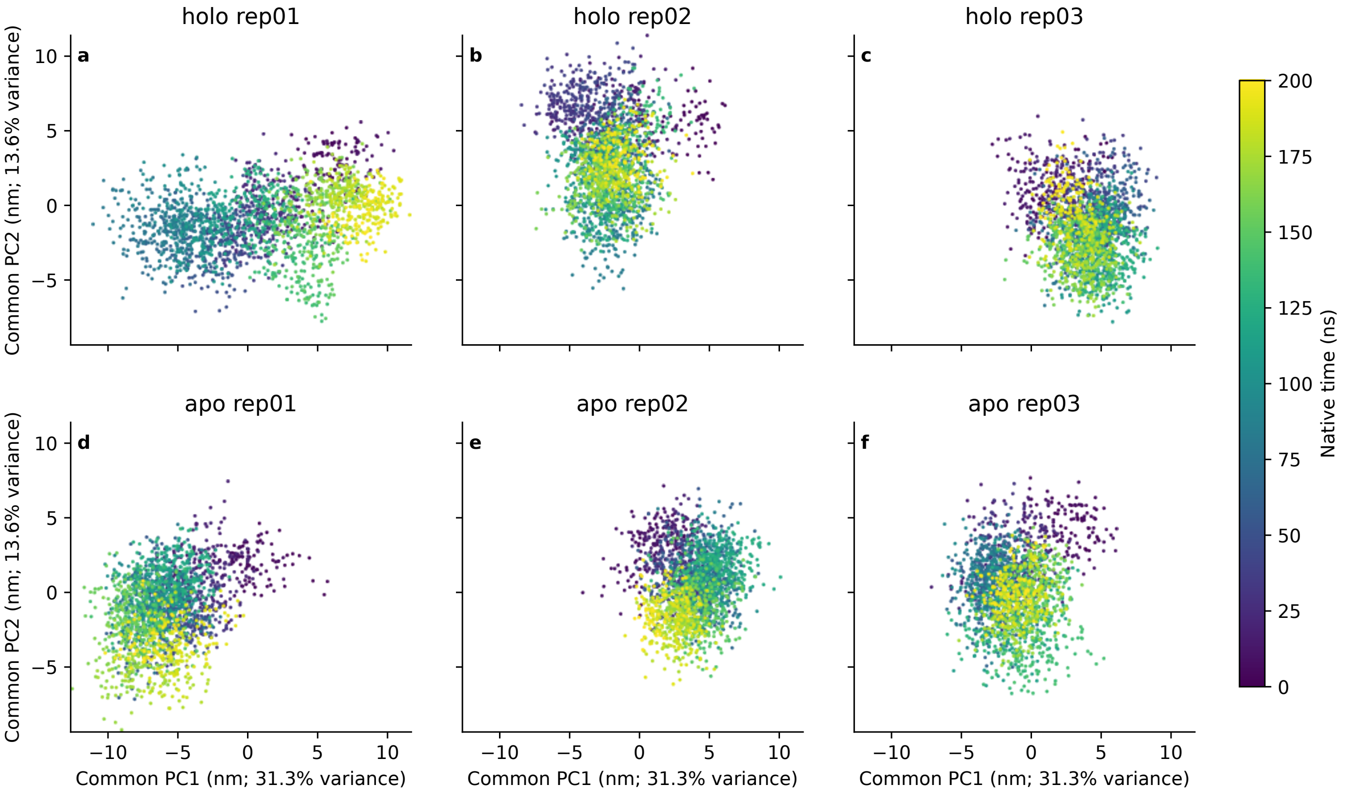

Figure S8. Six-run common-basis principal-component trajectories. Independent runs share one matched-atom mapping and covariance basis.

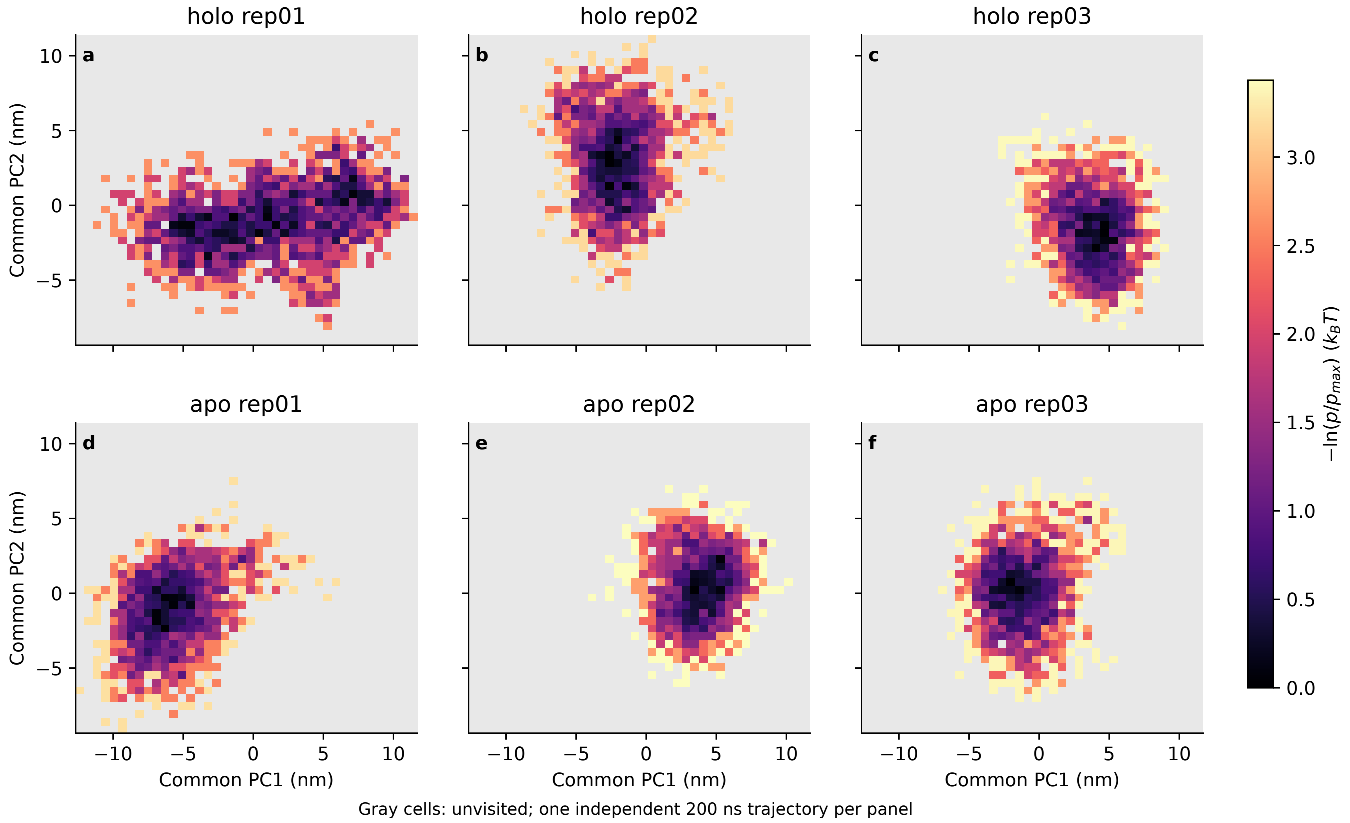

Figure S9. Six-run projected occupancy landscapes. Unsupported histogram bins remain missing; each surface has its own additive zero.

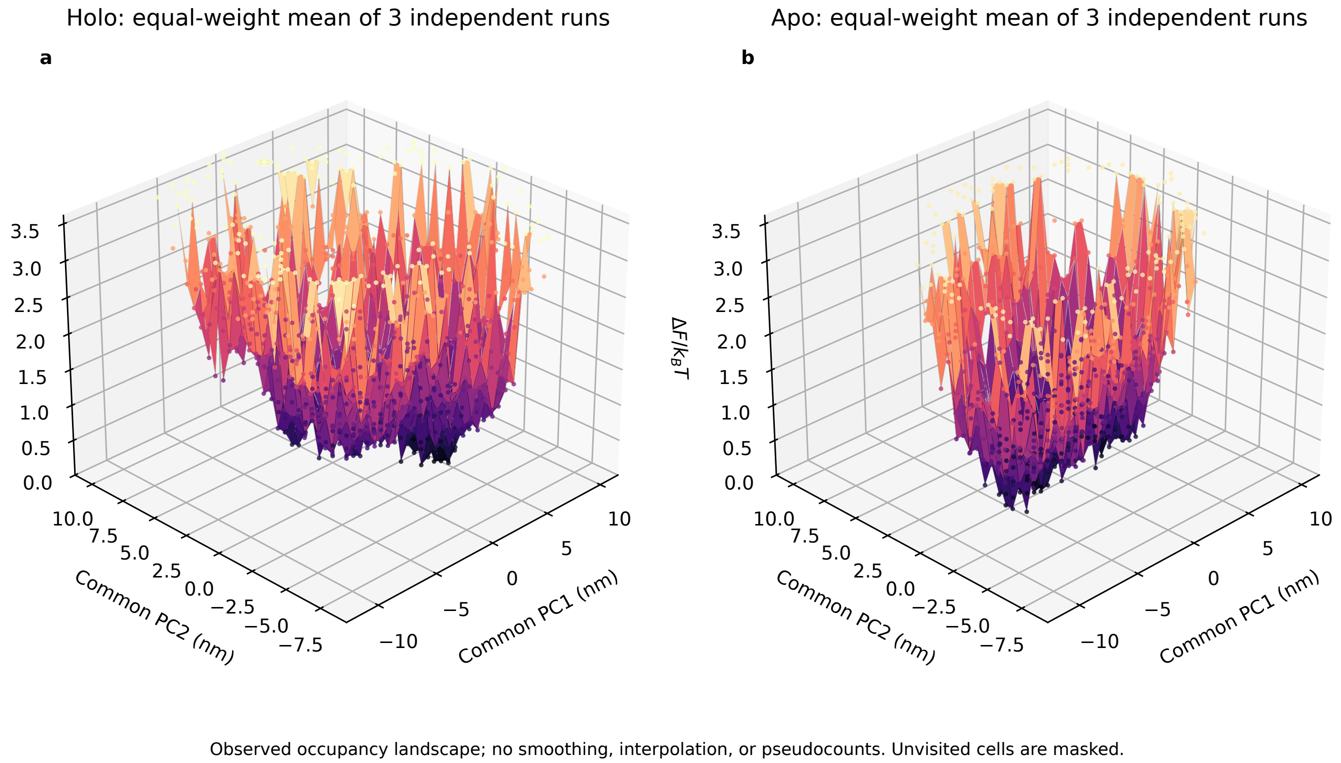

Figure S10. Three-dimensional holo and apo projected occupancy landscapes. These are conformational occupancy surfaces, with finite sampling and no affinity or kinetic-barrier inference.

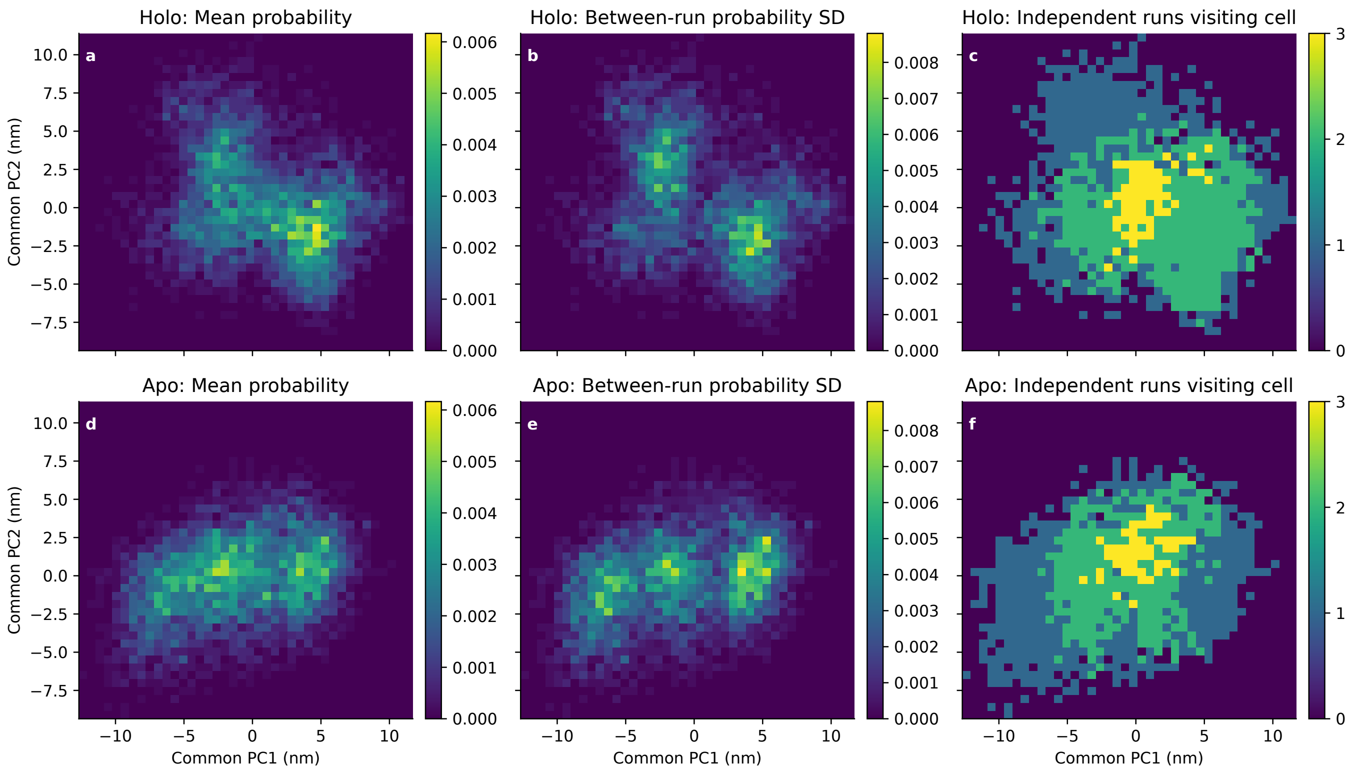

Figure S11. Independent-run and temporal-block occupancy support. Four 50-ns blocks describe within-run sampling; three runs per condition define comparison units.

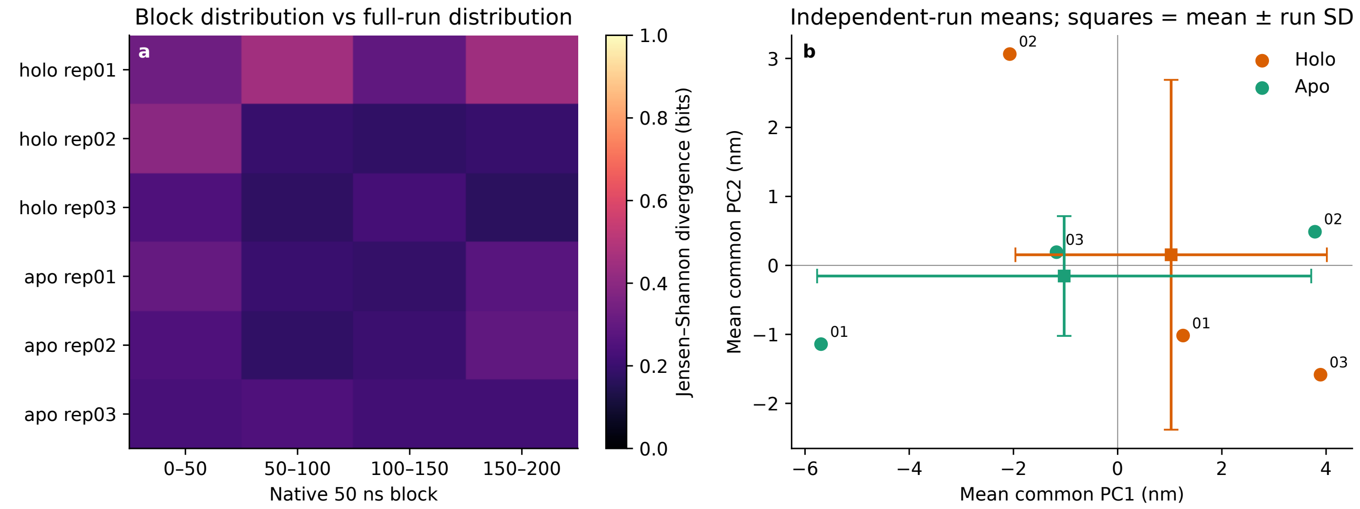

Figure S12. Sampling and histogram-resolution diagnostics. Autocorrelation and occupancy differences qualify interpretation rather than certify convergence.

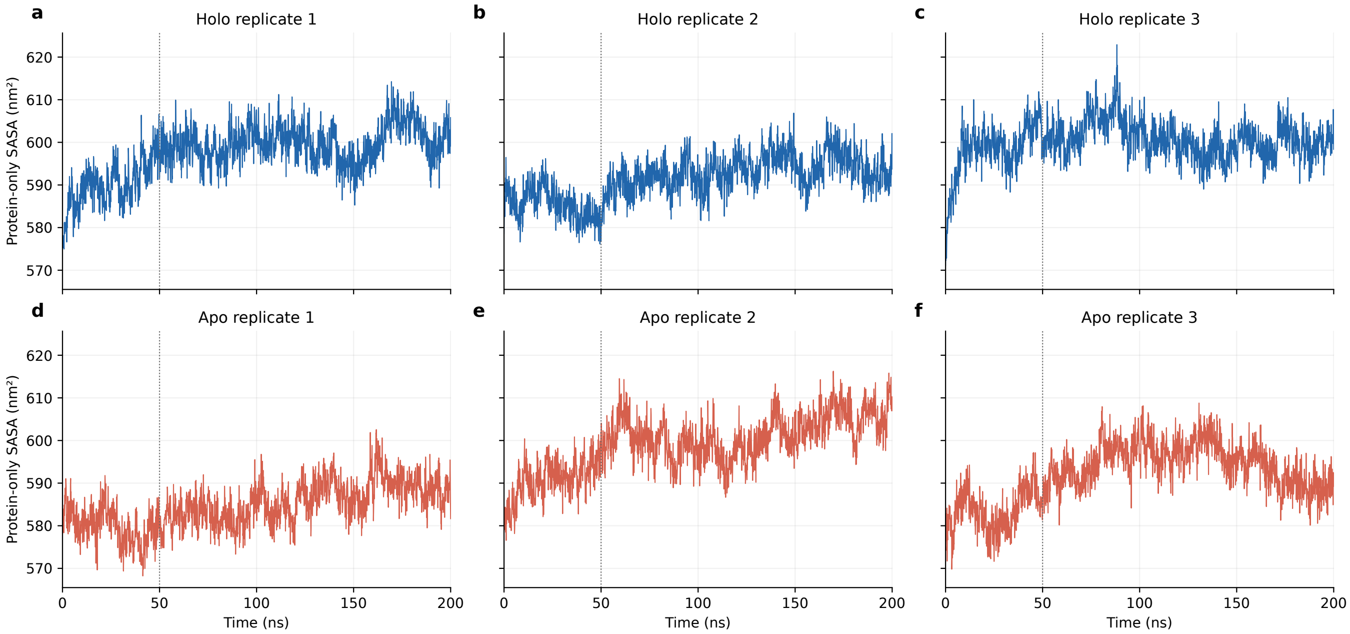

Figure S13. Protein solvent-accessible surface area for each of six independent runs.

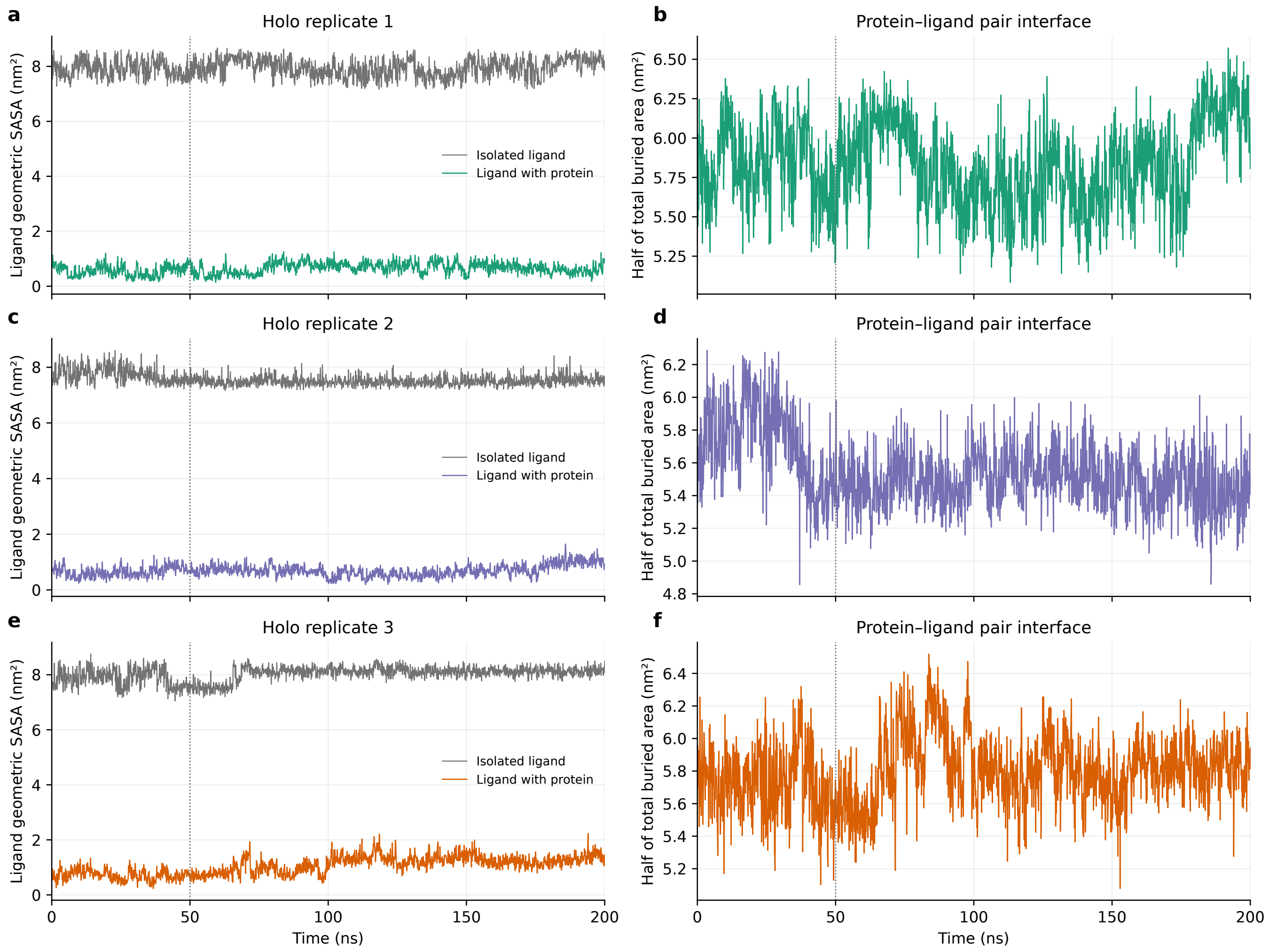

Figure S14. Holo ligand geometric exposure and interface areas, with explicit source selections.

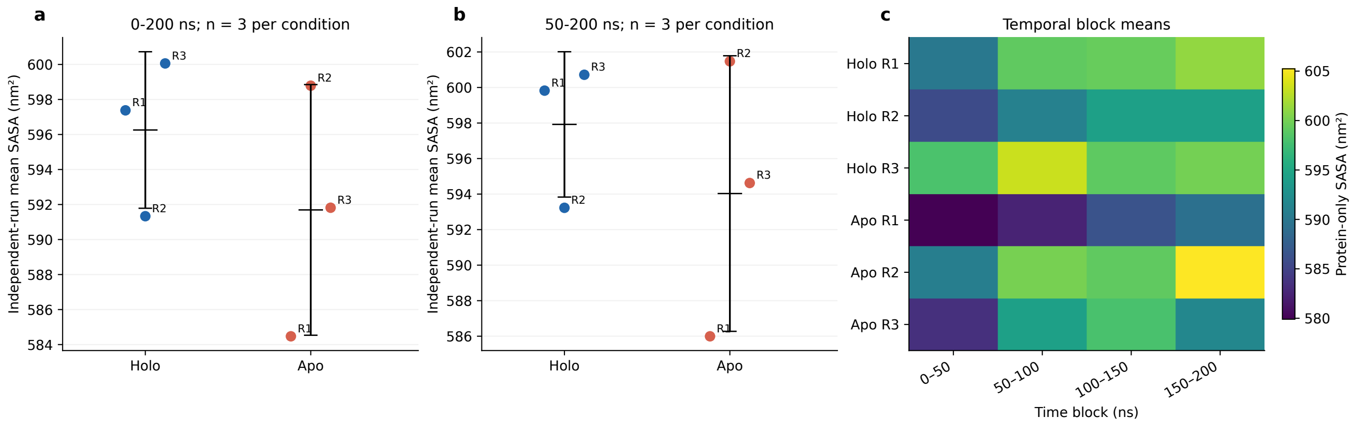

Figure S15. Independent-run and 50-ns-block SASA summaries. Three-run bootstrap intervals are descriptive.

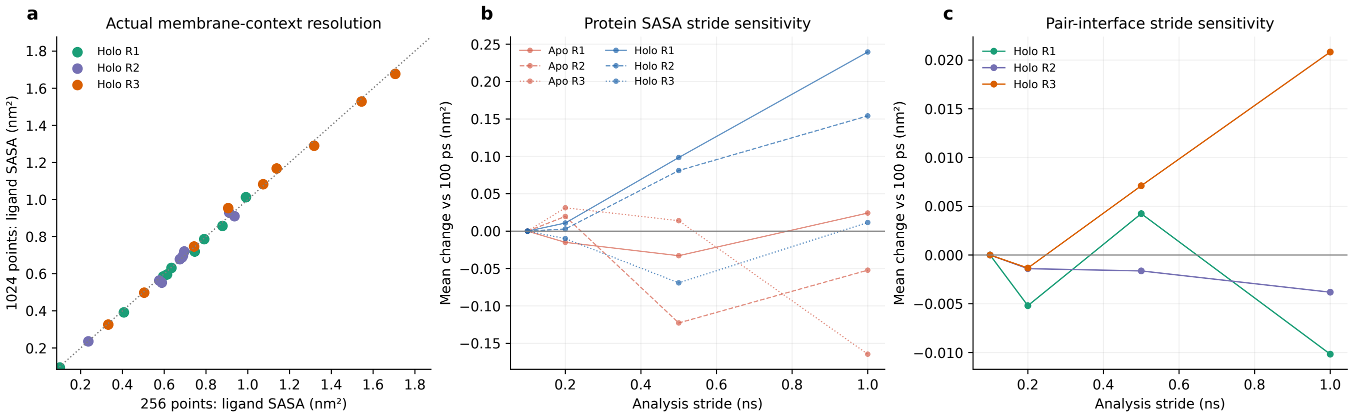

Figure S16. SASA native-stride and point-resolution sensitivity. Numerical controls retain the source geometric definitions.

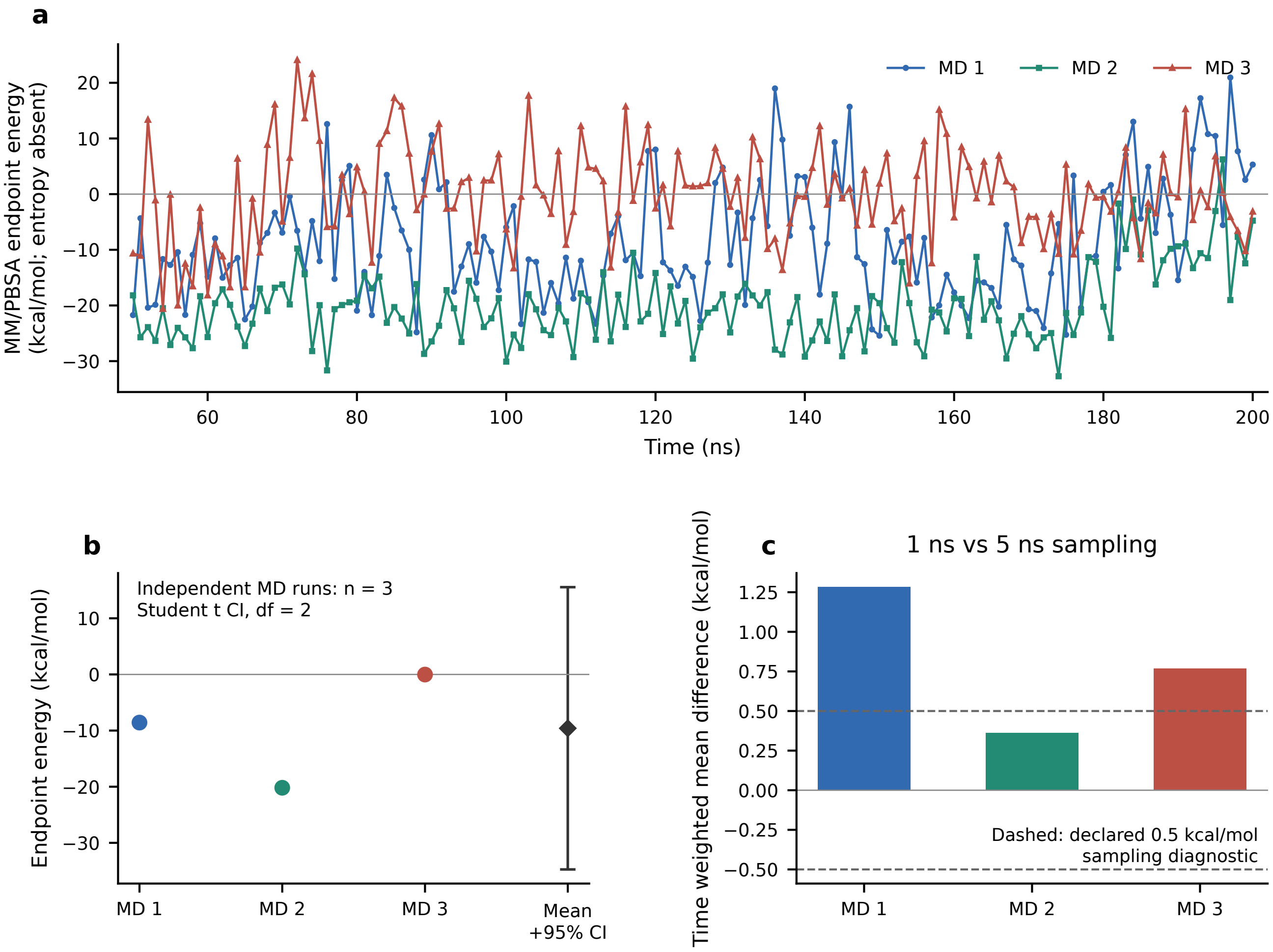

Figure S17. Membrane-MM/PBSA endpoint estimates from three independent runs, each sampled at 151 times over 50–200 ns. (a) Per-run energy traces. (b) Run means and the equal-run mean with a Student-t 95% interval (two degrees of freedom). (c) Trapezoidal time-weighted 1-minus-5-ns differences; dashed lines mark the predeclared 0.5-kcal/mol spacing diagnostic. Entropy is excluded and the between-run interval includes zero.

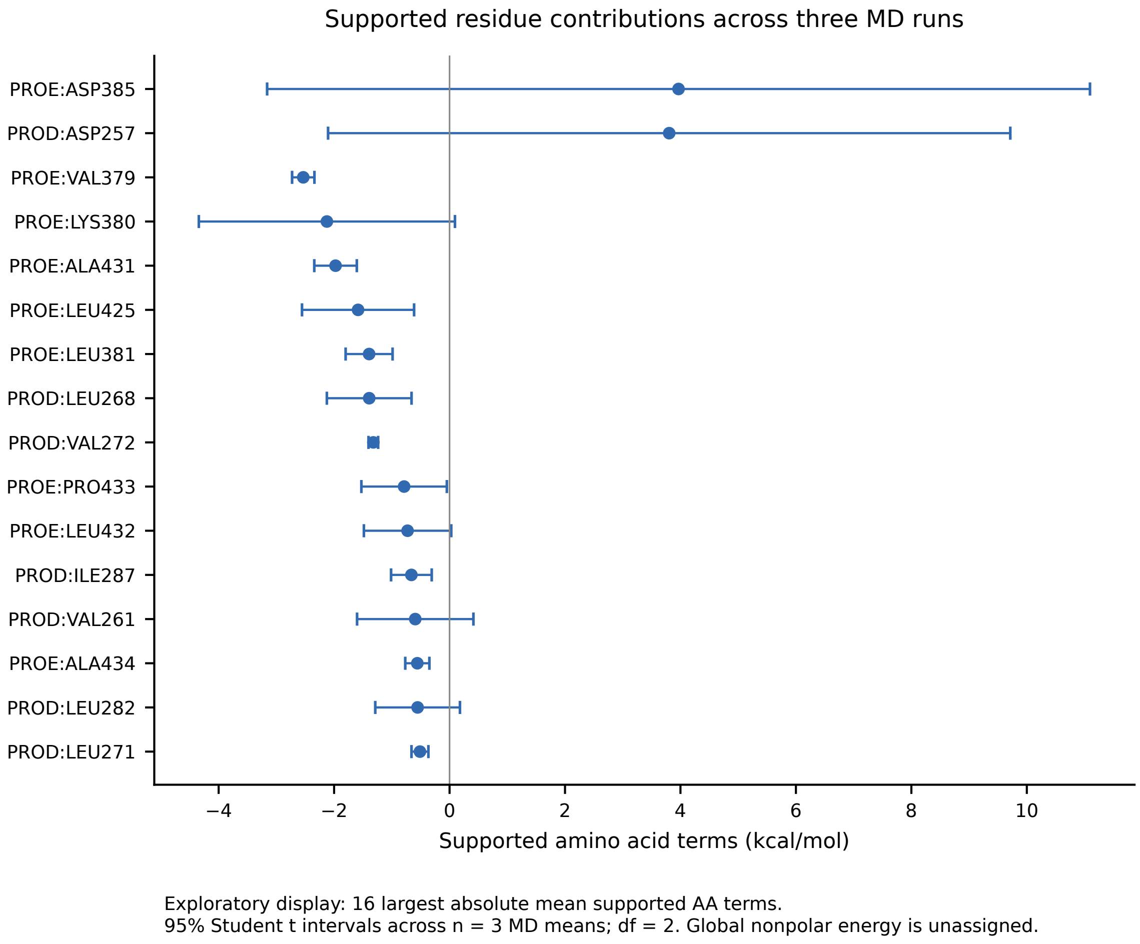

Figure S18. Supported amino-acid endpoint components from all 453 snapshots. Points and bars show equal-run means and descriptive three-run Student-t 95% intervals. The 16 largest absolute means are displayed for exploration; all 1,313 protein residues and complete temporal-block summaries are retained in the source tables. Global nonpolar energy is separate, so the plotted sums are not full per-residue TOTAL energies or experimentally established hotspots.

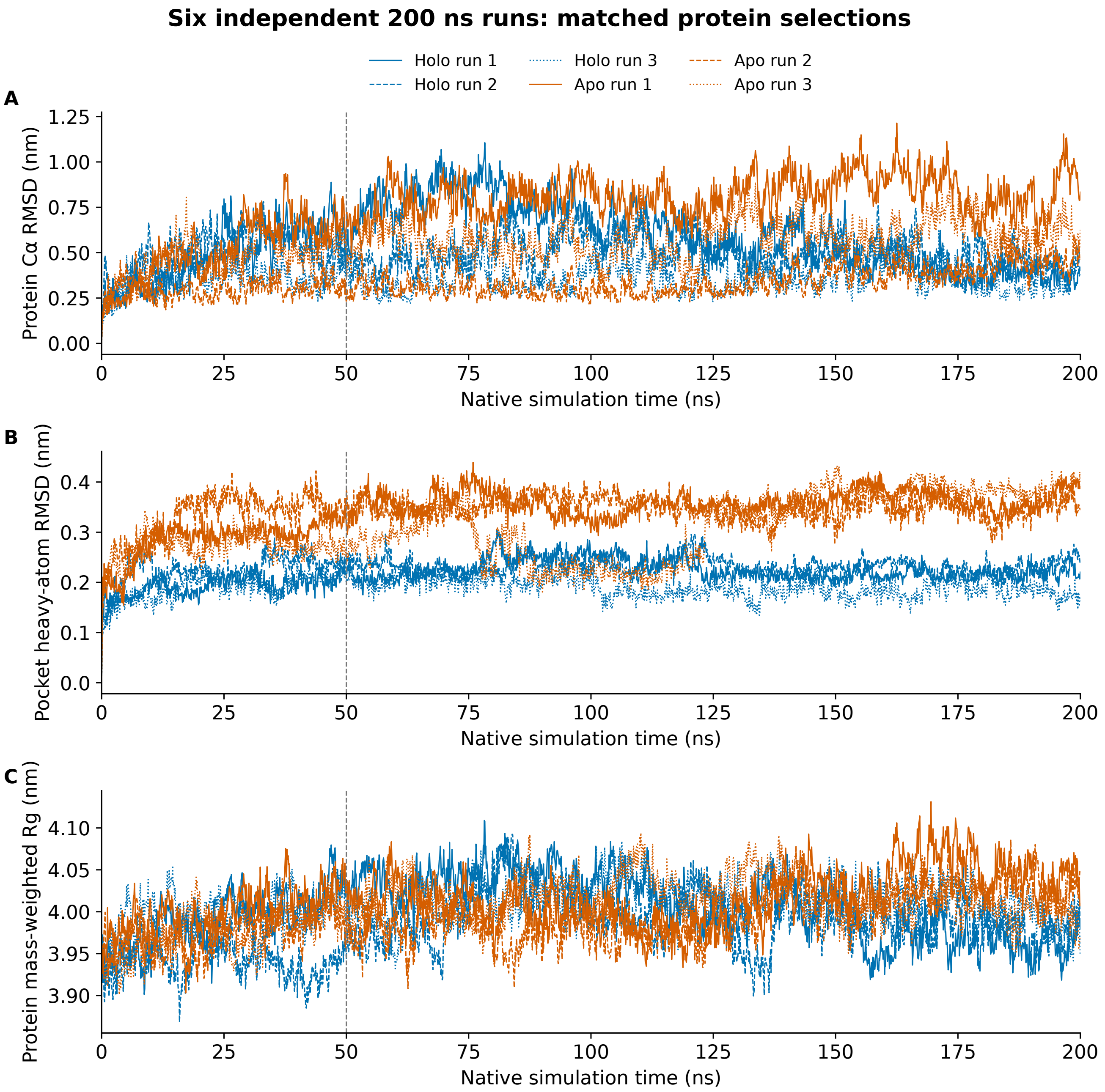

Figure S19. Structural dynamics of six independent runs. Traces display 100-ps observations from metrics calculated every 10 ps. Each run uses its own EQ6 reference; atom selections and fitting definitions are matched between conditions (Methods S8). Protein R_g uses the production TPR masses after hydrogen mass repartitioning. The vertical line at 50 ns marks the reporting window.

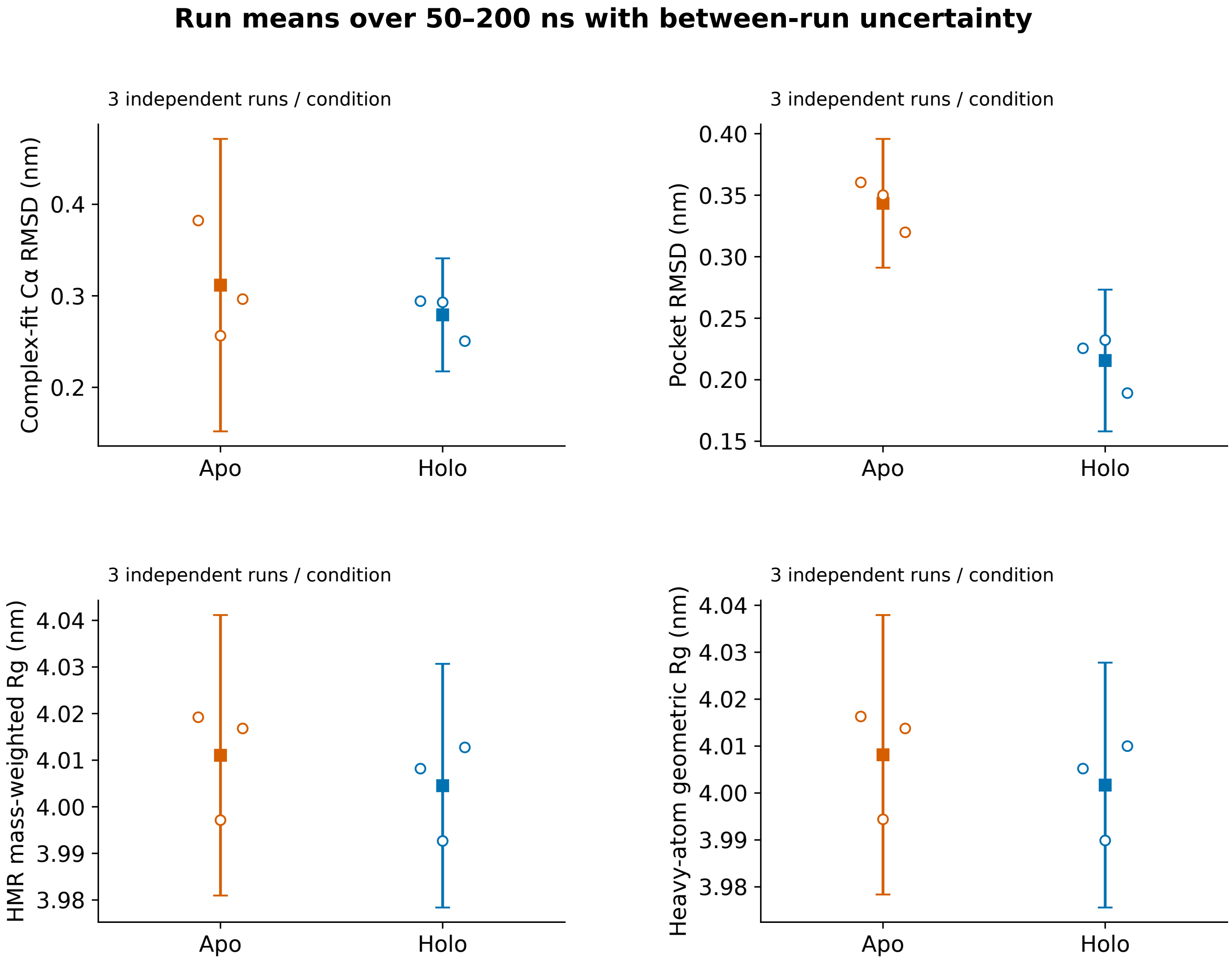

Figure S20. Run-level structural summaries over 50–200 ns. Circles represent the three independent run means per condition. Squares and bars show the equal-run mean and descriptive Student-t 95% interval with two degrees of freedom. Holo and apo runs are unpaired. Full 0–200-ns summaries and unpaired Welch intervals are provided in the source tables.

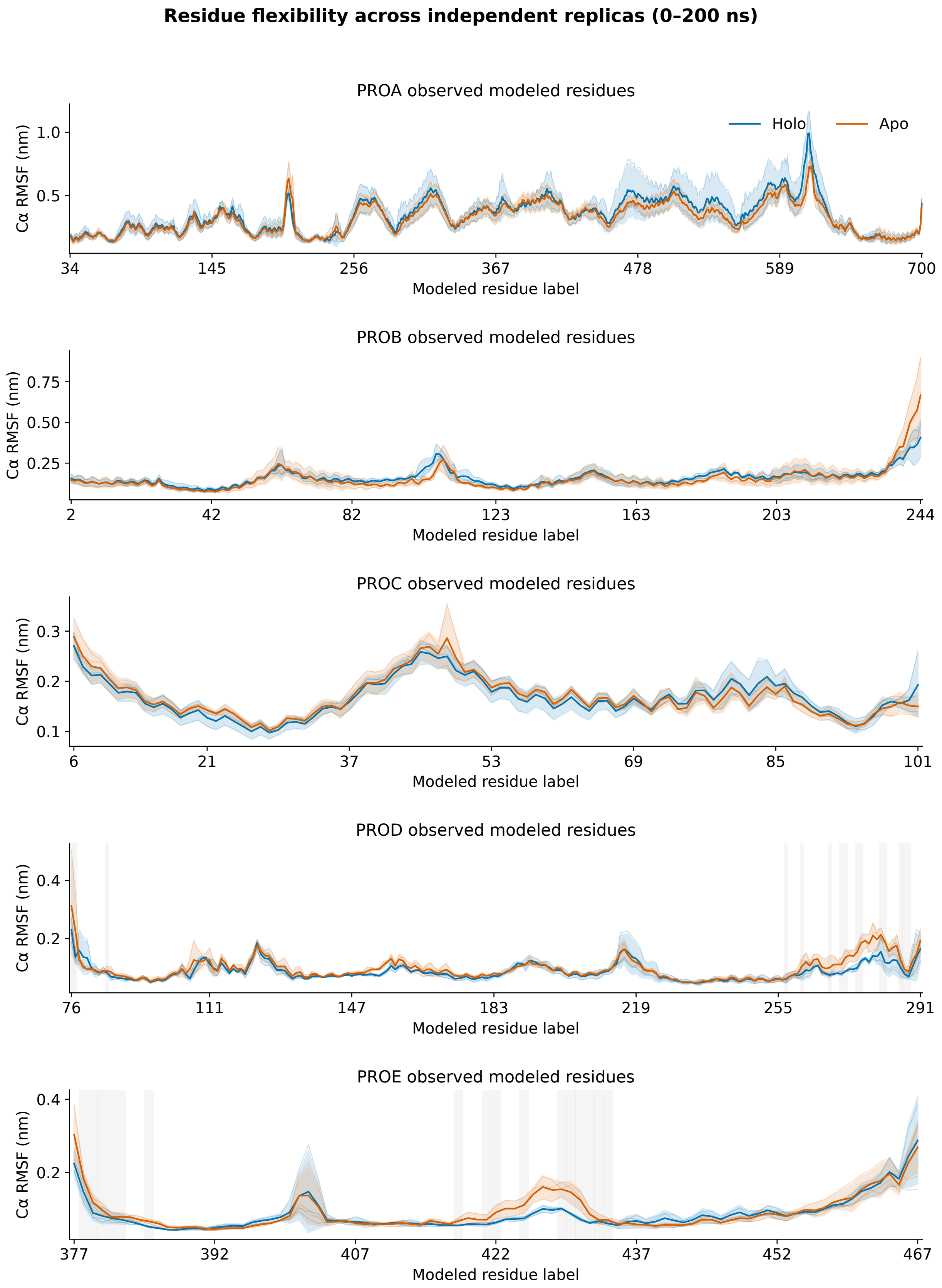

Figure S21. Residue flexibility over 200 ns. Cα RMSF is calculated separately for each run after unweighted PSEN1 fitting. Faint lines show individual runs, solid lines show equal-run means, and shading shows between-run standard deviations. Grey marks identify the predefined pocket. Only observed model residues are plotted. Source tables distinguish whole-trajectory RMSF from mean within-50-ns-block RMSF over 50–200 ns.

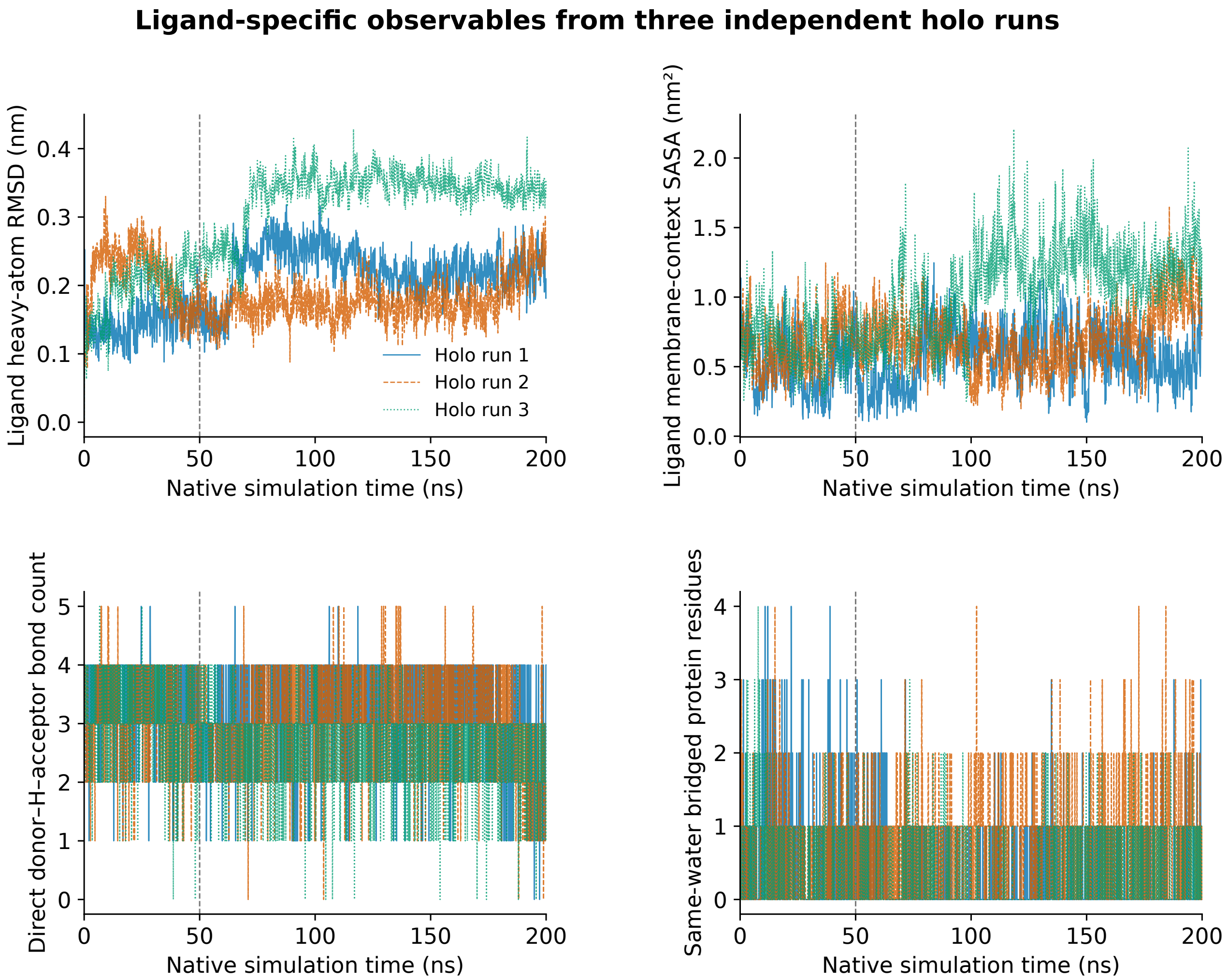

Figure S22. Ligand displacement, exposure and hydrogen-bond interactions. Ligand RMSD uses protein fitting without ligand self-alignment. Ligand SASA uses the membrane/glycan/ligand environment, RDKit van der Waals radii, a 1.4-Å probe and 256 deterministic surface points; 1,024-point resolution controls are retained separately. Direct hydrogen bonds require donor–acceptor distance ≤ 3.5 Å and donor–H–acceptor angle $\geq 150°$. A bridged residue requires two qualifying bonds through the same water oxygen in the same frame. Bond and bridged-residue counts have different units.

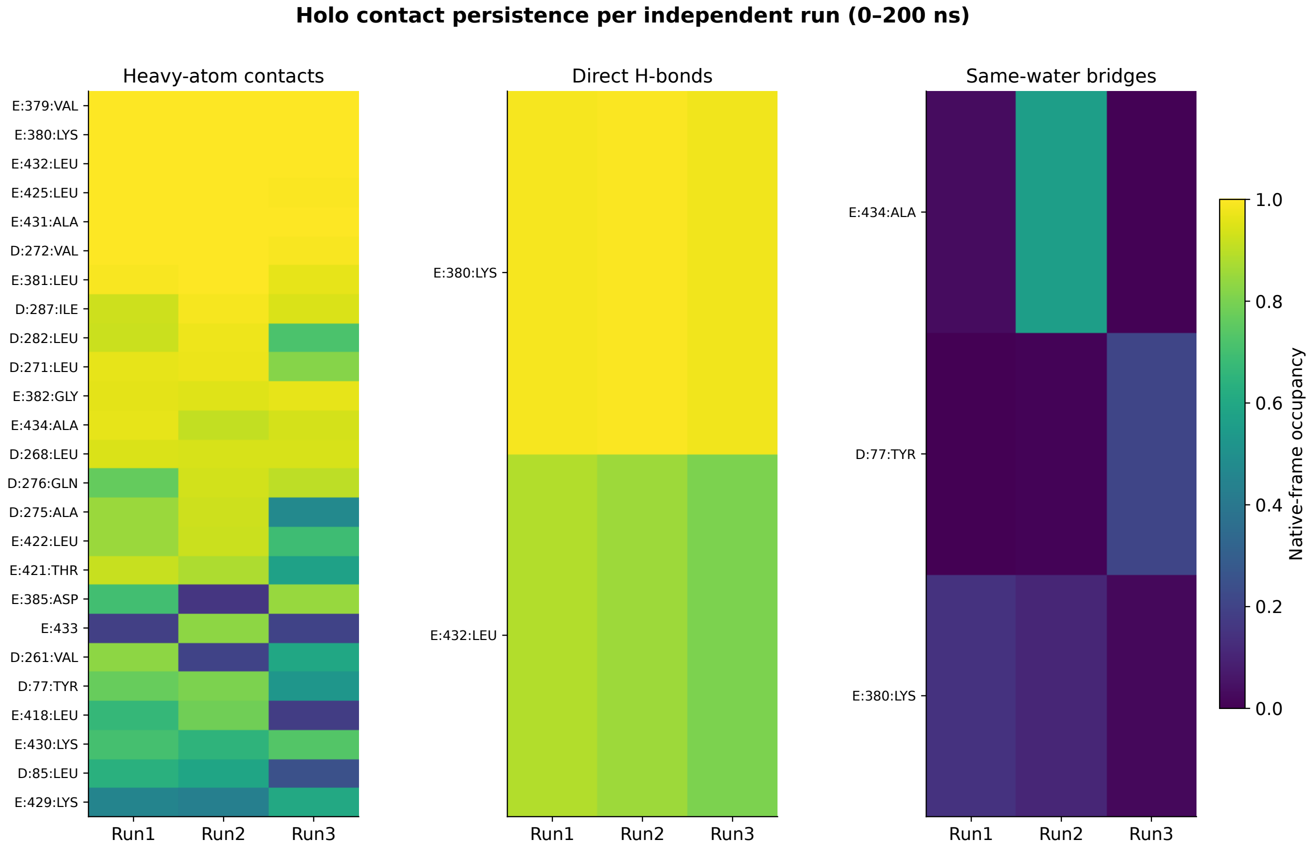

Figure S23. Residue-level ligand-contact occupancies. Source tables retain all 1,313 modelled protein residues and three event types. Each panel displays up to 25 residues with maximum occupancy $\geq 10\%$ in any run, ordered by that maximum. This is a descriptive display selection. Heavy-atom contacts require distance ≤ 4.5 Å. Occupancy is the fraction of native 10-ps frames with at least one qualifying event for the residue. Water bridges retain the same-frame, same-water definition in Methods S8.

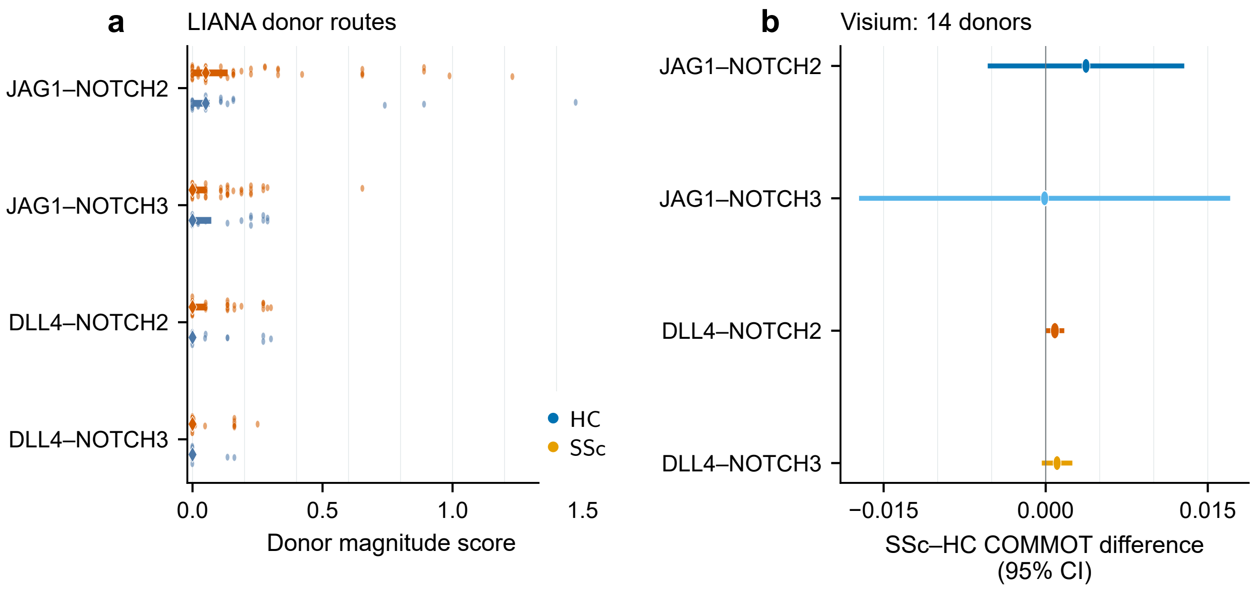

Figure S24. Donor communication scores and spatial disease contrasts. (a) LIANA+ magnitude scores for HC (blue) and SSc (orange): 133 eligible donors for JAG1 and 112 for DLL4 routes. Cohort-adjusted disease intervals include zero (BH $q = 0.714$–0.812). (b) Visium COMMOT SSc–HC differences and 95% intervals in 14 donor-labelled sections (10 SSc, 4 HC); disease tests use all 1,001 condition-label assignments. BH adjustment covers four routes. Raw donor scores and adjusted disease differences have distinct scales. Xenium contact enrichment and receiver detection are shown separately in main Figure 6; Methods S7 specifies the settings and null assumptions.

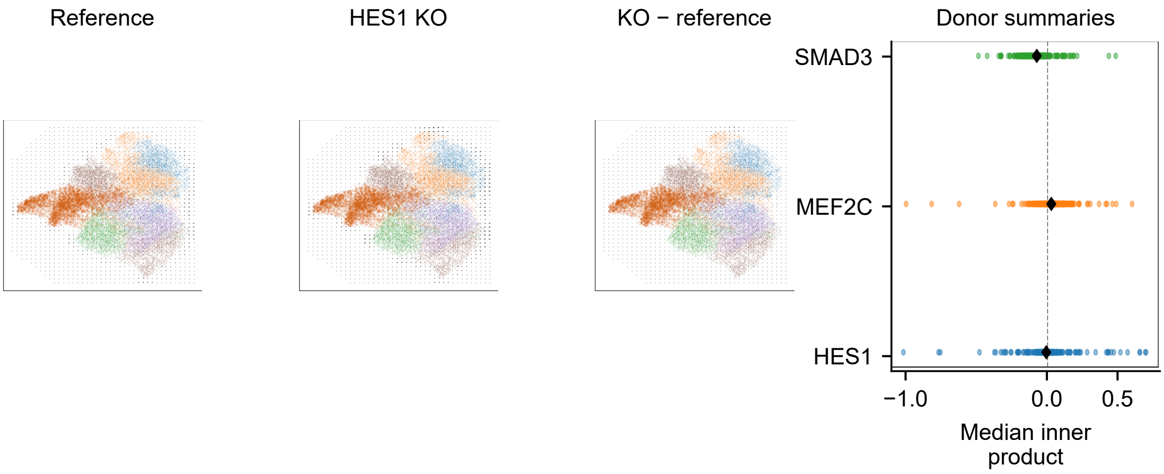

Figure S25. Fixed-model transition-field reconstruction and donor summaries. Left to right: reference transition field, HES1 knockout-weighted field, their exact difference, and fixed-field donor summaries for SMAD3, MEF2C and HES1. The reference and knockout fields use the archived 29,985-cell object and the same 40-by-40 display grid. The difference reproduces the exported calculation (maximum numerical discrepancy 1.55×10^{-15}). Donor points summarise the fixed mature-Myofibroblast field; diamonds and bars show medians and 10,000-donor-bootstrap 95% intervals. The HES1 summary includes 200 donors. These descriptive estimates condition on the fitted network and do not measure donor-specific network-refitting uncertainty, observed differentiation or causal effects. Methods S2 provides the developmental-reference and lineage definitions.

References

[1] Mollie E. Brooks, Kasper Kristensen, Koen J. van Benthem, Arni Magnusson, Casper W. Berg, Anders Nielsen, Hans J. Skaug, Martin Maechler, and Benjamin M. Bolker. glmmTMB balances speed and flexibility among packages for zero-inflated generalized linear mixed modeling. *The R Journal*, 9(2):378–400, 2017. doi: 10.32614/RJ-2017-066.

[2] Stefano Mangiola, Alexandra J. Roth-Schulze, Marie Trussart, Enrique Zozaya-Valdés, Mengyao Ma, Zijie Gao, Alan F. Rubin, Terence P. Speed, Heejung Shim, and Anthony T. Papenfuss. sccomp: Robust differential composition and variability analysis for single-cell data. *Proceedings of the National Academy of Sciences*, 120(33): e2203828120, 2023. doi: 10.1073/pnas.2203828120.

[3] Kenji Kamimoto, Blerta Stringa, Christy M. Hoffmann, Kunal Jindal, Lilianna Solnica-Krezel, and Samantha A. Morris. Dissecting cell identity via network inference and in silico gene perturbation. *Nature*, 614(7949):742–751, Feb 2023. ISSN 1476-4687. doi: 10.1038/s41586-022-05688-9.

[4] Hannah A. Pliner, Jonathan S. Packer, José L. McFaline-Figueroa, Darren A. Cusanovich, Riza M. Daza, Delasa Aghamirzaie, Sanjay Srivatsan, Xiaojie Qiu, Dana Jackson, Anna Minkina, Andrew C. Adey, Frank J. Steemers, Jay Shendure, and Cole Trapnell. Cicero predicts cis-regulatory dna interactions from single-cell chromatin accessibility data. *Molecular Cell*, 71(5):858–871.e8, Sept 2018. ISSN 1097-2765. doi: 10.1016/j.molcel.2018.06.044.

[5] Pau Badia-i Mompel, Jesús Vélez Santiago, Jana Braunger, Celina Geiss, Daniel Dimitrov, Sophia Müller-Dott, Petr Taus, Aurelien Dugourd, Christian H Holland, Ricardo O Ramirez Flores, and Julio Saez-Rodriguez. decoupler: ensemble of computational methods to infer biological activities from omics data. *Bioinformatics Advances*, 2(1):vbac016, Jan 2022. ISSN 2635-0041. doi: 10.1093/bioadv/vbac016.

[6] Sophia Müller-Dott, Eirini Tsirvouli, Miguel Vazquez, Ricardo O Ramirez Flores, Pau Badia-i Mompel, Robin Fallegger, Dénes Türei, Astrid Lægreid, and Julio Saez-Rodriguez. Expanding the coverage of regulons from high-confidence prior knowledge for accurate estimation of transcription factor activities. *Nucleic Acids Research*, 51(20):10934–10949, Oct 2023. ISSN 1362-4962. doi: 10.1093/nar/gkad841.

[7] Philipp Weiler, Marius Lange, Michal Klein, Dana Pe'er, and Fabian Theis. Cellrank 2: unified fate mapping in multiview single-cell data. *Nature Methods*, 21(7):1196–1205, June 2024. ISSN 1548-7105. doi: 10.1038/s41592-024-02303-9.

[8] F. Pedregosa, G. Varoquaux, A. Gramfort, V. Michel, B. Thirion, O. Grisel, M. Blondel, P. Prettenhofer, R. Weiss, V. Dubourg, J. Vanderplas, A. Passos, D. Cournapeau, M. Brucher, M. Perrot, and E. Duchesnay. Scikit-learn: Machine learning in Python. *Journal of Machine Learning Research*, 12:2825–2830, 2011.

[9] Carmen Bravo González-Blas, Seppe De Winter, Gert Hulselmans, Nikolai Hecker, Irina Matetovici, Valerie Christiaens, Suresh Poovathingal, Jasper Wouters, Sara Aibar, and Stein Aerts. Scenic+: single-cell multiomic inference of enhancers and gene regulatory networks. *Nature Methods*, 20(9):1355–1367, July 2023. ISSN 1548-7105. doi: 10.1038/s41592-023-01938-4.

[10] Andrew Kachites McCallum. MALLET: A machine learning for language toolkit. Software, 2002. URL `https://mimno.github.io/Mallet/index`. Official software citation; accessed 3 October 2026.

[11] Felix Mölder, Kim Philipp Jablonski, Brice Letcher, Michael B. Hall, Christopher H. Tomkins-Tinch, Vanessa Sochat, Jan Forster, Soohyun Lee, Sven O. Twardziok, Alexander Kanitz, Andreas Wilm, Manuel Holtgrewe, Sven Rahmann, Sven Nahnsen, and Johannes Köster. Sustainable data analysis with snakemake. *F1000Research*, 10:33, Apr 2021. ISSN 2046-1402. doi: 10.12688/f1000research.29032.2.

[12] Mark D. Robinson, Davis J. McCarthy, and Gordon K. Smyth. edgeR: a bioconductor package for differential expression analysis of digital gene expression data. *Bioinformatics*, 26(1):139–140, 2010. doi: 10.1093/bioinformatics/btp616.

[13] Vipul Singhal, Nigel Chou, Joseph Lee, Yifei Yue, Jinyue Liu, Wan Kee Chock, Li Lin, Yun-Ching Chang, Erica Mei Ling Teo, Jonathan Aow, Hwee Kuan Lee, Kok Hao Chen, and Shyam Prabhakar. BANKSY unifies cell typing and tissue domain segmentation for scalable spatial omics data analysis. *Nature Genetics*, 56:431–441, 2024. doi: 10.1038/s41588-024-01664-3.

[14] Sergio J. Rey and Luc Anselin. *PySAL: A Python Library of Spatial Analytical Methods*, pages 175–193. Springer Berlin Heidelberg, 2010. ISBN 9783642036477. doi: 10.1007/978-3-642-03647-7_11.

[15] Dénes Türei, Alberto Valdeolivas, Lejla Gul, Nicolàs Palacio-Escat, Michal Klein, Olga Ivanova, Márton Ölbei, Attila Gábor, Fabian Theis, Dezső Módos, Tamás Korcsmáros, and Julio Saez-Rodriguez. Integrated intra- and intercellular signaling knowledge for multicellular omics analysis. *Molecular Systems Biology*, 17(3):e9923, March 2021. ISSN 1744-4292. doi: 10.15252/msb.20209923.

[16] Robin Browaeys, Wouter Saelens, and Yvan Saeys. Nichenet: modeling intercellular communication by linking ligands to target genes. *Nature Methods*, 17(2):159–162, Feb 2020. ISSN 1548-7105. doi: 10.1038/s41592-019-0667-5.

[17] Daniel Dimitrov, Philipp Sven Lars Schäfer, Elias Farr, Pablo Rodriguez-Mier, Sebastian Lobentanzer, Pau Badia-i Mompel, Aurélien Dugourd, Jovan Tanevski, Ricardo Omar Ramirez Flores, and Julio Saez-Rodriguez. Liana+ provides an all-in-one framework for cell-cell communication inference. *Nature Cell Biology*, 26(9):1613–1622, 2024. doi: 10.1038/s41556-024-01469-w.

[18] Zixuan Cang, Yanxiang Zhao, Axel A. Almet, Adam Stabell, Raul Ramos, Maksim V. Plikus, Scott X. Atwood, and Qing Nie. Screening cell-cell communication in spatial transcriptomics via collective optimal transport. *Nature Methods*, 20(2):218–228, 2023. doi: 10.1038/s41592-022-01728-4.

[19] Dylan M. Cable, Evan Murray, Luli S. Zou, Aleksandr Goeva, Evan Z. Macosko, Fei Chen, and Rafael A. Irizarry. Robust decomposition of cell type mixtures in spatial transcriptomics. *Nature Biotechnology*, 40(4):517–526, 2022. doi: 10.1038/s41587-021-00830-w.

[20] John D. Hunter. Matplotlib: A 2d graphics environment. *Computing in Science & Engineering*, 9(3):90–95, 2007. doi: 10.1109/MCSE.2007.55.

[21] Charles R. Harris, K. Jarrod Millman, Stéfan J. van der Walt, Ralf Gommers, Pauli Virtanen, David Cournapeau, Eric Wieser, Julian Taylor, Sebastian Berg, Nathaniel J. Smith, Robert Kern, Matti Picus, Stephan Hoyer, Marten H. van Kerkwijk, Matthew Brett, Allan Haldane, Jaime Fernández del Río, Mark Wiebe, Pearu Peterson, Pierre Gérard-Marchant, Kevin Sheppard, Tyler Reddy, Warren Weckesser, Hameer Abbasi, Christoph Gohlke, and Travis E. Oliphant. Array programming with NumPy. *Nature*, 585(7825):357–362, 2020. doi: 10.1038/s41586-020-2649-2.

[22] Wes McKinney. Data structures for statistical computing in Python. In *9th Python in Science Conference*, pages 56–61, 2010. doi: 10.25080/Majora-92bf1922-00a.

[23] Artifex. PyMuPDF 1.27.2.3. Computer software, 2026. URL `https://github.com/pymupdf/PyMuPDF/releases/tag/1.27.2.3`. Version 1.27.2.3; accessed 3 October 2026.

[24] pypdf Contributors. pypdf 6.11.0. Computer software, 2026. URL `https://github.com/py-pdf/pypdf/releases/tag/6.11.0`. Version 6.11.0; accessed 3 October 2026.

[25] R Core Team. *R: A Language and Environment for Statistical Computing*. R Foundation for Statistical Computing, Vienna, Austria, 2025. URL `https://www.R-project.org/`.

[26] Skipper Seabold and Josef Perktold. statsmodels: Econometric and statistical modeling with Python. In *9th Python in Science Conference*, 2010. URL `https://www.statsmodels.org/stable/index.html`.

[27] Pauli Virtanen, Ralf Gommers, Travis E. Oliphant, Matt Haberland, Tyler Reddy, David Cournapeau, Evgeni Burovski, Pearu Peterson, Warren Weckesser, Jonathan Bright, Stéfan J. van der Walt, Matthew Brett, Joshua Wilson, K. Jarrod Millman, Nikolay Mayorov, Andrew R. J. Nelson, Eric Jones, Robert Kern, Eric Larson, C. J. Carey, İlhan Polat, Yu Feng, Eric W. Moore, Jake VanderPlas, Denis Laxalde, Josef Perktold, Robert Cimrman, Ian Henriksen, E. A. Quintero, Charles R. Harris, Anne M. Archibald, Antônio H. Ribeiro, Fabian Pedregosa, Paul van Mulbregt, and SciPy 1.0 Contributors. SciPy 1.0: Fundamental algorithms for scientific computing in Python. *Nature Methods*, 17(3):261–272, 2020. doi: 10.1038/s41592-019-0686-2.

[28] Xuefei Guo, Haotian Li, Xiaoli Lu, Hao Liu, Kaicheng U, Chuangye Yan, Jianlin Lei, Jing Huang, Rui Zhou, and Yigong Shi. Structural basis of human γ-secretase inhibition by anticancer clinical compounds. *Nature Structural & Molecular Biology*, 32(4):719–728, 2025. doi: 10.1038/s41594-024-01439-8.

[29] G. Yang, R. Zhou, X. Guo, C. Yan, J. Lei, and Y. Shi. Structural basis of gamma-secretase inhibition and modulation by small molecule drugs. *Cell*, 184:521–533.e14, 2021. doi: 10.1016/j.cell.2020.11.049.

[30] J. Huang et al. CHARMM36m: an improved force field for folded and intrinsically disordered proteins. *Nature Methods*, 14:71–73, 2017. doi: 10.1038/nmeth.4067.

[31] K. Vanommeslaeghe et al. CHARMM general force field: A force field for drug-like molecules compatible with the charmm all-atom additive biological force fields. *Journal of Computational Chemistry*, 31:671–690, 2010. doi: 10.1002/jcc.21367.

[32] K. Vanommeslaeghe, E. P. Raman, and A. D. MacKerell. Automation of the CHARMM general force field (CGenFF) II: Assignment of bonded parameters and partial atomic charges. *Journal of Chemical Information and Modeling*, 52(12):3155–3168, 2012. doi: 10.1021/ci3003649.

[33] Seonghoon Kim, Jumin Lee, Sunhwan Jo, III Brooks, Charles L., Hui Sun Lee, and Wonpil Im. CHARMM-GUI ligand reader and modeler for CHARMM force field generation of small molecules. *Journal of Computational Chemistry*, 38(21):1879–1886, 2017. doi: 10.1002/jcc.24829.

[34] S. Jo, T. Kim, V. G. Iyer, and W. Im. CHARMM-GUI: a web-based graphical user interface for CHARMM. *Journal of Computational Chemistry*, 29(11):1859–1865, 2008. doi: 10.1002/jcc.20945.

[35] J. Lee et al. CHARMM-GUI input generator for NAMD, GROMACS, AMBER, OpenMM, and CHARMM/OpenMM simulations using the CHARMM36 additive force field. *Journal of Chemical Theory and Computation*, 12:405–413, 2016. doi: 10.1021/acs.jctc.5b00935.

[36] E. L. Wu et al. CHARMM-GUI membrane builder toward realistic biological membrane simulations. *Journal of Computational Chemistry*, 35(27):1997–2004, 2014. doi: 10.1002/jcc.23702.

[37] B. R. Brooks et al. CHARMM: The biomolecular simulation program. *Journal of Computational Chemistry*, 30 (10):1545–1614, 2009. doi: 10.1002/jcc.21287.

[38] Mark James Abraham, Teemu Murtola, Roland Schulz, Szilárd Páll, Jeremy C. Smith, Berk Hess, and Erik Lindahl. GROMACS: High performance molecular simulations through multi-level parallelism from laptops to supercomputers. *SoftwareX*, 1–2:19–25, 2015. doi: 10.1016/j.softx.2015.06.001.

[39] David A. Case, Hasan Metin Aktulga, Kellon Belfon, David S. Cerutti, G. Andrés Cisneros, Vinícius Wilian D. Cruzeiro, Negin Forouzesh, Timothy J. Giese, Andreas W. Götz, Holger Gohlke, et al. Ambertools. *Journal of Chemical Information and Modeling*, 63:6183–6191, 2023. doi: 10.1021/acs.jcim.3c01153. URL `https://doi.org/10.1021/acs.jcim.3c01153`.

[40] F. Alexander Wolf, Philipp Angerer, and Fabian J. Theis. Scanpy: large-scale single-cell gene expression data analysis. *Genome Biology*, 19(1):15, Feb 2018. ISSN 1474-760X. doi: 10.1186/s13059-017-1382-0.

[41] Isaac Virshup, Sergei Rybakov, Fabian J. Theis, Philipp Angerer, and F. Alexander Wolf. anndata: Access and store annotated data matrices. *Journal of Open Source Software*, 9(101):4371, 2024. doi: 10.21105/joss.04371. URL `https://doi.org/10.21105/joss.04371`.

[42] Matthew E. Ritchie, Belinda Phipson, Di Wu, Yifang Hu, Charity W. Law, Wei Shi, and Gordon K. Smyth. limma powers differential expression analyses for rna-sequencing and microarray studies. *Nucleic Acids Research*, 43 (7):e47, 2015. doi: 10.1093/nar/gkv007.

[43] M. F. Adasme, K. L. Linnemann, S. N. Bolz, F. Kaiser, S. Salentin, V. J. Haupt, and M. Schroeder. PLIP 2021: expanding the scope of the protein–ligand interaction profiler to DNA and RNA. *Nucleic Acids Research*, 49 (W1):W530–W534, 2021. doi: 10.1093/nar/gkab294.

[44] Schrödinger, LLC. The PyMOL molecular graphics system, version 3.1.1, 2024. URL `https://www.pymol.org/`.

[45] N. Michaud-Agrawal, E. J. Denning, T. B. Woolf, and O. Beckstein. MDAnalysis: A toolkit for the analysis of molecular dynamics simulations. *Journal of Computational Chemistry*, 32:2319–2327, 2011. doi: 10.1002/jcc.21787.

[46] R. J. Gowers et al. MDAnalysis: A python package for the rapid analysis of molecular dynamics simulations. In *Proceedings of the 15th Python in Science Conference*, pages 98–105, 2016. doi: 10.25080/Majora-629e541a-00e.
